# Efficacy and safety of fluoroquinolones in the treatment of human brucellosis: A systematic review and meta-analysis

**DOI:** 10.1371/journal.pntd.0013822

**Published:** 2026-10-23

**Authors:** Mohsen Mohammadi-Sartang, Nastaran Rafieipour, Shahla Afrasiabian, Roya Mahmoudi, Yousef Moradi, Mehdi Karimi, Karo Servatyari

**Affiliations:** 1 Department of Clinical Nutrition, School of Nutrition and Food Sciences, Shiraz University of Medical Sciences, Shiraz, Iran; 2 Department of Nutrition, Islamic Azad University, Rasht Branch, Rasht, Iran; 3 Zoonoses Research Center, Research Institute for Health Development, Kurdistan University of Medical Sciences, Sanandaj, Iran; 4 Nutrition Research Center, School of Nutrition and Food Sciences, Tabriz University of Medical Sciences, Tabriz, Iran; 5 Department of Diagnostics and Intervention, Umeå University, Umeå, Sweden; 6 Faculty of Medicine, Bogomolets National Medical University, Kyiv, Ukraine; University of Calgary, CANADA

## Abstract

**Introduction:**

Brucellosis is a major zoonotic infection worldwide, with the highest burden reported in the Mediterranean region. The therapeutic role of fluoroquinolones in this disease continues to be controversial. This systematic review and meta-analysis aimed to evaluate the efficacy and safety of fluoroquinolone-containing regimens compared with standard non-fluoroquinolone regimens for the treatment of human brucellosis.

**Methods:**

In accordance with PRISMA recommendations, this systematic review and meta-analysis assessed the efficacy and safety of fluoroquinolone-based regimens for treating brucellosis. It was prospectively registered in PROSPERO (CRD420251142019). A comprehensive search of PubMed, Scopus, Web of Science, and the Cochrane Library was carried out, including studies published through August 2025. Randomized controlled trials comparing fluoroquinolones with conventional therapies (such as doxycycline with streptomycin or rifampicin) were included. Therapeutic failure, relapse, adverse events, and time to defervescence were the main outcomes. To compute pooled risk ratios and mean differences with 95%CIs, data were analyzed using STATA v18. The GRADE method and the RoB 2 tool were used to evaluate the risk of bias and the certainty of the evidence.

**Results:**

The analysis comprised 9 clinical studies with 602 brucellosis patients. When compared to standard regimens, fluoroquinolone-based regimens were linked to a significantly higher risk of therapeutic failure (RR = 2.11, 95% CI: 1.26–3.51; P = 0.004) and a higher, though not statistically significant, risk of relapse (RR = 1.88, 95% CI: 0.94–3.74; P = 0.07). No statistically significant between-study heterogeneity was observed for either outcome (I² = 0%). Subgroup analyses showed a significantly increased risk of therapeutic failure in patients with uncomplicated brucellosis and in studies with treatment durations >8 weeks, although between-subgroup differences were not statistically significant. On the other hand, no statistically significant difference in adverse events was observed between fluoroquinolone-based and standard regimens under the random-effects model (RR = 0.62, 95% CI: 0.29–1.33; P = 0.22). Although the point estimate favored fluoroquinolone-based regimens, the confidence interval included the null effect**.** Fluoroquinolone-based regimens were also associated with a significantly longer time to defervescence compared with standard regimens (MD = 1.20 days, 95% CI: 0.66–1.74; P < 0.001). The certainty of evidence was moderate for therapeutic failure, and time to defervescence, and low for relapse and adverse events according to the GRADE evaluation.

**Conclusion:**

Compared with conventional doxycycline-based combinations, fluoroquinolone-based regimens were associated with a higher risk of therapeutic failure and longer time to defervescence, while the overall risk of relapse did not differ significantly. No significant difference in adverse events was observed with the random-effects model, although the point estimate favored fluoroquinolone-based regimens. Overall, these findings do not support the routine use of fluoroquinolones as first-line therapy for adult brucellosis. Further well-designed randomized controlled trials are needed.

## Author summary

Brucellosis is a zoonotic infection in which treatment failure and relapse remain important concerns. We compared fluoroquinolone-based regimens with standard treatments for human brucellosis. Fluoroquinolone-based regimens were associated with more treatment failures and a longer time to defervescence, while the overall risk of relapse was not significantly different. No significant difference in adverse events was found with the random-effects analysis, although the results tended to favor fluoroquinolones. Overall, these findings do not support the routine use of fluoroquinolones as first-line therapy. Established doxycycline-based combination regimens remain preferred treatment options**.**

## Introduction

Brucellosis, a zoonotic infection historically termed Malta fever, is a serious communicable zoonosis of great importance that is prevalent in various regions of the world, particularly in the Middle East and Iran [1]. Humans are infected by bacteria of the genus Brucella through contact with contaminated animals, contact with their body fluids, or intake of unpasteurized milk and its derivatives, and develop a pervasive systemic infection [2].

Between 2015 and 2016, bovine brucellosis prevalence in Kuwait and Jordan ranged between 7.2% and 18.1%. In comparison, during 2012–2014, the prevalence was much lower around 3% to 3.1%in Iraq and Iran. In Iran, the prevalence of caprine brucellosis due to Brucella melitensis was estimated at 3.5% in 2014. At present, the test-and-slaughter control strategy is mainly used in countries where disease prevalence remains below 2% [1].

Effective control of brucellosis has invariably provided a significant challenge to the field of medicine, in part due to Brucella’s ability to endure within host cells, such as macrophages and reticuloendothelial cells, and to promote latent infection reservoirs. Their intracellular environment impairs the efficacy of many conventional antibiotics to reach an effective concentration, and the resultant occurrence of relapse following successful initial treatment is a significant concern. Brucella invades host cells, suppresses lysosomal fusion, and moves to the endoplasmic reticulum to replicate. Its survival within cells relies on the BvrS/BvrR regulatory system, VirB type IV secretion machinery, and a complete lipopolysaccharide (LPS) layer [3].

A meta-analytic evaluation by Big et al. indicated that fluoroquinolones retain considerable efficacy against *Brucella* spp., with resistance reported infrequently (1.6–4.6%). The study further highlighted their potential role as a safe therapeutic alternative in situations involving disease recurrence or adverse events related to standard treatment protocols. Because of their intracellular activities, low toxicity to the kidneys, and relapse prevention potential, fluoroquinolones are a suitable choice for patients who develop complications from other agents [4].

In contrast, a domestic systematic review titled “Antibiotic susceptibility of Brucella spp. isolated from human specimens” showed alarming results. Although the first focus of the study was on overseas data, the study results show that there are strains of Brucella whose susceptibility to quinolones, such as ciprofloxacin, is reduced [5].

While the usual therapy for brucellosis involves regimens of doxycycline and rifampin or doxycycline and streptomycin [6], fluoroquinolones have been suggested as an alternative or adjunctive mode of treatment in selected cases. This is because of various beneficial factors, including adequate oral absorption, acceptable tolerability, and intracellular penetration [6].

Despite encouraging results from some laboratory and clinical investigations, the overall body of evidence remains limited and does not currently warrant the recommendation of quinolones as initial therapy for patients with human brucellosis [7].

However, the possible benefits associated with fluoroquinolones should be evaluated against the proven effectiveness of the conventional drug regimen since the treatment of brucellosis involves more than improving the symptoms; it is necessary to prevent recurrence of the disease [8,9].

Newer agents of the fluoroquinolone group possess enhanced activity against gram-negative bacilli and selected Gram-positive pathogens, particularly, Streptococcus pneumoniae, Staphylococcus aureus, and selected anaerobic organisms. These agents possess a longer half-life, are administered once daily at a fixed dose, and selected agents are eliminated through non-renal clearance. Clinical trials have established positive efficacy for a range of infections relative to standard treatment regimens; however, a direct comparison of various fluoroquinolone agents has not yet been conducted [10].

Despite favorable oral bioavailability, intracellular penetration, and tolerability, the clinical efficacy of fluoroquinolone-containing regimens for brucellosis remains uncertain. Previous studies have reported inconsistent findings and were often limited by small sample sizes and heterogeneous regimens. Furthermore, differences in the fluoroquinolone used, treatment combinations, duration of therapy, and clinical outcomes assessed have made it difficult to draw firm conclusions regarding their comparative efficacy and safety [7,11,12].

Therefore, we aimed to compare the efficacy and safety of fluoroquinolone-containing regimens with standard non-fluoroquinolone regimens for the treatment of human brucellosis.

## Methods

### Ethical approval

Ethical approval was not required as this study used data from previously published research.

### Protocol and registration

The Preferred Reporting Items for Systematic Reviews and Meta-Analyses (PRISMA) 2020 criteria were followed in conducting this systematic review and meta-analysis [9](S1 Checklist). Prior to the start of the investigation, the protocol was prospectively registered in the International Prospective Register of Systematic Reviews (PROSPERO) database (registration number: CRD420251142019), guaranteeing methodological rigor and transparency.

### Search strategy

A comprehensive and systematic literature search was performed across PubMed, Scopus, Web of Science, and the Cochrane Library from database inception to August 2, 2025. The search strategy incorporated both Medical Subject Headings (MeSH) and free-text terms related to brucellosis and fluoroquinolones. Key terms included “brucellosis”, “Brucella”, “Malta fever”, “Gibraltar fever”, “undulant fever”, and “Cyprus fever”, combined with “fluoroquinolone”, “ciprofloxacin”, “ofloxacin”, “levofloxacin”, and “moxifloxacin”, along with outcome- and design-related terms such as “treatment failure”, “relapse”, “treatment outcome”, “therapy”, “clinical trial”, and “randomized controlled trial”. The search strategy was adapted for each database to maximize sensitivity. In addition, reference lists of all included studies and relevant review articles were manually screened to identify additional eligible studies, and inclusion was restricted to English-language publications (S1 Table).

### Eligibility criteria

Eligibility was defined a priori using PICO-based criteria, including study design, population, intervention, comparator, and outcomes. Specifically, randomized controlled trials (RCTs) involving patients diagnosed with brucellosis using clinical, serological, or microbiological criteria were included. Eligible interventions consisted of fluoroquinolone-based treatment regimens administered either as monotherapy or in combination with other antimicrobial agents, while comparators included standard recommended regimens such as doxycycline plus rifampicin or doxycycline plus streptomycin. Studies were required to report at least one relevant outcome, including therapeutic failure, relapse, adverse events, or time to defervescence. Therapeutic failure was defined as the lack of clinical improvement or worsening of symptoms during treatment, while relapse referred to the recurrence of symptoms or laboratory-confirmed infection following completion of therapy and initial cure. Adverse events were defined as any reported drug-related side effects, particularly those leading to treatment discontinuation or dose adjustment, and time to defervescence was defined as the number of days from treatment initiation to sustained resolution of fever. Studies were excluded if they were case series, retrospective designs, review articles, or if they provided insufficient data to calculate effect estimates.

### Study selection

All retrieved records were independently screened by two reviewers using a two-stage process, involving initial title and abstract screening followed by full-text assessment of potentially eligible studies. Any disagreements between reviewers were resolved through discussion and consensus to ensure accurate study selection. The reasons for exclusion at the full-text assessment stage are provided in S2 Table.

### Data extraction

To guarantee consistency and accuracy of the extracted data, two reviewers separately carried out the data extraction process using a standardized data collecting form. Study characteristics (first author, publication year, country, and study design), participant characteristics (sample size, mean age, and sex distribution), intervention and comparator regimen details (drug combinations, dosage, and duration), follow-up period, and all predefined outcomes of interest were all extracted. Effect sizes, such as averages, standard deviations, or event counts, were computed or estimated using accepted statistical techniques when outcome data were not directly reported. Discussion was used to settle any disagreements between reviewers. The detailed data-extraction information for the included studies is provided in the Supporting Information (S3 Table).

The primary objective of this systematic review and meta-analysis was to compare treatment success between fluoroquinolone-containing regimens and standard non-fluoroquinolone regimens in patients with human brucellosis. Secondary outcomes included relapse, overall treatment failure, adverse events, and treatment discontinuation due to adverse events. Disagreements between reviewers were resolved through discussion. The extracted data used for the quantitative synthesis are provided in the Supporting Information (S3 Table).

### Risk of bias assessment

The Cochrane Risk of Bias 2 (RoB 2) tool was used to evaluate the methodological quality of the included randomized trials [10]. This test assesses five domains: measurement of outcomes, bias resulting from the randomization procedure, deviations from intended treatments, missing outcome data, and selection of reported results. Based on these evaluations, each study was given an overall risk of bias judgment. Each domain was assessed as low risk of bias, moderate concerns, or high risk of bias. Risk of bias was assessed for each included study using the prespecified criteria, with detailed domain-level judgments presented in S4 Table.

### Certainty of evidence

The degree of certainty of the evidence supporting each result was evaluated using the Grading of Recommendations Assessment, Development, and Evaluation (GRADE) method [12]. This technique considers five primary categories: risk of bias, inconsistency, indirectness, imprecision, and publication bias. Based on these criteria, the overall certainty of evidence for each outcome was classified as strong, moderate, low, or extremely low. The certainty of evidence ranged from low to moderate across the evaluated outcomes (S5 Table).

### Statistical analysis

STATA software, version 18, was used to perform meta-analyses. Pooled risk ratios with 95% confidence intervals (CIs) were computed for binary outcomes, such as treatment failure, relapse, and adverse events; mean differences with 95% CIs were utilized for continuous outcomes, such as time to defervescence. Fixed effect model was chosen since the studies involved in this research had adequate homogeneity in the aspect of population, interventions, comparisons, and outcomes. Cochran’s Q test was used to evaluate statistical heterogeneity, and the I2 statistic was used to quantify it; values above 50% indicated significant heterogeneity. The kind of fluoroquinolone regimen, length of therapy, disease type (acute versus complex brucellosis), location, sample size, and length of follow-up were all taken into consideration when performing predefined subgroup analyses. The robustness of the pooled estimates was assessed by leave-one-out sensitivity analyses. Begg’s rank correlation test, Egger’s regression test, were used to evaluate publication bias.

## Results

After the removal of 54 duplicates, 177 records remained. Of these, 104 were excluded by title/abstract screening, leaving 73 reports sought for retrieval. Thirty reports could not be retrieved. Of the 43 full-text articles assessed for eligibility, 34 was excluded (8 case reports,14 reviews, 10 invitro or in vivo studies, 2 prospective comparative) resulting in the final inclusion of 9 clinical trials [11,13–20] (Fig 1).

**Fig 1:**
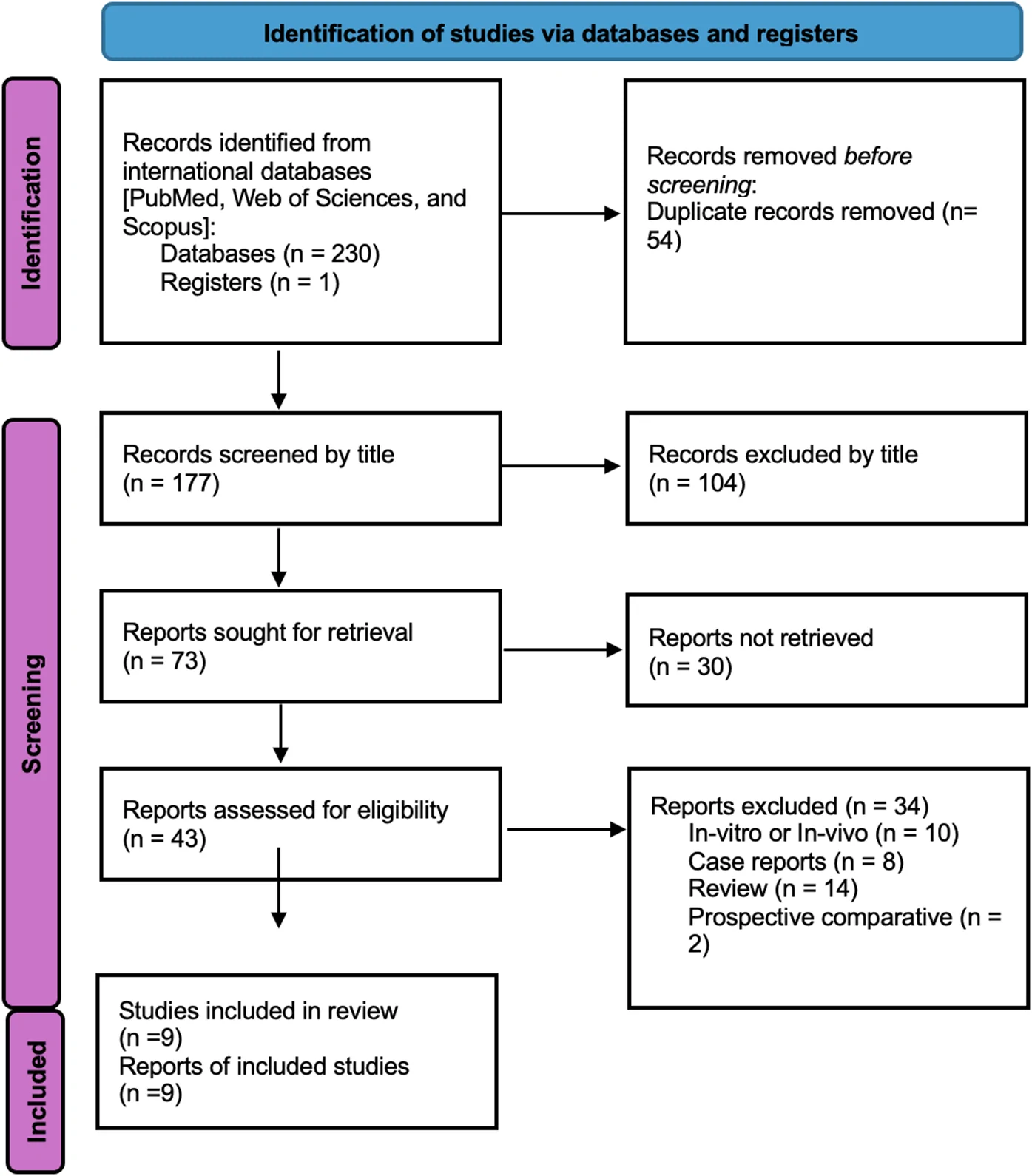
PRISMA flow diagram of study selection.

Tables 1 and S2 summarizes the baseline characteristics of the included studies. Nine studies conducted between 1990 and 2014 across several countries, predominantly Iran and Turkey, with additional studies from Israel and Albania, were included. The sample sizes varied substantially, ranging from 10 to 178 participants (total: 602 patients). The mean age of the enrolled patients was generally in the third to fourth decade of life, with most studies including both men and women. Overall, there was a balanced sex distribution across the intervention and control groups, with no major sex-related imbalances reported. All included trials evaluated adult patients with brucellosis, most commonly acute uncomplicated disease, although some studies also enrolled patients with osteoarticular involvement or complicated forms, such as brucellar spondylitis. The intervention arms predominantly consisted of fluoroquinolone-based combinations, mainly ciprofloxacin or ofloxacin (administered orally in standard doses), combined with rifampicin or doxycycline. Control groups typically received standard regimens, most frequently doxycycline plus rifampicin or doxycycline combined with streptomycin. Treatment durations ranged from short courses of 6 weeks or 45 days to longer regimens of 3 months, depending on the trial design and patient population. Most of the included studies employed randomized parallel-group controlled trial designs. In summary, the included trials represent a relatively young adult population with acute brucellosis, with balanced demographic characteristics between groups, and investigated diverse fluoroquinolone-based regimens compared to conventional standard-of-care therapies over variable treatment durations.

**Table 1:** Demographic characteristics of the included studies.

First author/ year	Location	Mean age years (I/C)	Study Design	Sample size Total(I/C)	Female/Male (I/C)	Intervention Group	Control Group	Duration	Population
Murat Akova/1993 [11]	Ankara/Turkey	(34.4 ± 14.3)/(37.8 ± 15.1)	Parallel RCT	61(30/31)	(16/14)/(14/17)	Ofloxacin (400 mg once daily) Rifampicin (600 mg once daily)	Doxycycline (200 mg once daily) Rifampicin (600 mg once daily)	6 weeks	Adults with acute uncomplicated brucellosis
Keramat/2009 [17]	Hamedan/Iran	39.67/43.66	Parallel RCT	178(61/117)	61/117	Ciprofloxacin (500 mg PO twice daily) Rifampicin (600 mg once daily) OR Ciprofloxacin (500 mg PO twice daily) Doxycycline (200 mg once daily)	Doxycycline (200 mg once daily) Rifampicin (600 mg once daily)	8-12 weeks	Adults with acute uncomplicated brucellosis
Lang/1990 [18]	Israel	Range (18–58)	Parallel RCT	10(6/4)	(3/3)/(3/1)	Ciprofloxacin (500 mg PO twice daily)	Doxycycline (200 mg once daily) Rifampicin (600 mg once daily)	6 weeks	Adults with acute uncomplicated brucellosis
Kalo/1996 [15]	Tirana, Albania	Total (31.8 ± 13.5)	Parallel RCT	24(12/12)	10/14	Ciprofloxacin (500 mg PO twice daily) Doxycycline (200 mg once daily)	Doxycycline (200 mg once daily) Rifampicin (600 mg once daily)	6 weeks	Adults with acute uncomplicated brucellosis
Didgar/2012 [19]	Arak/Iran	(38.6 ± 16.7)/(35.4 ± 16.4)	Randomized controlled trial (triple blind)	80(40/40)	20/60	Ciprofloxacin (500 mg PO twice daily) Doxycycline (200 mg once daily)	Doxycycline (200 mg once daily) Rifampicin (600 mg once daily)	8 weeks	Adults with acute uncomplicated brucellosis
Alikhani/2007 [14]	Tonekabon/Iran	Range (15–80)	Single blinded Parallel RCT	78(38/40)	NR	Ofloxacin (400 mg once daily) Rifampicin (600 mg once daily) Streptomycin (750–1000 mg IM daily)	Doxycycline (200 mg once daily) Rifampicin (600 mg once daily) Streptomycin (750– 1000 mg IM daily)	3 months	Adults with acute uncomplicated brucellosis
Agalar/1999 [13]	Ankara/Turkey	(37.8 ± 13.9)/(37.1 ± 15.8)	Parallel RCT	40(20/20)	(12/8)/(7/13)	Ciprofloxacin (500 mg PO twice daily) Rifampicin (600 mg once daily)	Doxycycline (200 mg once daily) Rifampicin (600 mg once daily)	45 days	Hospitalized brucellosis patients
Karabay/2004 [16]	Duzce/Turkey	Range(18–60/24–61)	Parallel RCT	29(15/14)	(2/13)/(3/11)	Ofloxacin (400 mg once daily) Rifampicin (600 mg once daily)	Doxycycline (200 mg once daily) Rifampicin (600 mg once daily)	45 days	Hospitalized brucellosis patients
Bayindir/2003 [20]	Malatya/Turkey	Overall: 40.5	Parallel RCT	102(19/83)	46/56	Ofloxacin (400 mg once daily) Rifampicin (600 mg once daily)	ST = 20 (Streptomycin 1g IM + Tetracycline 500 mg PO q6h) SD = 21(Streptomycin 1g IM + Doxycycline 100 mg PO q12h) DR = 20 (Doxycycline 100 mg PO q12h + Rifampicin 15 mg/kg PO qd) SDR = 22 (Streptomycin 1g IM + Doxycycline 100 mg PO q12h + Rifampicin 15 mg/kg PO qd)	45 days	Brucellar spondylitis

### Therapeutic failure.

In a pooled analysis of 8 studies, fluoroquinolone-based regimens were associated with a significantly higher risk of therapeutic failure compared with standard treatment regimens. The overall pooled RR was 2.11 (95% CI: 1.26–3.51; p = 0.004), indicating that patients receiving fluoroquinolone-based combinations had more than twice the risk of treatment failure. No statistically significant between-study heterogeneity was observed (Cochran’s Q = 3.02, df = 7, p = 0.88; I^2^ = 0%). In leave one out sensitivity analysis, pooled RRs ranged from 1.80 to 2.26, and the direction of the effect remained consistent. The pooled effect remained statistically significant after excluding each of the other seven studies individually. On leaving out Keramat (2009), however, the pooled effect was no longer statistically significant (RR = 1.80, 95% CI: 0.94-3.43; p = 0.076). Thus, although the direction of the effect remained consistent, statistical significance was sensitive to the exclusion of Keramat (2009) (Fig 2).

**Fig 2:**
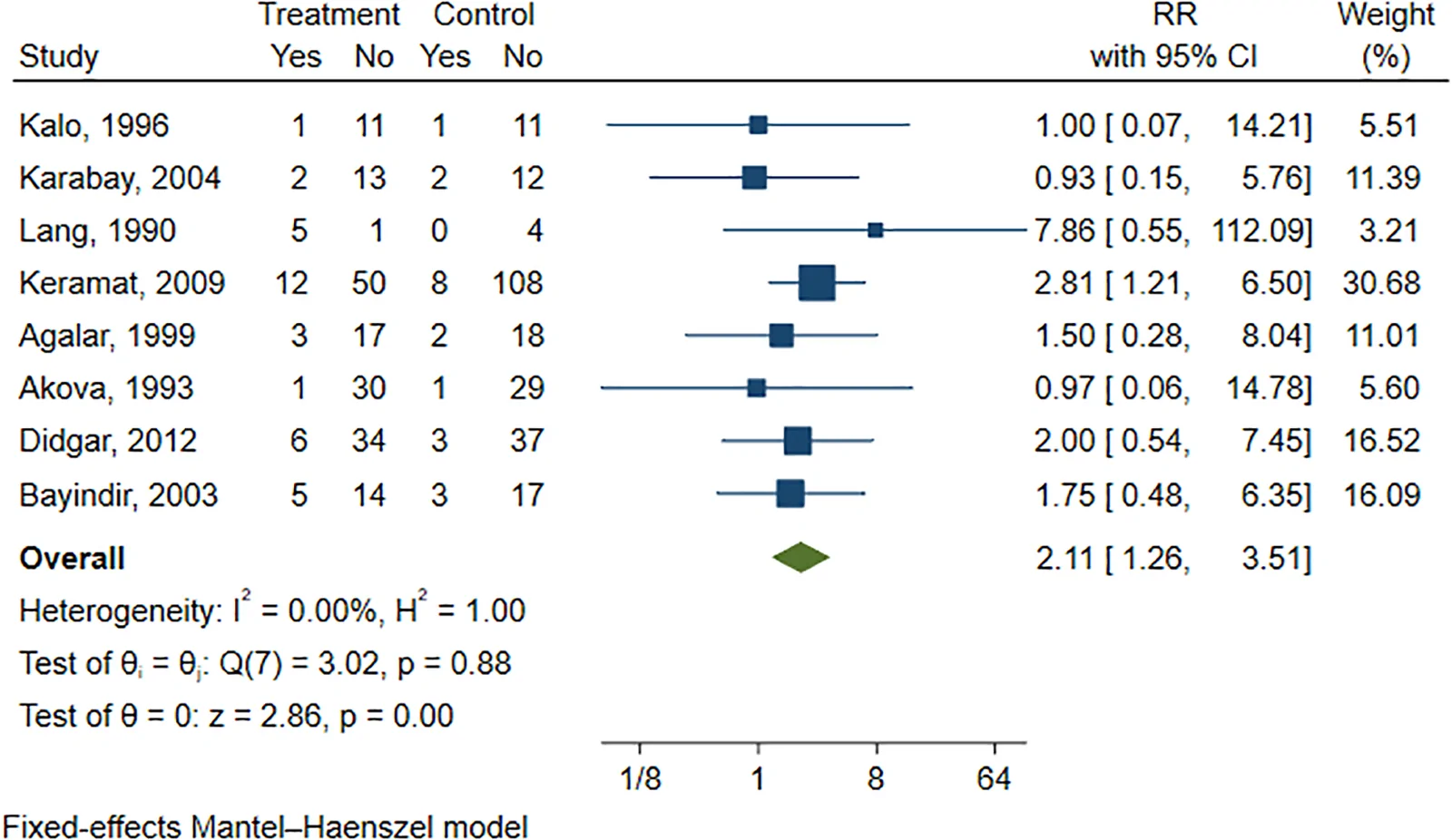
Forest plot of therapeutic failure comparing fluoroquinolone-based regimens versus standard therapy in brucellosis. A. Forest plot. B. Sensitivity analysis.

### Relapse.

Relapse outcomes were reported in seven studies. The pooled analysis showed that fluoroquinolone-based regimens were associated with a higher risk of relapse compared with standard therapy; however, this difference was not statistically significant (RR 1.88, 95% CI: 0.94–3.74, p = 0.07) (Fig 3). The fixed-effect Mantel–Haenszel model was used. No statistically significant between-study heterogeneity was observed (Q = 4.84, df = 6, p = 0.56; I² = 0%), indicating consistency across studies. Statistical significance was achieved only after omitting Keramat (2009) (RR 2.67, 95% CI: 1.14–6.26, p = 0.024), whereas the association remained statistically non-significant in all other leave-one-out repetitions. These findings indicate that while the overall point estimate favored a higher risk of relapse with fluoroquinolone-based regimens, the overall association was not statistically significant and was sensitive to the inclusion of Keramat (2009).

**Fig 3:**
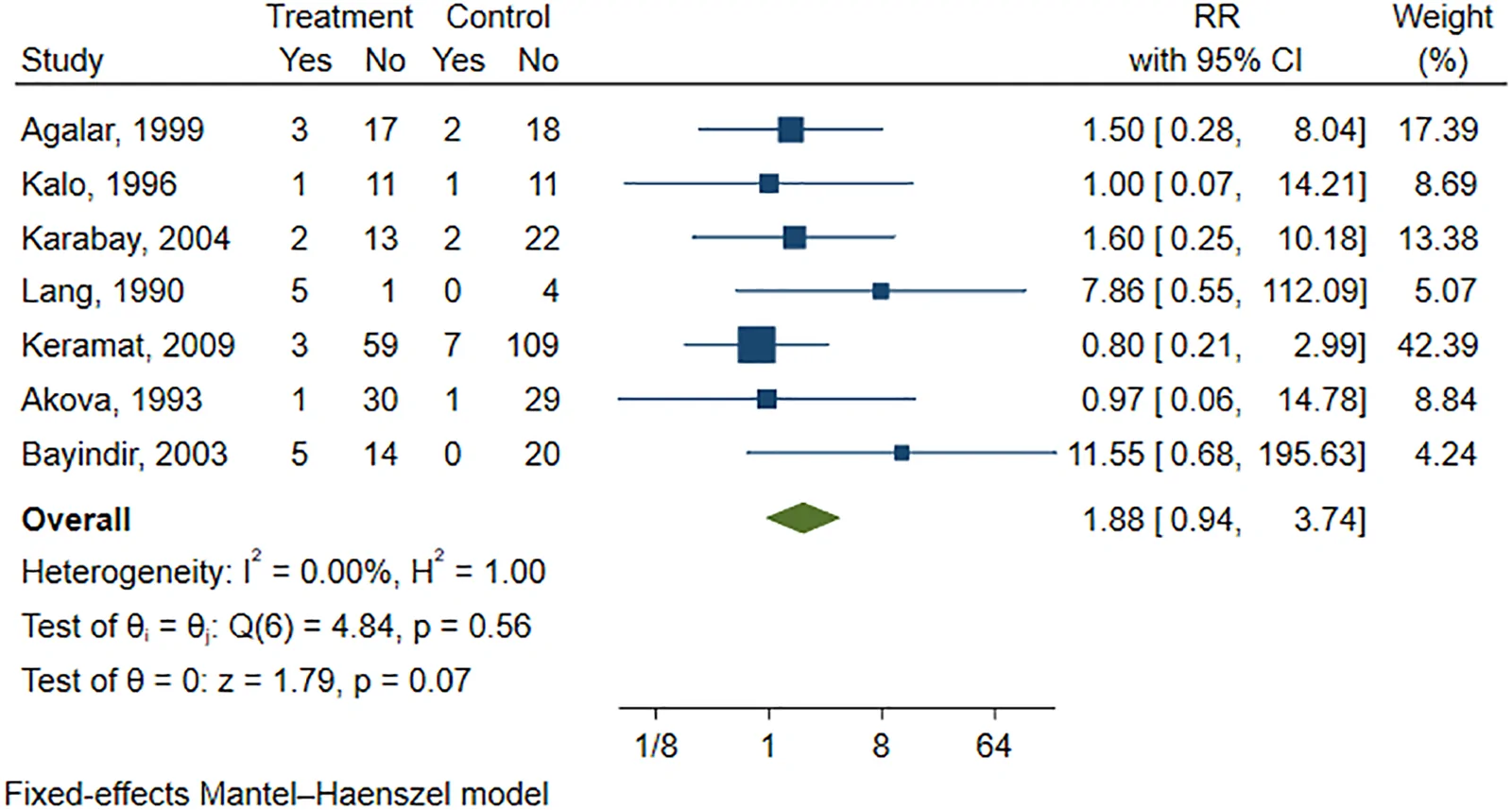
Forest plot of relapse comparing fluoroquinolone-based regimens versus standard therapy in brucellosis. A. Forest plot. B. Sensitivity analysis.

### Adverse events.

Seven studies reported data on adverse events. The pooled analysis showed that fluoroquinolone-based regimens were associated with a lower risk of adverse events compared with standard therapy; however, this difference was not statistically significant (RR: 0.62, 95% CI: 0.29–1.33; p = 0.22). (Fig 4). Moderate between-study heterogeneity was observed (Cochran’s Q = 12.68, df = 6, p = 0.05; I^2^ = 52.70), indicating variability among the study estimates. The random-effects DerSimonian–Laird model was used. In the leave-one-out sensitivity analysis, the pooled risk ratios ranged from 0.53 to 0.77, and all corresponding 95% confidence intervals included 1.00. The results therefore remained statistically non-significant after omission of each individual study, although the direction of the pooled association remained consistent, with all pooled RRs below 1.00 (Fig 4).

**Fig 4:**
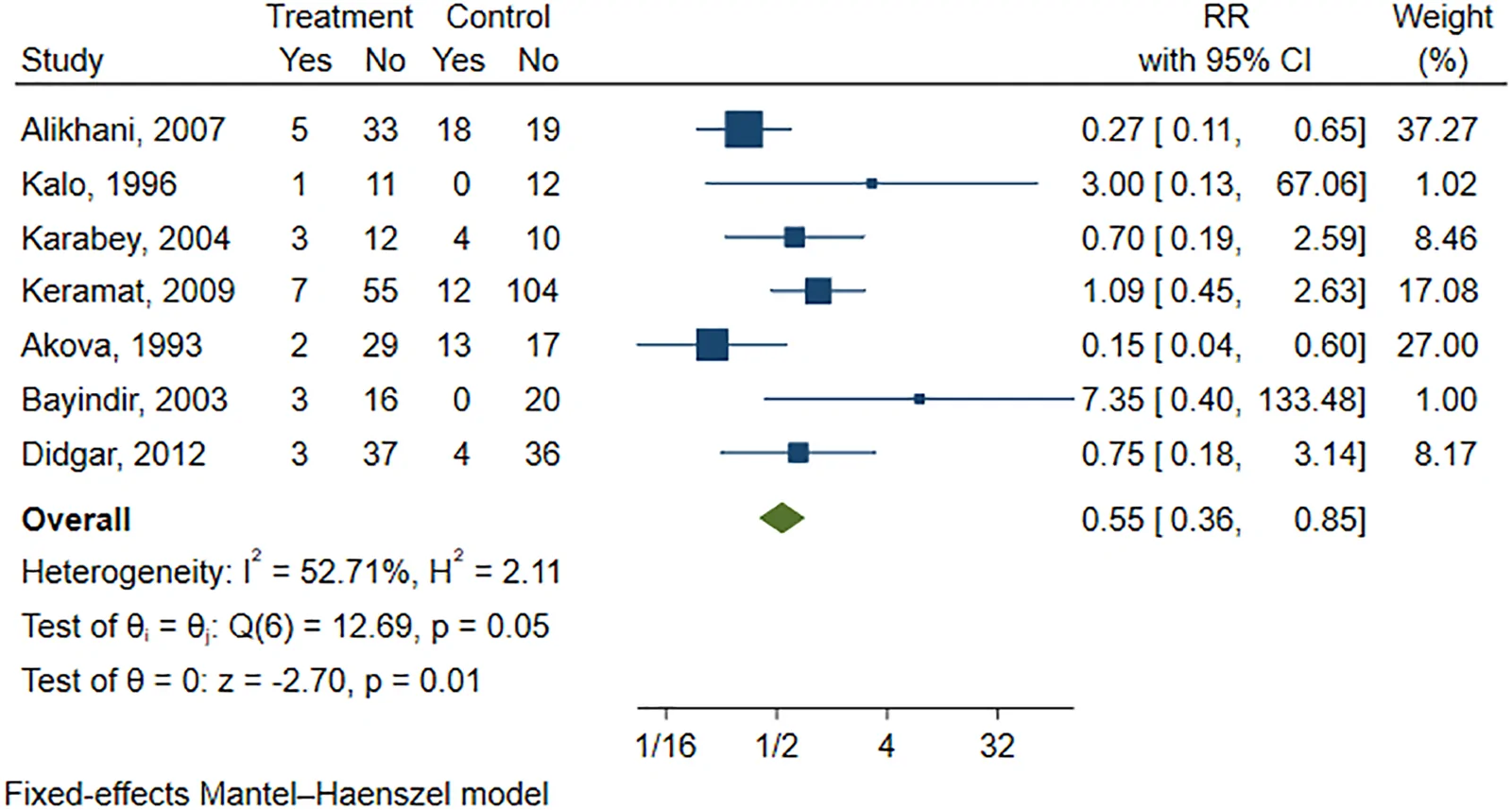
Forest plot of adverse event comparing fluoroquinolone-based regimens versus standard therapy in brucellosis. A. Forest plot. B. Sensitivity analysis.

### Time to defervescence.

Time to defervescence was evaluated in four studies. Patients receiving fluoroquinolone-based regimens had a longer time to fever reduction than those receiving conventional therapy. The pooled mean difference was 1.20 days (95% CI: 0.66–1.74; z = 4.36, p < 0.001) (Fig 5). No meaningful between-study heterogeneity was observed (Cochran’s Q = 0.12, df = 3, p = 0.99; I^2^ = 0%), indicating a high degree of consistency across the included studies. The analysis was conducted using a fixed-effects inverse-variance model. However, the study-specific confidence intervals for Lang (1990) and Akova (1993) included the null value, whereas the overall pooled estimate remained statistically significant.

**Fig 5:**
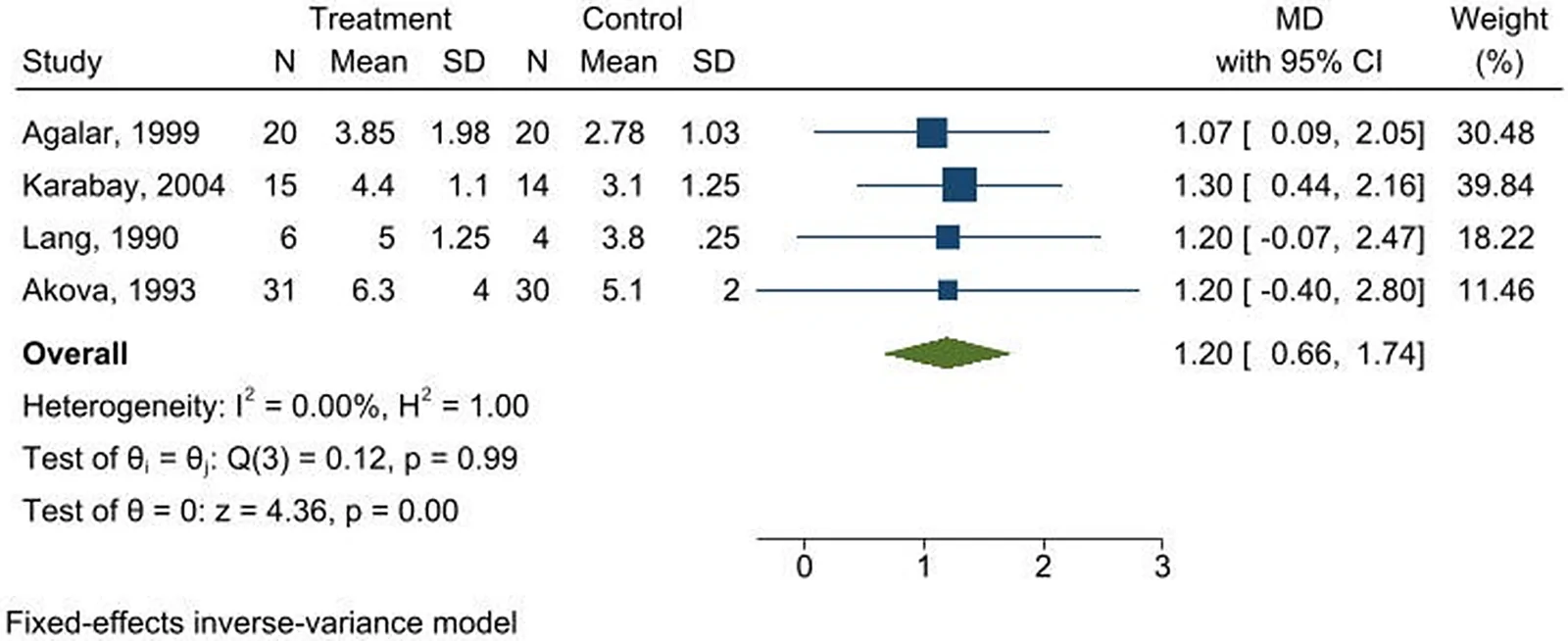
Forest plot of time to defervescence comparing fluoroquinolone-based regimens versus standard therapy in brucellosis.

## Subgroup analyses

### Therapeutic failure

Subgroup analyses indicated that fluoroquinolone-based regimens were associated with an increased risk of therapeutic failure across the evaluated subgroups. By treatment duration, the risk was higher in the 6–8-week subgroup (RR = 2.53, 95% CI: 0.62–10.22) and the > 8-week subgroup (RR = 2.52, 95% CI: 1.25–5.12) than in the ≤ 6-week subgroup (RR = 1.44, 95% CI: 0.59–3.48). However, statistical significance was observed only in the > 8-week subgroup. When stratified by intervention type, ciprofloxacin-based regimens showed the highest estimated risk of therapeutic failure (RR = 7.86, 95% CI: 0.55–112.09), although the association was not statistically significant. Ciprofloxacin–doxycycline regimens (RR = 1.75, 95% CI:0.54–5.62), ciprofloxacin–rifampin regimens (RR = 2.46, 95% CI: 1.17–5.19), and ofloxacin–rifampin regimens (RR = 1.34, 95% CI: 0.51–3.53) also showed increased risks; however, statistical significance was observed only for the ciprofloxacin–rifampin subgroup. By population type, patients with brucellar spondylitis (RR = 1.75, 95% CI: 0.48–6.35) and hospitalized patients (RR = 1.21, 95% CI: 0.36–4.13) had higher, but statistically non-significant, risks of therapeutic failure. Uncomplicated brucellosis was associated with a statistically significant increase in therapeutic failure (RR = 2.52, 95% CI: 1.34–4.75) (Table 2).

**Table 2:** Subgroup meta-analysis of fluoroquinolone-based regimens versus standard therapy in brucellosis across therapeutic failure, relapse, adverse events, and time to defervescence.

Outcomes	Groups	Subgroups	RR (95% CIs)	Heterogeneity assessment between studies		Heterogeneity assessment between subgroup	
				I square	P value	Q	P value
Therapeutic Failure	Treatment duration	<6 weeks	1.44[0.59-3.48]	0.00%	0.86	1.03	0.60
		6 – 8 weeks	2.53[0.62-10.22]	0.00%	0.44		
		>8 weeks	2.52[1.25-5.12]	0.00%	0.67		
	Type of interventions	Ciprofloxacin	7.86[0.55-112.09]	0.00%	–	2.02	0.57
		Ciprofloxacin-Doxycycline	1.75[0.54-5.62]	0.00%	0.65		
		Ciprofloxacin-Rifampin	2.46[1.17-5.19]	0.00%	0.44		
		Ofloxacin-Rifampin	1.34[0.51-3.53]	0.00%	0.83		
	Population	Brucellar Spondylitis	1.75[0.48-6.35]	0.00%	–	1.17	0.56
		Hospitalized	1.21[0.36-4.13]	0.00%	0.71		
		Uncomplicated	2.52[1.34-4.75]	0.00%	0.77		
Relapse	Treatment duration	<6 weeks	1.54[0.45-5.35]	0.00%	0.96	3.12	0.21
		6 – 8 weeks	3.95[1.18-13.28]	0.00%	0.41		
		>8 weeks	0.80[0.21-2.99]	0.00%	–		
	Type of interventions	Ciprofloxacin	7.86[0.55-112.09]	0.00%	–	3.23	0.36
		Ciprofloxacin-Doxycycline	1.00[0.07-14.21]	0.00%	–		
		Ciprofloxacin-Rifampin	1.00[0.36-2.80]	0.00%	0.56		
		Ofloxacin-Rifampin	2.98[0.86-10.30]	0.00%	0.37		
	Population	Brucellar Spondylitis	11.55[0.68-195.63]	–		1.95	0.38
		Hospitalized	1.54[0.45-5.35]	0.00%	0.96		
		Uncomplicated	1.40[0.56-3.52]	0.00%	0.49		
Adverse event	Treatment duration	<6 weeks	0.70[0.19-2.59]	0.00%	–	0.22	0.89
		6 – 8 weeks	0.14[0.08-16.9]	72.92%	0.02		
		>8 weeks	0.59[0.23-1.51]	59.87%	0.08		
	Type of interventions	Ofloxacin-Streptomycin-Rifampin	0.27[0.11-0.65]	0.00%	–	6.39	0.0.4
		Ciprofloxacin-Doxycycline	0.96[0.26-3.51]	0.00%	0.43		
		Ciprofloxacin-Rifampin	1.09[0.45-2.63]	0.00%	–		
		Ofloxacin-Rifampin	0.64[0.11-3.79]	68.72%	0.04		
	Population	Brucellar Spondylitis	7.35[0.40-133.48]	0.00%	–	3.03	0.22
		Hospitalized	0.70[0.19-2.59]	0.00%	–		
		Uncomplicated	0.50[0.21-1.21]	57.21%	0.05		
Outcomes	Groups	Subgroups	WMD (95% CIs)	Heterogeneity assessment between studies	Heterogeneity assessment between subgroup	Outcomes	Groups
Time to Defervescence	Treatment duration	<6 weeks	1.22 (0.56, 1.84)	0.00%	0.73	0.00	1.00
		6 – 8 weeks	1.05 (0.21, 2.19)	0.00%	1.00		
	Type of interventions	Ciprofloxacin	1.20 (-0.07, 2.47)	0.00%	—	0.11	0.95
		Ciprofloxacin-Rifampin	1.07 (0.09, 2.05)	0.00%	—		
		Ofloxacin-Rifampin	1.28 (0.52, 2.03)	0.00%	0.91		
	Population	Hospitalized	1.18 (0.56, 1.84)	0.00%	0.73	0.00	1.00
		Uncomplicated	1.05 (0.21, 2.19)	0.00%	1.00		

### Relapse

Subgroup analysis of relapse outcomes showed that the association was not statistically significant in most subgroups. By treatment duration, the risk of relapse was not significantly increased in the < 6-week subgroup (RR = 1.54, 95% CI: 0.45–5.35) or the > 8-week subgroup (RR = 0.80, 95% CI: 0.21–2.99). However, the 6–8-week subgroup showed a statistically significant increase in relapse risk (RR = 3.95, 95% CI: 1.18–13.28). Across intervention types, none of the fluoroquinolone-based regimens showed a statistically significant difference compared with standard therapy: ciprofloxacin (RR = 7.86, 95% CI: 0.55–112.09), ciprofloxacin–doxycycline (RR = 1.00, 95% CI: 0.07–14.21), ciprofloxacin–rifampin (RR = 1.00, 95% CI: 0.36–2.80), and ofloxacin–rifampin (RR = 2.98, 95% CI: 0.86–10.30). By population type, hospitalized patients did not show a statistically significant increase in relapse risk (RR = 1.54, 95% CI: 0.45–5.35), while patients with brucellar spondylitis had a higher but statistically non-significant risk (RR = 11.55, 95% CI: 0.68–195.63). Uncomplicated brucellosis also did not show a statistically significant association (RR = 1.40, 95% CI: 0.56–3.52) (Table 2).

### Adverse events

Although individual studies reported varying safety outcomes, the overall evidence did not support a consistent increase in adverse events among patients treated with fluoroquinolone-based regimens compared with standard therapy. By treatment duration, the risk of adverse events was not statistically significant in the < 6-week subgroup (RR = 0.70, 95% CI: 0.19–2.59), the 6–8-week subgroup (RR = 1.14, 95% CI: 0.08–16.90), or the > 8-week subgroup (RR = 0.59, 95% CI: 0.23–1.51). Regarding intervention type, ofloxacin–streptomycin–rifampin regimens (RR = 0.27, 95% CI: 0.11–0.65) were associated with a statistically significant reduction in adverse events, whereas ciprofloxacin–doxycycline (RR = 0.96, 95% CI: 0.26–3.51), ciprofloxacin–rifampin (RR = 1.09, 95% CI: 0.45–2.63), and ofloxacin–rifampin regimens (RR = 0.64, 95% CI: 0.11–3.79) did not show statistically significant differences. By population type, brucellar spondylitis was not associated with a statistically significant difference in adverse events (RR = 7.35, 95% CI: 0.40–133.48), and hospitalized patients also showed no significant difference (RR = 0.70, 95% CI: 0.19–2.59). Uncomplicated brucellosis was not associated with a statistically significant reduction in adverse events (RR = 0.50, 95% CI: 0.21–1.21) (Table 2).

### Time to defervescence

For time to defervescence, subgroup analyses showed no statistically significant differences across treatment-duration categories. The mean difference was 1.22 days (95% CI: 0.56–1.84) in the < 6-week subgroup and 1.05 days (95% CI: 0.21–2.19) in the 6–8-week subgroup. Across intervention types, ciprofloxacin was not associated with a statistically significant difference (MD = 1.20, 95% CI: −0.07–2.47), whereas ciprofloxacin–rifampin (MD = 1.07, 95% CI: 0.09–2.05) and ofloxacin–rifampin (MD = 1.28, 95% CI: 0.52–2.03) were associated with significantly longer time to defervescence. By population type, the mean difference was 1.18 days (95% CI: 0.56–1.84) among hospitalized patients and 1.05 days (95% CI: 0.21–2.19) among patients with uncomplicated brucellosis. No substantial heterogeneity was observed within the subgroups (I^2^ = 0%Between-subgroup differences were not statistically significant for treatment duration (Q = 0.00, p = 1.00), intervention type (Q = 0.11, p = 0.95), or population type (Q = 0.00, p = 1.00) (Table 2).

#### Publication bias.

Visual examination of the funnel plots reveals no clear asymmetries. For all the results, neither the Begg’s rank correlation test nor the Egger’s regression test for publication bias were statistically significant. For therapeutic failure, Begg’s test was not statistically significant (Kendall’s tau = -0.3571, p = 0.26), and Egger’s test also showed no significant asymmetry (intercept = -0.631, 95% CI: -2.06 to 0.80; t = -1.08, p = 0.32). Similarly, no statistically significant publication bias was detected for relapse (Begg’s tau = 0.42, p = 0.22; Egger’s intercept = 1.81, 95% CI: -0.66 to 4.28; t = 1.88, p = 0.11) or adverse events (Begg’s tau = 0.14, p = 0.76; Egger’s intercept = 1.86, 95% CI: -2.15 to 5.13; t = 1.54, p = 0.12). For time to defervescence, Kendall’s tau was 0 (p-value not available), while Egger’s test showed no significant publication bias (intercept = -0.16, 95% CI: -2.45 to 2.10; t = -0.31, p = 0.78) (Table 3).

**Table 3:** Assessment of publication bias in the impact of fluoroquinolone on brucellosis.

	Corrected Effect size				Begg’s rank correction test		Egger’s linear regression test			
	RR	95%CI	Weighted Mean Difference	95%CI	Kendal Tau	P	Intercept	95%CI	T value	P
Therapeutic Failure	2.11	1.26-3.51	–	–	-0.3571	0.26	-0.631	-2.06, 0.80	-1.08	0.32
Relapse	1.88	0.94-3.74	–	–	0.42	0.22	1.81	-0.66, 4.28	1.88	0.11
Adverse event	0.62	0.29-1.33	–	–	0.14	0.76	1.86	-2.15, 5.13	1.54	0.12
Time to Defervescence	–	–	1.20	0.66-1.74	0	–	-0.16	-2.45, 2.1	-0.31	0.78

### GRADE assessment

The degree of evidentiary certainty changed depending on the outcome when using the GRADE method. The estimate was supported by consistent results across included trials and no indication of statistical heterogeneity, however the certainty of evidence for therapeutic failure was rated as moderate, reduced due to concerns about risk of bias and potential publication bias.

Due to issues with imprecision, specifically the large confidence intervals and small number of outcome events, the certainty of the evidence for relapse was rated as low. Despite the low level of heterogeneity and the general consistency of effect estimates across the included trials, this grade remained in place. The certainty of evidence for adverse events was rated as moderate, with downgrading primarily because to imprecision and differences in sample sizes between studies. Despite considerable variation, the direction of effect stayed constant, and the overall risk of bias was deemed minimal. Because of methodological issues in the included trials, the certainty of the evidence for time to defervescence was deemed to be moderate. Despite this, the pooled estimate was solid, with comparatively accurate confidence ranges and minimal between-study variability. The certainty of evidence was moderate for therapeutic failure, adverse events, and time to defervescence, and low for relapse. These results imply that, in comparison to standard therapy, fluoroquinolone-based regimens may be linked to a higher risk of therapeutic failure and a delayed clinical response; however, the evidence supporting variations in relapse and adverse events is still sparse and should be interpreted cautiously (S5 Table).

## Discussion

We provide a thorough quantitative synthesis of nine clinical trials with 602 patients using systematic review and meta-analytic techniques, as well as a comprehensive evaluation of fluoroquinolone-containing treatment regimens in comparison to conventional treatment regimens for human brucellosis. When compared to standard therapy, fluoroquinolone-containing regimens were linked to a significantly higher risk of therapeutic failure (RR = 2.11, 95% CI: 1.26–3.51) and a significantly longer time to defervescence (MD = 1.20 days, 95% CI: 0.66–1.74). For these results, there was not statistically significant between-study heterogeneity (I^2^ = 0%).

The pooled effect for relapse was not statistically significant (RR = 1.88, 95% CI: 0.94–3.74; p = 0.07), but the risk of relapse was significantly increased in the 6–8-week treatment-duration subgroup (RR = 3.95, 95% CI: 1.18–13.28). However, fluoroquinolone-containing regimens were not associated with a statistically significant difference in adverse events under the random-effects model (RR = 0.62, 95% CI: 0.29–1.33). Although the point estimate suggested a possible reduction in adverse events, the confidence interval included the null effect. The results of this study indicate that any potential advantage of fluoroquinolone-containing regimens in terms of tolerability should be interpreted cautiously, particularly in view of the higher risk of therapeutic failure and delayed resolution of fever. The point estimate for adverse events may partly reflect differences in the adverse-event profiles of standard regimens, especially doxycycline- and rifampicin-containing combinations; however, this interpretation should be made with caution.

Our findings are broadly consistent with recent evidence indicating that fluoroquinolone-containing regimens should not routinely replace established doxycycline-based combinations for the treatment of human brucellosis. A 2024 systematic review and network meta-analysis of 43 randomized controlled trials ranked doxycycline combined with an aminoglycoside (particularly gentamicin or streptomycin) among the most effective strategies, while quinolone-containing regimens demonstrated comparatively lower efficacy in several comparisons, albeit with limited certainty of evidence [21].

These results align with current treatment recommendations that continue to favor doxycycline plus rifampicin or an aminoglycoside as first-line options for uncomplicated brucellosis, reserving fluoroquinolones primarily as alternative agents [22–24].

In this context, our observation of a higher risk of therapeutic failure and delayed defervescence with fluoroquinolone-containing regimens without a statistically significant difference in adverse events—further supports the continued use of established doxycycline-based combinations as first-line therapy.

The intracellular localization of *Brucella* species within acidic phagolysosomal compartments represents a potential obstacle to successful treatment, as fluoroquinolone activity may be attenuated under such conditions [25–28].

Recent in vitro and pharmacokinetic studies published in 2024 and 2025 also suggest that, despite their favorable tissue penetration, fluoroquinolones may show reduced activity under acidic conditions, although their activity may differ among individual agents [2].

The observed findings are broadly supported by previous evidence, which similarly demonstrated that ciprofloxacin undergoes pH-structured ionization in acidic environments, which may reduce intracellular accumulation, whereas ofloxacin maintains a remarkably better intracellular equilibrium [29–31].

In addition to the pH-related limits, Brucella has several potential mechanisms of fluoroquinolone resistance. Mutations in the quinolone resistance locus recognition (QRDR), particularly in the gyrA and gyrB genes, reduce fluoroquinolone binding affinity [32].

Fluoroquinolone exposure may also promote the selection of resistance-associated mutations in quinolone resistance hotspots, especially in gyrA, including Ala87Val and Ser90Phe substitutions. Moreover, genomic investigations have highlighted the upregulation of efflux-related genes, including *mprF* and several representatives of the *bep* family. This mechanism may facilitate decreased intracellular exposure to fluoroquinolones, potentially attenuating their bactericidal activity against *Brucella* species [31,33]. These mechanisms may partly explain the reduced clinical effectiveness observed with fluoroquinolone-containing regimens.

The limited translation of in vitro susceptibility findings in which numerous fluoroquinolones show low MICs, into in vivo clinical efficacy highlights the complexity of the host–pathogen–drug interaction [4,34].

Additionally, *Brucella*’s capacity to establish long-term infection in non-phagocytic cells—where it replicates within endoplasmic reticulum-derived compartments (as shown in Vero and other epithelial cell models) together with the existence of antibiotic-tolerant “persister” subpopulations that survive antimicrobial pressure, may help explain the higher rates of therapeutic failure seen with fluoroquinolone-containing regimens, particularly those limited to 6–8 weeks [35,36].

The above-mentioned mechanisms are supported by the recent multicenter observational studies performed in endemic settings that revealed a reduced likelihood of relapse in both uncomplicated and complicated osteoarticular brucellosis when fluoroquinolones were not included in the treatment regimen [24,37].

However, well-established doxycycline-based regimens combined with rifampicin or an aminoglycoside have demonstrated favorable clinical outcomes, potentially related to their intracellular activity and synergistic effects. A 2025 review using ISCHEM concluded that recent evidence indicates that fluoroquinolone-containing regimens have not been established as equivalent to standard doxycycline-based therapies for intracellular sterilization [1].

Our results agree with the existing clinical practice guidelines, which still recommend combined treatments based on doxycycline, in conjunction either with aminoglycosides (Streptomycin or Gentamicin), or rifampicin, as the first choice for the treatment of uncomplicated human brucellosis. Network meta-analyses conducted in recent years found the combination of doxycycline with gentamicin or streptomycin to be the most effective regimen, whereas combinations with fluoroquinolones were found to have relatively low effectiveness. There is no recommendation for using fluoroquinolone-containing combinations as first-choice treatment in the current guidelines and expert consensus. Thus, the increased risk of treatment failure and relapse found in our meta-analysis further confirms the clinical utility of using proven combinations of doxycycline. Fluoroquinolones may be used as alternative drugs only in those cases when standard drugs are contraindicated or poorly tolerated [21,24].

The current meta-analysis has several methodological strengths that should be acknowledged. The use of randomized trials, the very low heterogeneity for the most critical outcomes, and the assessment of sensitivity to the choice of statistical model provide greater robustness to the meta-analysis. However, several limitations should be acknowledged. These include the very small sample sizes in some of the included studies, the fact that the vast majority of the studies were conducted between 1990 and 2014, and the low certainty of the evidence coupled with potentially imprecise effect estimates all of which may limit the validity and generalizability of our findings. Furthermore, most of the available data originate from endemic countries, particularly Iran and Turkey.

Considering all these findings, there is no evidence of superiority of fluoroquinolone regimens compared with standard doxycycline regimens. Although the point estimates for adverse events favored fluoroquinolone-containing regimens, this difference was not statistically significant under the random-effects model. This possible advantage in terms of tolerability should therefore be interpreted cautiously, particularly considering the significantly higher risks of therapy failure and prolonged defervescence period. Overall, the increase in relapses did not reach statistical significance; however, the increase in the 6–8-week duration of therapy subgroup was significantly higher.

## Conclusion

Current synthesis of evidence suggests that fluoroquinolone-based regimens were associated with a higher risk of therapeutic failure, as well as a longer time to defervescence, than standard doxycycline-based regimens for the treatment of acute brucellosis. Under the random-effects model, fluoroquinolone-containing regimens did not show a statistically significant difference in adverse events compared with standard regimens (RR = 0.63, 95% CI: 0.29–1.33). Although the point estimate favored fluoroquinolone-containing regimens, the confidence interval included the null effect. The increase in overall relapse was not statistically significant, although a significantly higher risk was noted in the subgroup with a treatment duration of 6–8 weeks. Regimens containing fluoroquinolones should not be routinely preferred to be established doxycycline-based combinations for first-line therapy, due to moderate certainty of evidence for therapeutic failure and time to defervescence, and low certainty of evidence for relapse.

### Limitations

Although this study provides a comprehensive synthesis of the available evidence, several limitations merit consideration. Chief among these is the fact that all included randomized controlled trials were published between 1990 and 2014, emphasizing limited availability of contemporary randomized data in patients with brucellosis. This limitation may indicate that changes in epidemiology, antimicrobial susceptibility patterns, and clinical management over time have not been adequately captured. Furthermore, most of the available data originated from endemic countries, particularly Iran and Turkey, which may limit the generalizability of the findings to non-endemic settings. Thirdly, Thirdly, clinical heterogeneity in the treatment regimens, drug combinations, and treatment durations existed among the included trialseven though statistical heterogeneity was low for the outcomes of interest. Fourthly, the operational definitions of therapeutic failure and relapse differed to some extent between the included trials, and follow-up durations were inconsistent.

The possibility of publication bias cannot be completely excluded. Begg’s and Egger’s tests did not suggest statistically significant publication bias; however, these tests have limited power when the number of included studies is small**.** Thus, the possibility of publication bias cannot be completely excluded.

In addition, the extremely wide confidence intervals observed for some outcomes indicate substantial statistical imprecision. This limitation, mainly due to the small sample sizes and low event rates in some of the included trials, contributed to downgrading the certainty of evidence to “low” by GRADE for the relevant outcomes**.**

### Declaration of generative AI and AI-assisted technologies in the writing process

During the preparation of this manuscript, the authors used Gemini 3.1 Pro and ChatGPT Plus solely to improve the grammar, language, and readability of the text. After using these tools, the authors reviewed and edited the content as needed and take full responsibility for the content of the publication.

## Supporting information

S1 Table Search strategies used in the systematic review. Detailed search strategies used to identify relevant studies across the databases searched.
(DOCX)

S2 Table Study characteristics of all included studies. Summary of the main characteristics of all studies included in the systematic review and meta-analysis.
(DOCX)

S3 Table Risk-of-bias assessment results. Results of the risk-of-bias assessments for the included studies.
(DOCX)

S4 Table List of studies excluded after full-text screening. List of studies excluded following full-text assessment, with reasons for exclusion.
(DOCX)

S5 Table GRADE assessment results. Results of the assessment of the certainty of evidence using the GRADE approach.
(DOCX)

S1 Checklist PRISMA 2020 checklist. Completed PRISMA 2020 checklist for the systematic review and meta-analysis. Based on: Page MJ, McKenzie JE, Bossuyt PM, Boutron I, Hoffmann TC, Mulrow CD, et al. The PRISMA 2020 statement: an updated guideline for reporting systematic reviews. *BMJ*. 2021;372. https://doi.org/10.1136/bmj.n71. The PRISMA 2020 checklist is available under the Creative Commons Attribution 4.0 International (CC BY 4.0) license: https://creativecommons.org/licenses/by/4.0/.
(DOCX)
