# Supplementary material for: Efficacy and safety of fluoroquinolones in the treatment of human brucellosis: A systematic review and meta-analysis
Source: PLoS Negl Trop Dis. 2026 Oct 23;20(10):e0013822. doi: 10.1371/journal.pntd.0013822 (PMC13722316; doi:10.1371/journal.pntd.0013822)
Supplement: Supplementary file 1 [file pntd.0013822.s008.pdf]

Comment on the paper

S.No.	Points considered for the review	My comments/feedback (summary)
1.	What are the main claims of the paper and how significant are they for the discipline?	The topic is interesting and significant for the discipline
2.	Are these claims novel? If not, which published articles weaken the claims of originality of this one?	It is not as such Novel, there is recent work (in 2024) and it did not justify why it is relevant to make this review. Most old studies referred are very old
3.	Are the claims properly placed in the context of the previous literature? Have the authors treated the literature fairly?	Effort has been made, but the authors should have justified why and how the focus on those drugs is still important than the recent and technological based experiments (e.g.<br><br>1. Phage Therapy: A Targeted Solution for Brucellosis. Lashtoo Aghaee B, Alikhani MY, Kazemi S, Ahmadyousefi M, van Leeuwen WB, Nasaj M.Vector Borne Zoonotic Dis. 2025 Dec;25(12):713-721. doi: 10.1177/15303667251367519. Epub 2025 Aug 11.PMID: 40788720 Review.<br>2. CRISPR technology for diagnosis and treatment of human brucellosis. Lau CH, Li X, Liang QL, Guo R, Ren X, Xu Z, Zhu Y, Lai Y, Liu G, Huang Y, Wu W, Zhu H, Chen J, Zhang X.J Microbiol Methods. 2026 Jan;240:107339. doi: 10.1016/j.mimet.2025.107339. Epub 2025 Nov 20.<br>3. Complementary Use of Probiotics and Their Antimicrobial Proteins in Brucellosis: A Comprehensive Review of Mechanisms and Clinical Benefits. Soozangar N, Faghfuri E, Gholizadeh P.Probiotics Antimicrob Proteins. 2025 Aug 27. doi: 10.1007/s12602-025-10731-y. Online ahead of print.PMID: 40864417 Review.
3.	Do the data and analyses fully support the claims? If not, what other evidence is required?	Yes, most of the data are old (references)
4.	Would additional work improve the paper? How much better would the paper be if this work were performed and how difficult would it be to do this work?	I don’t see the relevance of this or any additional work. Because based on enough international researches the WHO is discouraging the use of fluoroquinolones as the primary therapy for acute human brucellosis and it has been suggested that the first-line drug therapy for acute brucellosis should be doxycycline and rifampicin or streptomycin.
5.	*PLOS Neglected Tropical Diseases* encourages authors to publish detailed protocols and	No problem with review process/method

	algorithms as supporting information online. Do any particular methods used in the manuscript warrant such treatment? If a protocol is already provided, for example for a randomized controlled trial, are there any important deviations from it? If so, have the authors explained adequately why the deviations occurred?	
6.	Is this paper outstanding in its discipline? If yes, what makes it outstanding? If not, why not?	Not outstanding, for the reasons I mentioned above
7.	If the paper is considered unsuitable for publication in its present form, does the study itself show sufficient potential that the authors should be encouraged to resubmit a revised version?	I am not convinced that much, the authors relied on old references, and limited data to make comparisons between the different parameters.
8.	Are original data deposited in appropriate repositories and accession/version numbers provided for genes, proteins, mutants, diseases, etc.?	Not indicated as to where the data is deposited
9.	Are details of the methodology sufficient to allow the experiments to be reproduced?	-
10.	Is the manuscript well organized and written clearly enough to be accessible to non-specialists?	Not, the justification regarding the need of making the review is not convincing/not addressed, there are lots of fragmented ideas, and many paragraphs with just one or two sentences, with no or few references cited, follow of ideas is ruined, (particularly in the discussion part), very old references used and current researches/advancements in the search of the treatment are not touched in the paper, the styles followed in the reference writing is very different, some references are duplicated, Most tables title and numbering and some are duplicated
