# Supplementary material for: Efficacy and safety of fluoroquinolones in the treatment of human brucellosis: A systematic review and meta-analysis
Source: PLoS Negl Trop Dis. 2026 Oct 23;20(10):e0013822. doi: 10.1371/journal.pntd.0013822 (PMC13722316; doi:10.1371/journal.pntd.0013822)
Supplement: Supplementary file 2 [file pntd.0013822.s004.docx]

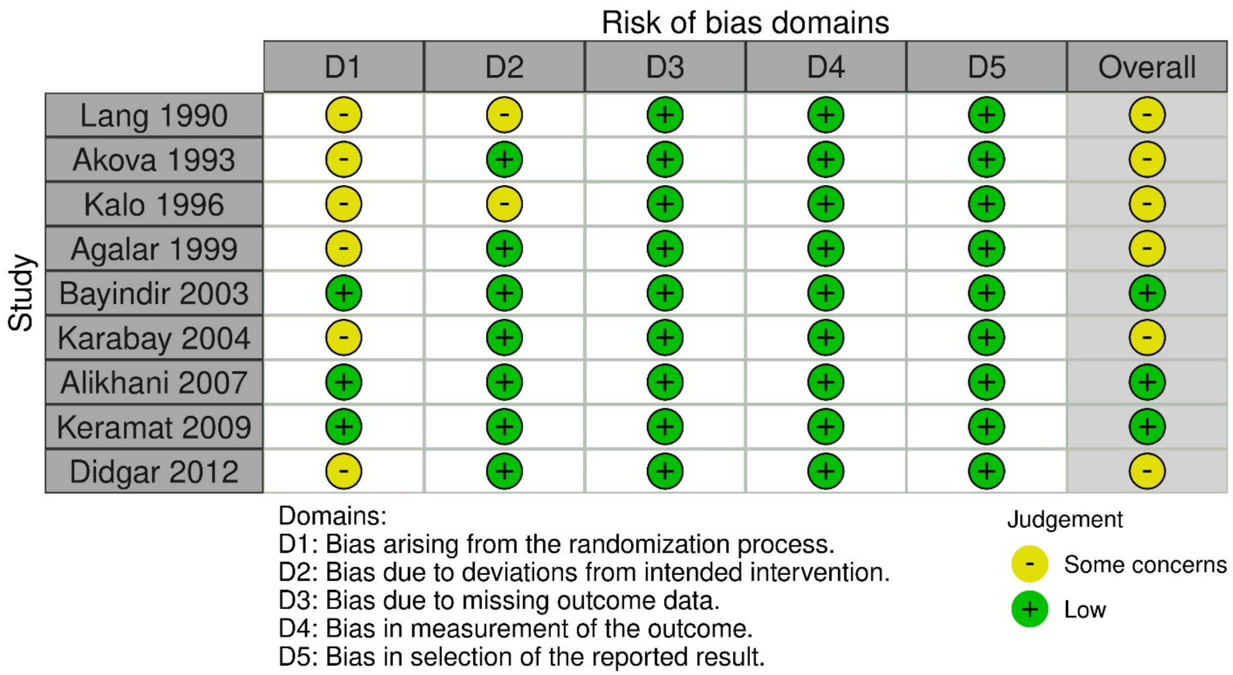
Risk of bias domains
D1
D2
D3
D4
D5
Overall
Study
Lang 1990 - - + + + -
Akova 1993 - + + + + -
Kalo 1996 - - + + + -
Agalar 1999 - + + + + -
Bayindir 2003 + + + + + +
Karabay 2004 - + + + + -
Alikhani 2007 + + + + + +
Keramat 2009 + + + + + +
Didgar 2012 - + + + + -
Domains:
D1: Bias arising from the randomization process.
D2: Bias due to deviations from intended intervention.
D3: Bias due to missing outcome data.
D4: Bias in measurement of the outcome.
D5: Bias in selection of the reported result.
Judgement
- Some concerns
+ Low

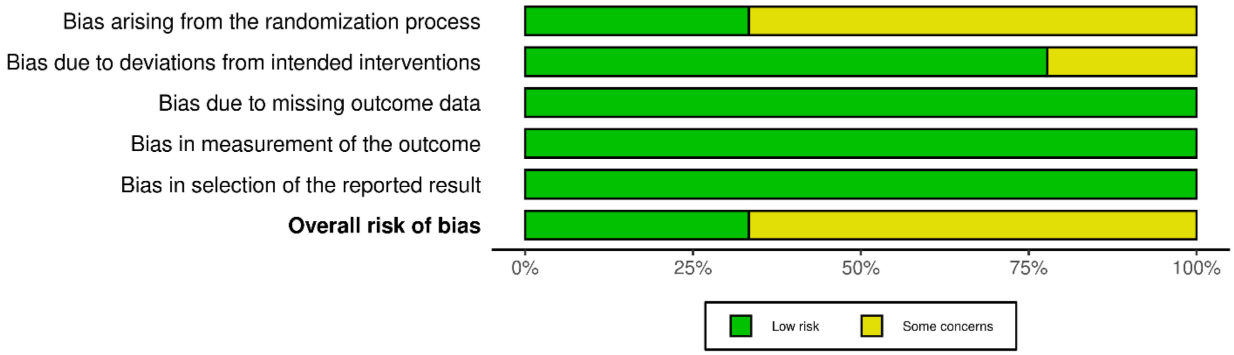
Bias arising from the randomization process
Bias due to deviations from intended interventions
Bias due to missing outcome data
Bias in measurement of the outcome
Bias in selection of the reported result
Overall risk of bias
0%
25%
50%
75%
100%
Low risk
Some concerns
