# Supplementary material for: Efficacy and safety of fluoroquinolones in the treatment of human brucellosis: A systematic review and meta-analysis
Source: PLoS Negl Trop Dis. 2026 Oct 23;20(10):e0013822. doi: 10.1371/journal.pntd.0013822 (PMC13722316; doi:10.1371/journal.pntd.0013822)
Supplement: Supplementary file 2 [file pntd.0013822.s001.docx]

**Efficacy and Safety of Fluoroquinolones in the Treatment of Human Brucellosis: A Systematic Review and Meta-Analysis**

| **Population** | **brucellosis patients** |
| --- | --- |
| **Intervention** | **fluroquinolone-containing regimen** |
| **Comparison** | **Standard regimen** |
| **Outcome** | **Effectiveness – relapse rate – therapeutic failure – treatment discontinuous – adverse event – time to deference** |

I (N=71,440)

"Fluoroquinolones"[MeSH Terms] OR "Ciprofloxacin"[MeSH Terms] OR "Levofloxacin"[MeSH Terms] OR "Ofloxacin"[MeSH Terms] OR "Fluoroquinolones"[MeSH Terms] OR "Ciprofloxacin"[Title/Abstract] OR "Fleroxacin"[Title/Abstract] OR "Enoxacin"[Title/Abstract] OR "Enrofloxacin"[Title/Abstract] OR "Gatifloxacin"[Title/Abstract] OR "Gemifloxacin"[Title/Abstract] OR "Moxifloxacin"[Title/Abstract] OR "Norfloxacin"[Title/Abstract] OR "Ofloxacin"[Title/Abstract] OR "Levofloxacin"[Title/Abstract] OR "Pefloxacin"[Title/Abstract]

O (N=12,351,752)

"Treatment Outcome"[MeSH Terms] OR "Therapeutics"[MeSH Terms] OR "Therapeutic*"[Title/Abstract] OR "Treatment Outcome"[Title/Abstract] OR "treatment*"[Title/Abstract] OR "therapy"[Title/Abstract] OR "efficacy"[Title/Abstract] OR "Recurrences"[Title/Abstract] OR "Relapse"[Title/Abstract] OR "Recrudescences"[Title/Abstract] OR "Clinical Efficacy"[Title/Abstract] OR "Rehabilitation Outcome"[Title/Abstract] OR "Therapies"[Title/Abstract] OR "Treatment Effectiveness"[Title/Abstract] OR "Clinical Effectiveness"[Title/Abstract]

P (N=26,664)

"Brucellosis"[MeSH Terms] OR "Brucellosis"[Title/Abstract] OR "brucell*"[Title/Abstract] OR "brucella infection"[Title/Abstract] OR "malta fever"[Title/Abstract] OR "Gibraltar Fever"[Title/Abstract] OR "undulant fever"[Title/Abstract] OR "Mediterranean Fever"[Title/Abstract] OR "malta fever"[Title/Abstract] OR "Cyprus fever"[Title/Abstract] OR "rock fever"[Title/Abstract] OR "Bang's disease"[Title/Abstract]

Study Design (N=2,806,979)

"clinical trial"[Title/Abstract] OR "single blinded method"[Title/Abstract] OR "double blinded method"[Title/Abstract] OR "cross over study"[Title/Abstract] OR "cross over stud*"[Title/Abstract] OR "random allocation"[Title/Abstract] OR "clinical trials as topic"[Title/Abstract] OR "comparative study"[Title/Abstract] OR "clinical trial*"[Title/Abstract] OR "controlled trial"[Title/Abstract] OR "Intervention"[Title/Abstract] OR "Randomized"[Title/Abstract] OR "Randomised"[Title/Abstract] OR "RCT"[Title/Abstract] OR "single blind"[Title/Abstract] OR "double blind"[Title/Abstract] OR "Parallel"[Title/Abstract] OR "Assignment"[Title/Abstract] OR "Cross-over"[Title/Abstract] OR "Randomly"[Title/Abstract]

Not animal/case report (N=1,147,091)

("rats"[Title/Abstract] OR "mice"[Title/Abstract] OR "swine"[Title/Abstract] OR "mouse"[Title/Abstract] OR "mus"[Title/Abstract] OR "rat"[Title/Abstract] OR "in-vitro"[Title/Abstract] OR "in-vitro"[Title/Abstract] OR "pig"[Title/Abstract] OR "rabbit"[Title/Abstract] OR "rooster"[Title/Abstract] OR "cell"[Title/Abstract] OR "cells"[Title/Abstract] OR "cow"[Title/Abstract] OR "cows"[Title/Abstract] OR "suide"[Title/Abstract] OR "warthogs"[Title/Abstract] OR "broilers"[Title/Abstract] OR "roiler"[Title/Abstract] OR "in-vivo"[Title/Abstract] OR "in-vivo"[Title/Abstract] OR "rats"[Title/Abstract] OR "pigs"[Title/Abstract] OR "rats*"[Title/Abstract] OR "protein"[Title/Abstract] OR "goats"[Title/Abstract] OR "chicken"[Title/Abstract] OR "shrimp"[Title/Abstract] OR "sheep"[Title/Abstract] OR "cats"[Title/Abstract] OR "dogs"[Title/Abstract] OR "crab"[Title/Abstract] OR "monkeys"[Title/Abstract] OR "rabbits"[Title/Abstract] OR "ducks"[Title/Abstract] OR "fowl"[Title/Abstract] OR "fowls"[Title/Abstract] OR "roots"[Title/Abstract] OR "bacteria"[Title/Abstract] OR "camel"[Title/Abstract] OR "Cohort Studies"[Title/Abstract] OR "cohort*"[Title/Abstract] OR "Cross-Sectional Studies"[Title/Abstract])

Result (N=25)

(("Ciprofloxacin"[MeSH Terms] OR "Ciprofloxacin"[Title/Abstract] OR "fluoroquinolone"[Title/Abstract] OR "quinolones"[Title/Abstract] OR "ofloxacin"[Title/Abstract] OR "levofloxacin"[Title/Abstract]) AND ("Brucellosis"[MeSH Terms] OR "Brucellosis"[Title/Abstract] OR "brucell*"[Title/Abstract] OR "brucella infection"[Title/Abstract] OR "malta fever"[Title/Abstract] OR "Gibraltar Fever"[Title/Abstract] OR "undulant fever"[Title/Abstract] OR "Mediterranean Fever"[Title/Abstract] OR "malta fever"[Title/Abstract] OR "Cyprus fever"[Title/Abstract] OR "rock fever"[Title/Abstract] OR "Bang's disease"[Title/Abstract]) AND ("clinical trial"[Title/Abstract] OR "single blinded method"[Title/Abstract] OR "double blinded method"[Title/Abstract] OR "cross over study"[Title/Abstract] OR "cross over stud*"[Title/Abstract] OR "random allocation"[Title/Abstract] OR "clinical trials as topic"[Title/Abstract] OR "comparative study"[Title/Abstract] OR "clinical trial*"[Title/Abstract] OR "controlled trial"[Title/Abstract] OR "Intervention"[Title/Abstract] OR "Randomized"[Title/Abstract] OR "Randomised"[Title/Abstract] OR "RCT"[Title/Abstract] OR "single blind"[Title/Abstract] OR "double blind"[Title/Abstract] OR "Parallel"[Title/Abstract] OR "Assignment"[Title/Abstract] OR "Cross-over"[Title/Abstract] OR "Randomly"[Title/Abstract])) NOT (("rats"[Title/Abstract] OR "mice"[Title/Abstract] OR "swine"[Title/Abstract] OR "mouse"[Title/Abstract] OR "mus"[Title/Abstract] OR "rat"[Title/Abstract] OR "in-vitro"[Title/Abstract] OR "in-vitro"[Title/Abstract] OR "pig"[Title/Abstract] OR "rabbit"[Title/Abstract] OR "rooster"[Title/Abstract] OR "cell"[Title/Abstract] OR "cells"[Title/Abstract] OR "cow"[Title/Abstract] OR "cows"[Title/Abstract] OR "suide"[Title/Abstract] OR "warthogs"[Title/Abstract] OR "broilers"[Title/Abstract] OR "roiler"[Title/Abstract] OR "in-vivo"[Title/Abstract] OR "in-vivo"[Title/Abstract] OR "rats"[Title/Abstract] OR "pigs"[Title/Abstract] OR "rats*"[Title/Abstract] OR "protein"[Title/Abstract] OR "goats"[Title/Abstract] OR "chicken"[Title/Abstract] OR "shrimp"[Title/Abstract] OR "sheep"[Title/Abstract] OR "cats"[Title/Abstract] OR "dogs"[Title/Abstract] OR "crab"[Title/Abstract] OR "monkeys"[Title/Abstract] OR "rabbits"[Title/Abstract] OR "ducks"[Title/Abstract] OR "fowl"[Title/Abstract] OR "fowls"[Title/Abstract] OR "roots"[Title/Abstract] OR "bacteria"[Title/Abstract] OR "camel"[Title/Abstract] OR "Cohort Studies"[Title/Abstract] OR "cohort*"[Title/Abstract] OR "Cross-Sectional Studies"[Title/Abstract]) AND ("meta analysis"[Publication Type] OR "systematic review"[Filter]))

| - Database | Search Strategy | N |
| --- | --- | --- |
| Pubmed | (("Ciprofloxacin"[MeSH Terms] OR "Ciprofloxacin"[Title/Abstract] OR "fluoroquinolone"[Title/Abstract] OR "quinolones"[Title/Abstract] OR "ofloxacin"[Title/Abstract] OR "levofloxacin"[Title/Abstract]) AND ("Brucellosis"[MeSH Terms] OR "Brucellosis"[Title/Abstract] OR "brucell*"[Title/Abstract] OR "brucella infection"[Title/Abstract] OR "malta fever"[Title/Abstract] OR "Gibraltar Fever"[Title/Abstract] OR "undulant fever"[Title/Abstract] OR "Mediterranean Fever"[Title/Abstract] OR "malta fever"[Title/Abstract] OR "Cyprus fever"[Title/Abstract] OR "rock fever"[Title/Abstract] OR "Bang's disease"[Title/Abstract]) AND ("clinical trial"[Title/Abstract] OR "single blinded method"[Title/Abstract] OR "double blinded method"[Title/Abstract] OR "cross over study"[Title/Abstract] OR "cross over stud*"[Title/Abstract] OR "random allocation"[Title/Abstract] OR "clinical trials as topic"[Title/Abstract] OR "comparative study"[Title/Abstract] OR "clinical trial*"[Title/Abstract] OR "controlled trial"[Title/Abstract] OR "Intervention"[Title/Abstract] OR "Randomized"[Title/Abstract] OR "Randomised"[Title/Abstract] OR "RCT"[Title/Abstract] OR "single blind"[Title/Abstract] OR "double blind"[Title/Abstract] OR "Parallel"[Title/Abstract] OR "Assignment"[Title/Abstract] OR "Cross-over"[Title/Abstract] OR "Randomly"[Title/Abstract])) NOT (("rats"[Title/Abstract] OR "mice"[Title/Abstract] OR "swine"[Title/Abstract] OR "mouse"[Title/Abstract] OR "mus"[Title/Abstract] OR "rat"[Title/Abstract] OR "in-vitro"[Title/Abstract] OR "in-vitro"[Title/Abstract] OR "pig"[Title/Abstract] OR "rabbit"[Title/Abstract] OR "rooster"[Title/Abstract] OR "cell"[Title/Abstract] OR "cells"[Title/Abstract] OR "cow"[Title/Abstract] OR "cows"[Title/Abstract] OR "suide"[Title/Abstract] OR "warthogs"[Title/Abstract] OR "broilers"[Title/Abstract] OR "roiler"[Title/Abstract] OR "in-vivo"[Title/Abstract] OR "in-vivo"[Title/Abstract] OR "rats"[Title/Abstract] OR "pigs"[Title/Abstract] OR "rats*"[Title/Abstract] OR "protein"[Title/Abstract] OR "goats"[Title/Abstract] OR "chicken"[Title/Abstract] OR "shrimp"[Title/Abstract] OR "sheep"[Title/Abstract] OR "cats"[Title/Abstract] OR "dogs"[Title/Abstract] OR "crab"[Title/Abstract] OR "monkeys"[Title/Abstract] OR "rabbits"[Title/Abstract] OR "ducks"[Title/Abstract] OR "fowl"[Title/Abstract] OR "fowls"[Title/Abstract] OR "roots"[Title/Abstract] OR "bacteria"[Title/Abstract] OR "camel"[Title/Abstract] OR "Cohort Studies"[Title/Abstract] OR "cohort*"[Title/Abstract] OR "Cross-Sectional Studies"[Title/Abstract]) AND ("meta analysis"[Publication Type] OR "systematic review"[Filter])) | 25 |
| Scopus | ( TITLE-ABS-KEY ( Fluoroquinolones ) OR TITLE-ABS-KEY ( Ciprofloxacin ) OR TITLE-ABS-KEY ( Levofloxacin ) OR TITLE-ABS-KEY ( Ofloxacin ) OR TITLE-ABS-KEY ( Fleroxacin ) OR TITLE-ABS-KEY ( Enoxacin ) OR TITLE-ABS-KEY ( Enrofloxacin ) OR TITLE-ABS-KEY ( Gatifloxacin ) OR TITLE-ABS-KEY ( Gemifloxacin ) OR TITLE-ABS-KEY ( Moxifloxacin ) OR TITLE-ABS-KEY ( Norfloxacin ) OR TITLE-ABS-KEY ( Pefloxacin ) ) AND ( TITLE-ABS-KEY ( Brucellosis ) OR TITLE-ABS-KEY ( brucell* ) OR TITLE-ABS-KEY ( brucella infection ) OR TITLE-ABS-KEY ( malta fever ) OR TITLE-ABS-KEY ( Gibraltar Fever ) OR TITLE-ABS-KEY ( undulant fever ) OR TITLE-ABS-KEY ( Mediterranean Fever ) OR TITLE-ABS-KEY ( Cyprus fever ) OR TITLE-ABS-KEY ( Bang's disease ) OR TITLE-ABS-KEY ( rock fever ) ) AND ( TITLE-ABS-KEY ( clinical trial ) OR TITLE-ABS-KEY ( single blinded method ) OR TITLE-ABS-KEY ( double blinded method ) OR TITLE-ABS-KEY ( cross over study ) OR TITLE-ABS-KEY ( cross over stud* ) OR TITLE-ABS-KEY ( random allocation ) OR TITLE-ABS-KEY ( comparative study ) or TITLE-ABS-KEY ( clinical trial* ) OR TITLE-ABS-KEY ( controlled trial ) OR TITLE-ABS-KEY ( Intervention ) OR TITLE-ABS-KEY ( Randomized ) OR TITLE-ABS-KEY ( Randomised ) OR TITLE-ABS-KEY ( RCT ) OR TITLE-ABS-KEY ( single blind ) OR TITLE-ABS-KEY ( double blind ) OR TITLE-ABS-KEY ( Parallel ) OR TITLE-ABS-KEY ( Assignment ) OR TITLE-ABS-KEY ( Cross-over ) OR TITLE-ABS-KEY ( Randomly ) ) | 172 |
| Web of science | #1: "Brucellosis" (Topic) or "brucell*" (Topic) or "brucella infection" (Topic) or "malta fever" (Topic) or "Gibraltar Fever" (Topic) or "undulant fever" (Topic) or "Mediterranean Fever" (Topic) or "Cyprus fever" (Topic) or "rock fever" (Topic) or "Bang's disease" (Topic)  #2: "Ciprofloxacin" (Topic) or "fluoroquinolone" (Topic) or "quinolones" (Topic) or "ofloxacin" (Topic) or "levofloxacin" (Topic) or "Enoxacin" (Topic) or "Enrofloxacin" (Topic) or "Gatifloxacin" (Topic) or "Gemifloxacin" (Topic) or "Moxifloxacin" (Topic) or "Norfloxacin" (Topic) or "Pefloxacin" (Topic)  #3: "clinical trial" (Topic) or "single blinded method" (Topic) or "double blinded method" (Topic) or "cross over study" (Topic) or "random allocation" (Topic) or "clinical trials as topic" (Topic) or "comparative study" (Topic) or "clinical trial*" (Topic) or "controlled trial" (Topic) or "Intervention" (Topic) or "Randomized" (Topic) or "Randomised" (Topic) or "RCT" (Topic) or "single blind" (Topic) or "double blind" (Topic) or "Parallel" (Topic) or "Assignment" (Topic) or "Cross-over" (Topic) or "Randomly" (Topic)  #1 AND #2 AND #3 | 34 |
| All |  | 231 |
| Duplicated |  | 54 |
| Remined after remove |  | 177 |
