# Supplementary material for: Efficacy and safety of fluoroquinolones in the treatment of human brucellosis: A systematic review and meta-analysis
Source: PLoS Negl Trop Dis. 2026 Oct 23;20(10):e0013822. doi: 10.1371/journal.pntd.0013822 (PMC13722316; doi:10.1371/journal.pntd.0013822)
Supplement: Supplementary file 3 [file pntd.0013822.s002.docx]

| **No.** | **Study** | **Reason for exclusion** |
| --- | --- | --- |
| 8 study | (Dash et al., 2012; Kurtaran et al., 2008; Liu et al., 2024; Liu et al., 2025; Miao & Li, 2024; Micozzi et al., 1990; Mortazavi et al., 2017; Sugihara et al., 2024) | case report |
| 14 study | (Anwar et al., 2023; Başaran et al., 2025; Cascio et al., 2012; Deftereos et al., 2022; Falagas & Bliziotis, 2006; Galvao et al., 2024; Heller et al., 2015; Helmy et al., 2017; Huang et al., 2023; Inayat et al., 2018; Maduranga et al., 2024; Pappas et al., 2004; Taamallah et al., 2021; Weil et al., 2003) | Review Article |
| 2 study | (Saltoglu et al., 2002; Taghvaee et al., 2011) | Prospective study |
| 10 study | (Al Dahouk et al., 2005; Baykam et al., 2004; Denk et al., 2015; Kilic et al., 2008; Lang et al., 1990; Pappas et al., 2006; Pechère, 1993; Rubinstein et al., 1991; Trujillano-Martín et al., 1999; Yamazhan et al., 2005) | In-vitro & In-vivo |

Al Dahouk, S., Hagen, R. M., Nöckler, K., Tomaso, H., Wittig, M., Scholz, H. C., Vergnaud, G., & Neubauer, H. (2005). Failure of a short-term antibiotic therapy for human Brucellosis using ciprofloxacin: A study on in vitro susceptibility of Brucella strains. *Chemotherapy*, *51*(6), 352-356.

Anwar, F. N., Roca, A. M., Khosla, I., Medakkar, S. S., Loya, A. C., Federico, V. P., Massel, D. H., Sayari, A. J., Lopez, G. D., & Singh, K. (2023). Antibiotic use in spine surgery: A narrative review based in principles of antibiotic stewardship [Article]. *North American Spine Society Journal*, *16*, Article 100278. <https://doi.org/10.1016/j.xnsj.2023.100278>

Başaran, S., Şimşek-Yavuz, S., Sarıcaoğlu, M. E., Aydın, M., Aygün, G., Azap, A., Azap, Ö., Benli, A., Demirtürk, N., Ergönül, Ö., Kocagül-Çelikbaş, A., Kuşçu, F., Özger, S., Kutlu, S. S., Türker, N., & Türkoğlu-Yılmaz, E. (2025). A Systematic Review and Analysis of Brucella Endocarditis Cases [Review]. *Anatolian Journal of Cardiology*, *29*(3), 111-117. <https://doi.org/10.14744/AnatolJCardiol.2025.4259>

Baykam, N., Esener, H., Ergönül, Ö., Eren, Ş., Çeli̇kbas, A. K., & Dokuzoǧuz, B. (2004). In vitro antimicrobial susceptibility of Brucella species. *International Journal of Antimicrobial Agents*, *23*(4), 405-407.

Cascio, A., De Caridi, G., Lentini, S., Benedetto, F., Stilo, F., Passari, G., Iaria, C., Spinelli, F., & Pappas, G. (2012). Involvement of the aorta in brucellosis: The forgotten, life-threatening complication. A systematic review [Review]. *Vector-Borne and Zoonotic Diseases*, *12*(10), 827-840. <https://doi.org/10.1089/vbz.2012.0965>

Dash, N., Al-Zarouni, M., Rattan, A., & Panigrahi, D. (2012). Misidentification of brucella melitensis as bergeyella zoohelcum by microscan walkaway®: A case report [Article]. *Medical Principles and Practice*, *21*(5), 495-497. <https://doi.org/10.1159/000338391>

Deftereos, S. G., Beerkens, F. J., Shah, B., Giannopoulos, G., Vrachatis, D. A., Giotaki, S. G., Siasos, G., Nicolas, J., Arnott, C., Patel, S., Parsons, M., Tardif, J. C., Kovacic, J. C., & Dangas, G. D. (2022). Colchicine in Cardiovascular Disease: In-Depth Review [Review]. *Circulation*, *145*(1), 61-78. <https://doi.org/10.1161/CIRCULATIONAHA.121.056171>

Denk, A., Demirdag, K., Kalkan, A., Ozden, M., Cetinkaya, B., & Kilic, S. S. (2015). In vitro activity of Brucella melitensis isolates to various antimicrobials in Turkey. *Infectious Diseases*, *47*(6), 364-369.

Falagas, M. E., & Bliziotis, I. A. (2006). Quinolones for treatment of human brucellosis: Critical review of the evidence from microbiological and clinical studies [Short survey]. *Antimicrobial Agents and Chemotherapy*, *50*(1), 22-33. <https://doi.org/10.1128/AAC.50.1.22-33.2006>

Galvao, E. L., Souza, K. M., de Freitas, M. G., Souza, M. R. F., Gonçalves, M. W. A., Cota, G., & Silva, S. N. (2024). Treatment of Childhood Brucellosis: A Systematic Review. *Pediatric Infectious Disease Journal*, *43*(9), 857-866. <https://doi.org/10.1097/INF.0000000000004389>

Heller, T., Bélard, S., Wallrauch, C., Carretto, E., Lissandrin, R., Filice, C., & Brunetti, E. (2015). Patterns of hepatosplenic brucella abscesses on cross-sectional imaging: A review of clinical and imaging features [Article]. *American Journal of Tropical Medicine and Hygiene*, *93*(4), 761-766. <https://doi.org/10.4269/ajtmh.15-0225>

Helmy, Y. A., El-Adawy, H., & Abdelwhab, E. M. (2017). A comprehensive review of common bacterial, parasitic and viral zoonoses at the human-animal interface in Egypt [Review]. *Pathogens*, *6*(3), Article 33. <https://doi.org/10.3390/pathogens6030033>

Huang, S., Wang, H., Li, F., Du, L., Fan, W., Zhao, M., Zhen, H., Yan, Y., Lu, M., Han, X., Li, Z., Li, M., An, S., Zhang, X., Zhen, Q., & Shui, T. (2023). Better efficacy of triple antibiotics therapy for human brucellosis: A systematic review and meta-analysis [Review]. *Plos Neglected Tropical Diseases*, *17*(9 September), Article e0011590. <https://doi.org/10.1371/journal.pntd.0011590>

Inayat, F., Mahboob, M., Ali, N. S., Bokhari, S. R. A., & Ashraf, A. (2018). Brucellosis in renal transplant recipients: A comparative review of 5 cases [Article]. *BMJ Case Reports*, *2018*, Article 225865. <https://doi.org/10.1136/bcr-2018-225865>

Kilic, S., Dizbay, M., & Cabadak, H. (2008). In vitro activity of tigecycline, tetracycline and fluoroquinolones against Brucella melitensis. *Journal of Chemotherapy*, *20*(1), 33-37.

Kurtaran, B., Sarpel, T., Tasova, Y., Candevir, A., Saltoglu, N., Inal, A. S., & Aksu, H. S. Z. (2008). Brucellar and tuberculous spondylitis in 87 adult patients: A descriptive and comparative case series [Article]. *Infectious Diseases in Clinical Practice*, *16*(3), 166-173. <https://doi.org/10.1097/IPC.0b013e318168ffb3>

Lang, R., Raoul, R., Sacks, T., & Shapiro, M. (1990). Failure of prolonged treatment with dprofloxadn in acute infections dueto brucella melitensis [Article]. *Journal of Antimicrobial Chemotherapy*, *26*(6), 841-846. <https://doi.org/10.1093/jac/26.6.841>

Liu, B., Zhang, Y., Yuan, Z., & Zhang, Q. (2024). Cervical Brucella Spondylitis: A Case Report on Diagnosis and Surgical Management [Article]. *Infection and Drug Resistance*, *17*, 3537-3545. <https://doi.org/10.2147/IDR.S474589>

Liu, H., Li, Q., Ouyang, X., Li, Q., Min, Y., & Dai, L. (2025). Diagnosis of early neurobrucellosis using metagenomic next-generation sequencing of the cerebrospinal fluid in nonepidemic zone: Case report and lecture review [Article]. *Medicine (United States)*, *104*(7), e41481. <https://doi.org/10.1097/MD.0000000000041481>

Maduranga, S., Valencia, B. M., Li, X., Moallemi, S., & Rodrigo, C. (2024). A systematic review and meta-analysis of comparative clinical studies on antibiotic treatment of brucellosis [Article]. *Scientific Reports*, *14*(1), Article 19037. <https://doi.org/10.1038/s41598-024-69669-w>

Miao, Y., & Li, X. (2024). A case report of acute renal failure caused by anti-brucellosis treatment [Article]. *Medicine (United States)*, *103*(13), E37560. <https://doi.org/10.1097/MD.0000000000037560>

Micozzi, A., Venditti, M., Gentile, G., Alessandri, N., Santero, M., & Martino, P. (1990). Successful treatment of Brucella melitensis endocarditis with pefloxacin. *European Journal of Clinical Microbiology and Infectious Diseases*, *9*(6), 440-442.

Mortazavi, S. J., Sobhan, M. R., & Mazoochy, H. (2017). Brucella arthritis following total knee arthroplasty in a patient with hemophilia: A case report [Article]. *Archives of Bone and Joint Surgery*, *5*(5), 342-346. <https://doi.org/10.22038/abjs.2017.21696.1559>

Pappas, G., Christou, L., Akritidis, N., & Tsianos, E. V. (2006). Quinolones for brucellosis: Treating old diseases with new drugs [Editorial]. *Clinical Microbiology and Infection*, *12*(9), 823-825. <https://doi.org/10.1111/j.1469-0691.2006.01442.x>

Pappas, G., Seitaridis, S., Akritidis, N., & Tsianos, E. (2004). Treatment of brucella spondylitis: Lessons from an impossible meta-analysis and initial report of efficacy of a fluoroquinolone-containing regimen [Article]. *International Journal of Antimicrobial Agents*, *24*(5), 502-507. <https://doi.org/10.1016/j.ijantimicag.2004.05.003>

Pechère, J.-C. (1993). Quinolones in intracellular infections. *Drugs*, *45*(Suppl 3), 29-36.

Rubinstein, E., Lang, R., Shasha, B., Hagar, B., Diamanstein, L., Joseph, G., Anderson, M., & Harrison, K. (1991). In vitro susceptibility of Brucella melitensis to antibiotics. *Antimicrobial Agents and Chemotherapy*, *35*(9), 1925-1927.

Saltoglu, N., Tasova, Y., Inal, A. S., Seki, T., & Aksu, H. S. (2002). Efficacy of rifampicin plus doxycycline versus rifampicin plus quinolone in the treatment of brucellosis [Article]. *Saudi Medical Journal*, *23*(8), 921-924. <https://www.scopus.com/inward/record.uri?eid=2-s2.0-0036700523&partnerID=40&md5=3511a3589ecaa6c8509ff99cb42fda7f>

Sugihara, T., Sano, Y., Ueki, T., Ishimura, T., Takeda, M., Kiriyama, Y., Mori, Y., Sakao, N., Otani, S., & Izutani, H. (2024). Successful conservative management of advanced pyogenic sternoclavicular joint arthritis with osteomyelitis and pulmonary infiltration: a case report [Article]. *Journal of Medical Case Reports*, *18*(1), Article 394. <https://doi.org/10.1186/s13256-024-04684-z>

Taamallah, K., Hammami, F., Gharsallah, H., Koubaa, M., Ben Jemaa, M., & Fehri, W. (2021). Brucella prosthetic valve endocarditis: A systematic review [Review]. *Journal of the Saudi Heart Association*, *33*(3), 198-212. <https://doi.org/10.37616/2212-5043.1257>

Taghvaee, M. R., Nozadi, M., & Hassani, M. (2011). A comparison between doxycycline-rifampin and ciprofloxacin-rifampin regimens in the treatment of acute Brucellosis. *Indian Journal of Medical Sciences*, *65*(10), 436.

Trujillano-Martín, I., García-Sánchez, E., Martínez, I. a. M., Fresnadillo, M. a. J., García-Sánchez, J. E. a., & García-Rodríguez, J. A. n. (1999). In vitro activities of six new fluoroquinolones against Brucella melitensis. *Antimicrobial Agents and Chemotherapy*, *43*(1), 194-195.

Weil, Y., Mattan, Y., Liebergall, M., & Rahav, G. (2003). Brucella prosthetic joint infection:: A report of 3 cases and a review of the literature. *Clinical Infectious Diseases*, *36*(7), E81-E86. <https://doi.org/10.1086/368084>

Yamazhan, T., Aydemir, Ş., Tünger, A., Serter, D., & Gökengin, D. (2005). In vitro activities of various antimicrobials against Brucella melitensis strains in the Aegean region in Turkey. *Medical Principles and Practice*, *14*(6), 413-416.
