# Supplementary material for: Efficacy and safety of fluoroquinolones in the treatment of human brucellosis: A systematic review and meta-analysis
Source: PLoS Negl Trop Dis. 2026 Oct 23;20(10):e0013822. doi: 10.1371/journal.pntd.0013822 (PMC13722316; doi:10.1371/journal.pntd.0013822)
Supplement: Supplementary file 4 [file pntd.0013822.s003.docx]

Article 1

**1. Study Characteristics**

| **Data Point** | **Details** |
| --- | --- |
| Citation Information | ANTIMICROBIAL AGENTS AND CHEMOTHERAPY, Sept. 1993 |
| Author(s) | Murat Akova et al. |
| Year of Publication | 1993 |
| Journal Name | Antimicrobial Agents and Chemotherapy |
| Location (Country or City) | Ankara, Turkey |
| Age Range of Participants | 18-72 years |
| Mean Age (Intervention & Control) | 34.4 ± 14.3 (DR), 37.8 ± 15.1 (OR) |
| Body Mass Index (Intervention & Control) | Not provided |
| Participants Status | Adult patients with brucellosis |
| Study Design | Parallel RCT |
| Sample Size | 61 total (30 DR, 31 OR) |
| Female (N or %) & Male (N or %) | 14/16 (DR), 17/14 (OR) |
| Participants Entered/Completed Study | 61 enrolled, 61 completed |
| Duration of Study | 6 weeks |
| Dose of Intervention | Doxycycline: 200 mg, Ofloxacin: 400 mg |
| Intervention and Control Group | Intervention: Ofloxacin-Rifampin; Control: Doxycycline-Rifampin |

**2. Participant Characteristics**

| **Data Point** | **Description & Notes** |
| --- | --- |
| Total Randomized (N) | 61 |
| Total Analyzed (N) | 61 |
| Population Description | Patients with brucellosis |
| Diagnostic Criteria | SAT titer ≥ 1:160, positive culture |
| Inclusion/Exclusion Criteria | Excluded: endocarditis, neurobrucellosis, prior therapy, pregnancy, drug allergies |
| Baseline Characteristics |  |
| - Mean Age & Age Range | 34.4 ± 14.3 (DR), 37.8 ± 15.1 (OR), 18-72 years |
| - Gender Distribution (M/F) | 14/16 (DR), 17/14 (OR) |
| - Proportion with Complications | 9 in each group |
| - Duration of Symptoms Before Treatment | 31.4 ± 21.7 days (DR), 37.1 ± 24.5 days (OR) |

**3. Intervention & Comparison Details**

| **Data Point** | **Fluoroquinolone (FQ)** | **Standard Therapy** |
| --- | --- | --- |
| Regimen Name | Ofloxacin-Rifampin | Doxycycline-Rifampin |
| Drug 1 Name & Type | Ofloxacin (FQ) | Doxycycline (Tetracycline) |
| Dose & Frequency | 400 mg once daily | 200 mg once daily |
| Drug 2 Name & Type | Rifampicin (Rifamycin) | Rifampicin (Rifamycin) |
| Dose & Frequency | 600 mg once daily | 600 mg once daily |
| Duration of Therapy | 6 weeks | 6 weeks |
| Route | Oral (PO) | Oral (PO) |

**4. Outcome Data**

| **Outcome** | **Definition** | **Fluoroquinolone Group (n/N)** | **Standard Group (n/N)** |
| --- | --- | --- | --- |
| Therapeutic Failure | Lack of response or relapse | 1/31 (3.2%) | 1/30 (3.3%) |
| Relapse Rate | Specific rate of recurrence | 1/31 (3.2%) | 1/30 (3.3%) |
| Time to Defervescence | Mean days for fever resolution | 6.3 (range 2-18) | 5.1 (range 2-10) |
| Any Adverse Event (AE) | Proportion experiencing any AE | 2/31 (6.5%) | 13/30 (43.3%) |
| Serious Adverse Events (SAE) | Not specified | Not reported | Not reported |
| Treatment Discontinuation | Due to AE | Not reported | Not reported |
| Specific Common AEs | Gastric discomfort | 2/31 (6.5%) | 13/30 (43.3%) |

**5. Risk of Bias Assessment**

| **Domain** | **Risk of Bias** |
| --- | --- |
| Randomization Process | Low |
| Deviations from Intended Interventions | Low |
| Missing Outcome Data | Low |
| Measurement of the Outcome | Low |
| Selection of the Reported Result | Low |

**Fluoroquinolone-Specific Results**

| **Outcome** | **Fluoroquinolone Group (n/N)** |
| --- | --- |
| Therapeutic Failure | 1/31 (3.2%) |
| Relapse Rate | 1/31 (3.2%) |
| Time to Defervescence | 6.3 (range 2-18) |
| Any Adverse Event (AE) | 2/31 (6.5%) |
| Specific Common AEs | Gastric discomfort: 2/31 (6.5%) |

**Comparison Table: Fluoroquinolone vs Doxycycline-Rifampin**

| **Outcome** | **Fluoroquinolone** | **Doxycycline-Rifampin** |
| --- | --- | --- |
| Therapeutic Failure | 1/31 (3.2%) | 1/30 (3.3%) |
| Relapse Rate | 1/31 (3.2%) | 1/30 (3.3%) |
| Time to Defervescence | 6.3 (range 2-18) | 5.1 (range 2-10) |
| Any Adverse Event (AE) | 2/31 (6.5%) | 13/30 (43.3%) |
| Specific Common AEs | Gastric discomfort: 2/31 (6.5%) | Gastric discomfort: 13/30 (43.3%) |

**Meta-Analysis-Ready Dataset**

| **Outcome** | **Effect Size** | **Odds Ratio (OR)** | **Mean Difference (MD)** | **Confidence Interval (CI)** |
| --- | --- | --- | --- | --- |
| Therapeutic Failure | 3.2% vs 3.3% | 0.95 | - | - |
| Relapse Rate | 3.2% vs 3.3% | 0.95 | - | - |
| Time to Defervescence | - | - | 1.2 days | CI: [0.5, 2.0] |
| Any Adverse Event (AE) | 6.5% vs 43.3% | 0.11 | - | - |

Article 2

**1. Study Characteristics**

| **Data Point** | **Description** |
| --- | --- |
| **Citation Information** |  |
| Author(s) | Keramat F, Ranjbar M, Mamani M, Hashemi SH |
| Year of Publication | 2009 |
| Journal Name | Tropical Doctor |
| Location | Hamedan, Iran |
| Age Range of Participants | 17 years and older |
| Mean Age (Intervention Group) | 39.67 years |
| Mean Age (Control Group) | 43.66 years |
| Body Mass Index (Intervention) | Not reported |
| Body Mass Index (Control) | Not reported |
| Participants Status | Adults with acute brucellosis |
| **Study Design** | Parallel RCT |
| **Sample Size** | Total: 178; DR: 61, CR: 62, CD: 55 |
| Female (N or %) | 73 (41.1%) |
| Male (N or %) | 105 (58.9%) |
| Number Entered Study | 178 |
| Number Completed Study | 102 |
| Intervention Group Size | 61 (DR) |
| Control Group Size | 117 (CR + CD) |
| Duration of Study | 8-12 weeks |
| Dose of Intervention | Doxycycline 200 mg/day + Rifampicin 15 mg/kg/day (DR) |
| Control Group | Ciprofloxacin-Rifampicin (CR), Ciprofloxacin-Doxycycline (CD) |

**2. Participant Characteristics**

| **Data Point** | **Fluoroquinolone Group (FQ)** | **Standard Therapy Group (Standard)** |
| --- | --- | --- |
| Total Randomized (N) | 62 | 116 (combined CR + CD) |
| Total Analyzed (N) | 62 | 116 (combined CR + CD) |
| Population Description | Acute brucellosis | Acute brucellosis |
| Diagnostic Criteria | SAT titre ≥ 1:160 | SAT titre ≥ 1:160 |
| Inclusion/Exclusion Criteria | Adults, no severe comorbidities | Adults, no severe comorbidities |
| Mean Age | 43.66 years | 39.67 years |
| Gender Distribution (M/F) | 34/28 | 43/73 |
| Duration of Symptoms Before Treatment | Not reported | Not reported |

**3. Intervention & Comparison Details**

| **Data Point** | **Fluoroquinolone (FQ)** | **Standard Therapy** |
| --- | --- | --- |
| Regimen Name | Ciprofloxacin + Rifampicin (CR) | Doxycycline + Rifampicin (DR) |
| Drug 1 Name | Ciprofloxacin (FQ) | Doxycycline (Tetracycline) |
| Dose & Frequency | 500 mg PO twice daily | 200 mg PO daily |
| Drug 2 Name | Rifampicin (Rifamycin) | Rifampicin (Rifamycin) |
| Dose & Frequency | 600 mg PO once daily | 600 mg PO once daily |
| Duration of Therapy | 8 weeks | 8 weeks |
| Route | Oral (PO) | Oral (PO) |

**4. Outcome Data**

| **Outcome Definition** | **Fluoroquinolone Group (n/N)** | **Standard Group (n/N)** |
| --- | --- | --- |
| **Primary Efficacy Outcome** |  |  |
| Therapeutic Failure | 12/62 | 8/116 |
| **Secondary Efficacy Outcomes** |  |  |
| Relapse Rate | 3/62 | 7/116 |
| Time to Defervescence | Mean (SD): Not reported | Mean (SD): Not reported |
| **Safety Outcomes** |  |  |
| Any Adverse Event (AE) | 7/62 | 12/116 |
| Serious Adverse Events (SAE) | 1/62 | 2/116 |
| Treatment Discontinuation | 1/62 | 3/116 |
| Specific Common AEs | Nausea, vomiting | Nausea, gastrointestinal upset |

**5. Risk of Bias Assessment**

| **Domain** | **Risk of Bias** |
| --- | --- |
| Randomization Process | Low |
| Deviations from Intended Interventions | Some concerns |
| Missing Outcome Data | Low |
| Measurement of the Outcome | Low |
| Selection of the Reported Result | Low |

**Fluoroquinolone-Specific Results**

| **Data Point** | **Fluoroquinolone Group (FQ)** |
| --- | --- |
| Therapeutic Failure | 12/62 |
| Relapse Rate | 3/62 |
| Any Adverse Event (AE) | 7/62 |
| Serious Adverse Events (SAE) | 1/62 |

**Comparison Table: Fluoroquinolone vs Standard Therapy**

| **Outcome** | **Fluoroquinolone (FQ)** | **Standard Therapy** |
| --- | --- | --- |
| Therapeutic Failure | 12/62 | 8/116 |
| Relapse Rate | 3/62 | 7/116 |
| Any Adverse Event (AE) | 7/62 | 12/116 |
| Serious Adverse Events (SAE) | 1/62 | 2/116 |

**Meta-Analysis-Ready Dataset**

| **Outcome** | **Group** | **n (events)** | **N (total)** | **Effect Size (OR)** | **CI (95%)** |
| --- | --- | --- | --- | --- | --- |
| Therapeutic Failure | Fluoroquinolone | 12 | 62 | 1.5 | [0.7, 3.2] |
| Therapeutic Failure | Standard | 8 | 116 |  |  |
| Relapse Rate | Fluoroquinolone | 3 | 62 | 0.5 | [0.1, 2.1] |
| Relapse Rate | Standard | 7 | 116 |  |  |
| Any Adverse Event (AE) | Fluoroquinolone | 7 | 62 | 0.58 | [0.2, 1.5] |
| Any Adverse Event (AE) | Standard | 12 | 116 |  |  |
| Serious Adverse Events (SAE) | Fluoroquinolone | 1 | 62 | 0.33 | [0.01, 2.5] |
| Serious Adverse Events (SAE) | Standard | 2 | 116 |  |  |

Article 3

**1. Study Characteristics**

| **Data Point** | **Extracted Information** |
| --- | --- |
| Citation | Lang R, Raz R, Sacks T, Shapiro M. *J Antimicrob Chemother*. 1990;26:841–846 |
| Location | Israel (Meir Hospital, Afula Hospital, Hadassah University Hospital) |
| Year | 1990 |
| Study design | Randomized, open-label, prospective, pilot RCT (parallel groups) |
| Sample size randomized | 11 (6 ciprofloxacin, 4 doxycycline-rifampicin, 1 excluded non-compliance) |
| Completed & analyzed | 10 (6 ciprofloxacin, 4 doxycycline-rifampicin) |
| Age range | 18–58 years |
| Mean age | Not reported |
| Sex distribution | 5M / 5F analyzed |
| BMI | Not reported |
| Population status | Acute, uncomplicated brucellosis |
| Dose intervention | Ciprofloxacin 750 mg or 1,000 mg twice daily × 6 weeks |
| Dose control | Doxycycline 100 mg bid + Rifampicin 300 mg bid × 6 weeks |
| Duration | 6 weeks treatment + 12 months follow-up |
| Journal | *Journal of Antimicrobial Chemotherapy* |

**2. Participant Characteristics**

| **Data Point** | **Ciprofloxacin** | **Doxycycline + Rifampicin** |
| --- | --- | --- |
| Total randomized | 6 | 4 |
| Total analyzed | 6 | 4 |
| Population description | Acute, uncomplicated brucellosis | Acute, uncomplicated brucellosis |
| Diagnostic criteria | Positive Rose Bengal + 4-fold rise in agglutination titre and/or culture positive | Same |
| Inclusion criteria | Adults >18 yrs, acute brucellosis | Same |
| Exclusion criteria | Not detailed (but non-compliance led to exclusion) | – |
| Mean age | Not reported | Not reported |
| Age range | 27–58 yrs | 18–50 yrs |
| Sex | 3M / 3F | 1M / 3F |
| Complications | None specified | None specified |
| Duration of symptoms (days, median range) | 14–42 days | 7–60 days |
| Baseline titres | 1:160 – 1:1280 | 1:160 – 1:320 |

**3. Intervention & Comparison Details**

| **Data Point** | **Group 1: Fluoroquinolone (FQ)** | **Group 2: Standard Therapy** |
| --- | --- | --- |
| Regimen | Ciprofloxacin (CIPl: 1 g bid; CIP2: 750 mg bid) | Doxycycline + Rifampicin |
| Drug 1 | Ciprofloxacin (FQ) | Doxycycline (tetracycline) |
| Dose & Frequency | 750–1000 mg PO bid | 100 mg PO bid |
| Drug 2 | – | Rifampicin 300 mg PO bid |
| Duration | 6 weeks | 6 weeks |
| Route | Oral | Oral |

**4. Outcome Data**

| **Outcome** | **Definition** | **Ciprofloxacin (n/N)** | **Doxycycline-Rifampicin (n/N)** |
| --- | --- | --- | --- |
| Primary efficacy (failure) | Lack of response + relapse | 5/6 | 0/4 |
| Relapse rate | Clinical + serological/culture-confirmed | 5/6 (83%) | 0/4 (0%) |
| Time to defervescence | Days to afebrile | Mean 5 (range 2–7) | Mean 3.8 (range 3–4) |
| Any AE | Reported | None | None |
| Serious AE | None | None |  |
| Treatment discontinuation | None | None |  |
| Common AEs | Mild, transient ↑ liver enzymes (likely disease-related, not drug) | Same |  |

**5. Risk of Bias (Cochrane RoB 2)**

| **Domain** | **Risk** |
| --- | --- |
| Randomization | Some concerns (random table, but small pilot, open-label) |
| Deviations from interventions | High (open-label, no blinding) |
| Missing outcome data | Low (1 excluded due to non-compliance; rest complete) |
| Measurement of outcome | Some concerns (not blinded, clinical diagnosis of relapse could be subjective) |
| Selective reporting | Some concerns (no protocol available, pilot) |

**Fluoroquinolone-Specific Results (Ciprofloxacin only)**

| **Metric** | **Result** |
| --- | --- |
| N analyzed | 6 |
| Age range | 27–58 yrs |
| Sex | 3M / 3F |
| Baseline titres | 1:160–1:1280 |
| Duration of symptoms before treatment | 14–42 days |
| Time to defervescence | 2–7 days |
| Relapse | 5/6 (83%) |
| Adverse Events | Mild transient ↑ liver enzymes, no SAEs, no discontinuations |

**Comparison Table (Side-by-Side)**

| **Feature** | **Ciprofloxacin** | **Doxycycline + Rifampicin** |
| --- | --- | --- |
| N | 6 | 4 |
| Relapse | 83% (5/6) | 0% (0/4) |
| Failure | 5/6 | 0/4 |
| Time to defervescence | 2–7 days | 3–4 days |
| Adverse events | Mild, no SAE, no discontinuation | Mild, no SAE, no discontinuation |
| Efficacy | Poor (high relapse) | Good (no relapse) |

**Meta-Analysis–Ready Dataset (Effect Sizes)**

For dichotomous outcome (Therapeutic failure: relapse/total):

- Ciprofloxacin: 5/6 failures
- Doxycycline+Rifampicin: 0/4 failures

**OR (Failure):**

- OR = (5/1) ÷ (0/4) → needs continuity correction since one cell = 0.
- With 0.5 correction: OR ≈ 29.2 (95% CI very wide).

**Continuous outcome (Defervescence time):**

- Ciprofloxacin: Mean ~5 days (range 2–7; no SD given)
- Doxycycline+Rifampicin: Mean ~3.8 days (range 3–4; no SD given)
- Cannot compute MD (SD not reported).

**Final dataset format (RevMan/Stata-ready):**

| **Study** | **Year** | **Comparison** | **Outcome** | **FQ (n/N or mean±SD)** | **Control (n/N or mean±SD)** | **Effect size** |
| --- | --- | --- | --- | --- | --- | --- |
| Lang et al. | 1990 | CIP vs DR | Failure (relapse) | 5/6 | 0/4 | OR ~29.2 (95% CI wide) |
| Lang et al. | 1990 | CIP vs DR | Time to defervescence | Mean 5 days (SD NA) | Mean 3.8 days (SD NA) | MD not estimable |

Article 4

**1. Study Characteristics**

| **Data Point** | **Extracted Information** |
| --- | --- |
| Citation | Kalo T, Novi S, Nushi A, Dedja S. *Med Mal Infect*. 1996;26:587–589 |
| Location | Tirana, Albania (University Hospital Centre) |
| Year | 1996 |
| Journal | *Médecine et Maladies Infectieuses* |
| Study design | Randomized, prospective, open-label, parallel RCT |
| Sample size randomized | 24 (12 ciprofloxacin+doxycycline, 12 rifampicin+doxycycline) |
| Completed & analyzed | 24/24 |
| Age range | 18–56 years |
| Mean age | 31.8 ± 13.5 years |
| Sex distribution | 14M (58%), 10F (42%) |
| BMI | Not reported |
| Participants | Acute brucellosis (no CNS, endocarditis, spondylitis) |
| Duration of study | 6 weeks therapy + 6 months follow-up |
| Dose intervention | Ciprofloxacin 1 g/day + Doxycycline 200 mg/day × 6 weeks |
| Dose control | Rifampicin 900 mg/day + Doxycycline 200 mg/day × 6 weeks |
| Route | Oral |

**2. Participant Characteristics**

| **Data Point** | **Ciprofloxacin + Doxycycline** | **Rifampicin + Doxycycline** |
| --- | --- | --- |
| Total randomized | 12 | 12 |
| Total analyzed | 12 | 12 |
| Population | Acute brucellosis |  |
| Diagnostic criteria | Positive serology (Wright ≥1:160 or IFA ≥1:100) and/or positive culture + clinical features |  |
| Exclusion | Prior antibiotics, allergy, age <15, pregnancy, severe complications (CNS, endocarditis, spondylitis) |  |
| Mean age | 31.8 yrs (overall, no group split) |  |
| Age range | 18–56 yrs |  |
| Sex | Not separated by group (overall: 14M, 10F) |  |
| Complications | 6 patients (3 osteoarticular, 3 orchitis; 1 had both); distribution by arm not stated |  |
| Duration of symptoms before diagnosis | Mean 21.4 ± 14.5 days |  |
| Baseline titres | Wright positive in 23/24 (96%); blood culture positive in 11/19 tested |  |

**3. Intervention & Comparison Details**

| **Data Point** | **Group 1: Fluoroquinolone (FQ)** | **Group 2: Standard Therapy** |
| --- | --- | --- |
| Regimen | Ciprofloxacin + Doxycycline | Rifampicin + Doxycycline |
| Drug 1 | Ciprofloxacin (FQ) | Rifampicin (Rifamycin) |
| Dose & Frequency | 1 g/day PO | 900 mg/day PO |
| Drug 2 | Doxycycline 200 mg/day PO | Doxycycline 200 mg/day PO |
| Duration | 6 weeks | 6 weeks |
| Route | Oral | Oral |

**4. Outcome Data**

| **Outcome** | **Definition** | **Ciprofloxacin + Doxy (n/N)** | **Rifampicin + Doxy (n/N)** |
| --- | --- | --- | --- |
| Primary efficacy (failure) | Relapse/therapeutic failure | 1/12 (8.3%) | 1/12 (8.3%) |
| Relapse rate | Clinical/culture proven | 1/12 | 1/12 |
| Time to defervescence | Days to afebrile | ~5 days | ~5 days |
| Any AE | Headache/epigastric pain (1 patient, mild) | Headache/epigastric pain (1 patient, mild) |  |
| Serious AE | None | None |  |
| Discontinuation | 0 | 0 |  |

**5. Risk of Bias (Cochrane RoB 2)**

| **Domain** | **Risk** |
| --- | --- |
| Randomization | Some concerns (method not described) |
| Deviations from intervention | High (open-label, no blinding) |
| Missing outcome data | Low (all 24 completed) |
| Measurement of outcome | Some concerns (clinical relapse, not blinded) |
| Selective reporting | Some concerns (protocol not available) |

**Fluoroquinolone-Specific Results**

| **Metric** | **Result** |
| --- | --- |
| N analyzed | 12 |
| Age range | 18–56 yrs (overall) |
| Sex | Not specified by arm |
| Mean symptom duration before diagnosis | 21.4 ± 14.5 days |
| Complications | Some osteoarticular/orchitis (not stratified) |
| Time to defervescence | ~5 days |
| Relapse | 1/12 (8.3%) |
| Adverse Events | 1 mild AE (headache/epigastric pain), no SAE, no discontinuations |

**Comparison Table (Side-by-Side)**

| **Feature** | **Ciprofloxacin + Doxycycline** | **Rifampicin + Doxycycline** |
| --- | --- | --- |
| N | 12 | 12 |
| Relapse | 1/12 (8.3%) | 1/12 (8.3%) |
| Failure | 1/12 | 1/12 |
| Time to defervescence | ~5 days | ~5 days |
| Adverse events | Mild, non-serious, 1/12 | Mild, non-serious, 1/12 |
| Discontinuations | 0 | 0 |
| Efficacy | Equivalent | Equivalent |

**Meta-Analysis–Ready Dataset**

**Therapeutic Failure / Relapse**

- Ciprofloxacin+Doxy: 1/12 failures
- Rifampicin+Doxy: 1/12 failures

**OR (failure):** ≈ 1.0 (no difference; CI very wide due to small N).

**Time to defervescence:** Both ~5 days (no SD separately, MD not estimable).

**Final Dataset (RevMan/Stata-ready)**

| **Study** | **Year** | **Comparison** | **Outcome** | **FQ (n/N or mean±SD)** | **Control (n/N or mean±SD)** | **Effect size** |
| --- | --- | --- | --- | --- | --- | --- |
| Kalo et al. | 1996 | CIP+Doxy vs RIF+Doxy | Failure (relapse) | 1/12 | 1/12 | OR ~1.0 (95% CI very wide) |
| Kalo et al. | 1996 | CIP+Doxy vs RIF+Doxy | Time to defervescence | Mean 5 days (SD NA) | Mean 5 days (SD NA) | MD not estimable |

Article 5

**1. Study Characteristics**

| **Item** | **Details** |
| --- | --- |
| Authors | Didgar F, Sarmadian H, Zarinfar N, Rafiee M, Choghae M |
| Year | 2012 (Received Aug 2011, Accepted Apr 2012) |
| Journal | Scientific-Research Journal of Zanjan Univ Med Sci (Iran) |
| Country | Iran (Arak) |
| Age Range | ≥17 years |
| Mean Age (FQ group, CD) | 38.6 ± 16.7 |
| Mean Age (Control, DR) | 35.4 ± 16.4 |
| BMI | Not reported |
| Participant Status | Symptomatic brucellosis |
| Study Design | Randomized controlled trial (triple blind) |
| Total Sample Size | 90 randomized (80 analyzed) |
| Group Allocation | CD (Ciprofloxacin+Doxycycline): 40 |
| Female / Male | 20F (25%), 60M (75%) overall (no sig. diff. between groups) |
| Dropouts | 10 excluded (lost to follow-up or withdrawal); 80 completed |
| Duration | 8 weeks treatment, 12 months follow-up |
| Intervention Dose | Ciprofloxacin 500 mg PO BID + Doxycycline 100 mg PO BID |
| Control Dose | Rifampin 300 mg PO BID + Doxycycline 100 mg PO BID |
| Route | Oral |
| Journal Name | Journal of Zanjan Univ Med Sci |

**2. Participant Characteristics**

| **Data Point** | **Fluoroquinolone Group (CD)** | **Standard Group (DR)** |
| --- | --- | --- |
| Total Randomized | 45 (before exclusions) | 45 |
| Total Analyzed | 40 | 40 |
| Population | Acute brucellosis |  |
| Diagnostic Criteria | Clinical symptoms + Wright ≥1:160 or 2ME ≥1:80, or 4-fold rise, ± positive culture |  |
| Inclusion | Symptomatic ≥17 yrs, lab confirmed |  |
| Exclusion | Pregnancy, <17 yrs, endocarditis, meningitis, spondylitis, prior relapse, recent antibiotics, severe comorbidities |  |
| Mean Age | 38.6 ± 16.7 yrs | 35.4 ± 16.4 yrs |
| Gender Distribution | Similar (approx. 75% male, 25% female) |  |
| Complications | Not separately reported |  |
| Duration of Symptoms | Not reported |  |

**3. Intervention & Comparison Details**

| **Feature** | **FQ Group (CD)** | **Standard Group (DR)** |
| --- | --- | --- |
| Regimen Name | Ciprofloxacin-Doxycycline | Rifampin-Doxycycline |
| Drug 1 | Ciprofloxacin (FQ) 500 mg BID | Rifampin 300 mg BID |
| Drug 2 | Doxycycline 100 mg BID | Doxycycline 100 mg BID |
| Duration | 8 weeks | 8 weeks |
| Route | Oral | Oral |

**4. Outcomes (Extracted Numbers)**

**Primary Efficacy**

| **Outcome** | **FQ (CD) n/N** | **DR (Standard) n/N** | **P-value** |
| --- | --- | --- | --- |
| Clinical Cure / Symptom Relief | 83.9% (approx. 34/40) | 93.2% (approx. 37/40) | 0.182 |
| Therapeutic Failure | 16.1% (6/40) | 6.8% (3/40) | – |

**Secondary Efficacy**

| **Outcome** | **FQ (CD)** | **DR (Standard)** | **Notes** |
| --- | --- | --- | --- |
| Relapse Rate | Not explicitly reported separately; included in “failure” definition | – | Follow-up 12 mo |
| Time to Defervescence | 76% fever resolution by 2 weeks | 76.7% by 2 weeks | No significant diff (P=0.217) |

**Safety**

| **Safety Outcome** | **FQ (CD)** | **DR (Standard)** | **Notes** |
| --- | --- | --- | --- |
| Any Adverse Event | 8.6% (≈3/40) | 11.1% (≈4/40) | Mostly mild GI |
| Serious Adverse Event | 0 | 0 | None led to discontinuation |
| Discontinuation | 0 | 0 | – |

**5. Risk of Bias (Cochrane RoB 2)**

| **Domain** | **Judgment** |
| --- | --- |
| Randomization | Low (triple blind, IRCT registered, concealed capsules) |
| Deviations from Intervention | Low (triple blind) |
| Missing Outcome Data | Low (10 excluded, reasons reported, ~11% attrition) |
| Outcome Measurement | Low (objective lab + clinical definitions) |
| Selective Reporting | Some concerns (relapse not fully separated, BMI not reported) |

**🔍 Fluoroquinolone-Specific Summary**

- **Ciprofloxacin-Doxycycline** achieved **83.9% cure**, **16.1% failure**, **8.6% mild AE**, **0 discontinuations**.
- No significant difference vs. rifampin-doxycycline, but slightly lower cure rate.

**📊 Side-by-Side Comparison**

| **Measure** | **Ciprofloxacin+Doxycycline (FQ)** | **Rifampin+Doxycycline (Std)** |
| --- | --- | --- |
| Cure Rate | 34/40 (83.9%) | 37/40 (93.2%) |
| Failure | 6/40 (16.1%) | 3/40 (6.8%) |
| Relapse | Not separated | Not separated |
| Defervescence by 2 weeks | 76% | 76.7% |
| Adverse Events | 8.6% | 11.1% |
| Serious AE | 0 | 0 |
| Discontinuations | 0 | 0 |

**📑 Meta-Analysis–Ready Dataset**

Formatted for **RevMan/Stata entry**:

| **Outcome** | **Study** | **Intervention (n/N)** | **Control (n/N)** | **Effect Size** |
| --- | --- | --- | --- | --- |
| **Therapeutic Failure** | Didgar 2012 | 6/40 | 3/40 | OR = 2.14 (95% CI ~0.49–9.36) |
| **Cure** | Didgar 2012 | 34/40 | 37/40 | OR = 0.47 (95% CI ~0.11–2.03) |
| **Adverse Events** | Didgar 2012 | 3/40 | 4/40 | OR = 0.73 (95% CI ~0.16–3.26) |
| **Time to Defervescence** | Didgar 2012 | Mean 2 wks, 76% resolved | Mean 2 wks, 76.7% resolved | MD ~0 (no difference) |

Article 6

**1. Study Characteristics**

| **Item** | **Details** |
| --- | --- |
| Author(s) | Alikhani A, Heidarzadeh A |
| Year | 2007 |
| Journal | Journal of Isfahan Medical School |
| Location | Iran (Tonekabon, Guilan) |
| Age Range | 15–80 years |
| Mean Age | Similar across groups (not separately reported) |
| BMI | Not reported |
| Participant Status | Acute uncomplicated brucellosis |
| Study Design | Single-blind RCT, parallel |
| Sample Size | 78 randomized (38 ofloxacin, 40 doxycycline); 75 completed |
| Sex Distribution | Not reported explicitly |
| Entered vs Completed | 78 entered; 75 completed (96.1%) |
| Intervention Group | 38 (ofloxacin-based) |
| Control Group | 40 (doxycycline-based) |
| Duration of Study | 3 months treatment + 6 months follow-up |
| Dose of Intervention | Ofloxacin 200 mg PO q12h (month 1 with streptomycin, months 2–3 with rifampin) |
| Comparator Dose | Doxycycline 100 mg PO q12h (month 1 with streptomycin, months 2–3 with rifampin) |
| Journal Name | Journal of Isfahan Medical School |

**2. Participant Characteristics**

| **Item** | **Details** |
| --- | --- |
| Total Randomized | 78 |
| Total Analyzed | 75 |
| Population Description | Acute uncomplicated brucellosis |
| Diagnostic Criteria | Clinical + Wright test ≥1:160, 2ME ≥1:20 |
| Inclusion Criteria | Age ≥15, acute uncomplicated brucellosis |
| Exclusion Criteria | Neurobrucellosis, endocarditis, spondylitis, relapse, pregnancy, <15 years |
| Baseline Characteristics | Age 15–80, symptoms ≤3 months, no complications; groups similar |
| FQ Group | N=38 |
| Standard Group | N=40 |

**3. Intervention & Comparison**

| **Feature** | **FQ Group** | **Standard Group** |
| --- | --- | --- |
| Regimen | Streptomycin + Ofloxacin (month 1), Ofloxacin + Rifampin (months 2–3) | Streptomycin + Doxycycline (month 1), Doxycycline + Rifampin (months 2–3) |
| Drug 1 | Ofloxacin 200 mg q12h PO | Doxycycline 100 mg q12h PO |
| Drug 2 | Rifampin 600 mg q24h PO (months 2–3) | Rifampin 600 mg q24h PO (months 2–3) |
| Additional | Streptomycin 750–1000 mg IM daily × 1 month | Streptomycin 750–1000 mg IM daily × 1 month |
| Duration | 3 months | 3 months |
| Route | Oral + IM | Oral + IM |

**4. Outcomes**

**Efficacy**

| **Outcome** | **FQ Group (Ofloxacin)** | **Standard (Doxycycline)** |
| --- | --- | --- |
| Primary Cure | 38/38 (100%) | 37/37 (100%) |
| Relapse (6 mo f/u) | 0/38 | 0/37 |
| Therapeutic Failure | 0/38 | 0/37 |

**Safety**

| **Outcome** | **FQ Group (Ofloxacin)** | **Standard (Doxycycline)** |
| --- | --- | --- |
| Any AE | 5/38 (13.2%) | 18/37 (48.6%) |
| Common AEs | GI upset, nausea, epigastric pain, diarrhea, photosensitivity | Same but more frequent (notably photosensitivity) |
| SAE | None reported | None reported |
| Treatment Discontinuation | None | None |

**5. Risk of Bias (Cochrane RoB2)**

| **Domain** | **Judgment** |
| --- | --- |
| Randomization | Some concerns (block randomization mentioned, concealment unclear) |
| Deviations from intended interventions | Some concerns (single-blind only) |
| Missing outcome data | Low risk (96% completed) |
| Measurement of outcome | Some concerns (not blinded assessor) |
| Selection of reported result | Some concerns (no protocol preregistration) |

**6. Fluoroquinolone-Focused Summary**

- **Efficacy**: 100% cure, 0% relapse, 0% failure.
- **Safety**: 13.2% AE rate, mostly mild GI or skin reactions.
- **Comparison**: Significantly fewer AEs than doxycycline-based regimen (p<0.001).

**7. Comparison Table (Side-by-Side)**

| **Feature** | **Ofloxacin (FQ)** | **Doxycycline (Standard)** |
| --- | --- | --- |
| Cure | 100% | 100% |
| Relapse | 0% | 0% |
| AE Rate | 13.2% | 48.6% |
| Common AE | Mild GI, skin | GI, skin, photosensitivity |
| SAE | None | None |

**8. Meta-Analysis–Ready Dataset**

| **Outcome** | **OR / MD** | **95% CI** | **Notes** |
| --- | --- | --- | --- |
| Therapeutic Failure (0 vs 0) | Not estimable | — | Both 100% cure |
| Relapse | Not estimable | — | 0% in both groups |
| Any AE (5/38 vs 18/37) | OR = 0.14 | 95% CI: 0.04 – 0.44 | Favours ofloxacin |
| Serious AE | OR not estimable | — | None reported |
| Treatment Discontinuation | OR not estimable | — | None reported |

Article 7

**1. Study Characteristics**

| **Data Point** | **Details** |
| --- | --- |
| Author(s) | Agalar C, Usubutun S, Turkyilmaz R |
| Year | 1999 |
| Journal | Eur J Clin Microbiol Infect Dis |
| Location | Ankara, Turkey |
| Age Range | 15–65 (Group 1), 16–60 (Group 2) |
| Mean Age | 37.1 ± 15.8 (Doxy-Rif); 37.8 ± 13.9 (Cipro-Rif) |
| BMI | Not reported |
| Participants Status | Hospitalized brucellosis patients |
| Study Design | Open-label, randomized controlled trial |
| Sample Size | 40 total (20 per group) |
| Sex Distribution | Group 1: 13M/7F; Group 2: 8M/12F |
| Completed Study | All 40 completed |
| Intervention Group | 20 |
| Control Group | 20 |
| Duration of Study | Treatment 30–45 days; follow-up 12 months |
| Dose of Intervention | Ciprofloxacin 500 mg PO BID + Rifampicin 600 mg PO daily |
| Control | Doxycycline 100 mg PO BID + Rifampicin 600 mg PO daily |
| Journal | Eur J Clin Microbiol Infect Dis (Springer) |

**2. Participant Characteristics**

| **Data Point** | **Group 1 (Doxy-Rif)** | **Group 2 (Cipro-Rif)** |
| --- | --- | --- |
| Total Randomized | 20 | 20 |
| Total Analyzed | 20 | 20 |
| Population | Acute brucellosis |  |
| Diagnostic Criteria | Clinical + serology + blood culture |  |
| Inclusion/Exclusion | Excluded: <15y, seizures, recent antibiotics, pregnancy, antibiotic allergy, negative cultures |  |
| Mean Age | 37.1 ± 15.8 | 37.8 ± 13.9 |
| Age Range | 15–65 | 16–60 |
| Sex | 13M/7F | 8M/12F |
| Duration of Symptoms Pre-treatment | 36.5 ± 28 days | 24.4 ± 16.1 days |
| Complications | Arthritis (3), sacroileitis (1), orchitis (1) | Arthritis (3), sacroileitis (2) |

**3. Intervention & Comparison**

| Group | Regimen | Drug 1 | Dose & Frequency | Drug 2 | Dose & Frequency | Duration | Route |
| --- | --- | --- | --- | --- | --- | --- | --- |
| FQ Arm | Ciprofloxacin + Rifampicin | Ciprofloxacin (FQ) | 500 mg PO BID | Rifampicin | 600 mg PO daily | 30 days | Oral |
| Comparator | Doxycycline + Rifampicin | Doxycycline (Tetracycline) | 100 mg PO BID | Rifampicin | 600 mg PO daily | 45 days | Oral |

**4. Outcome Data**

| **Outcome** | **FQ Group (Cipro-Rif, n=20)** | **Comparator (Doxy-Rif, n=20)** |
| --- | --- | --- |
| Therapeutic Failure | 3/20 (15%) relapse | 2/20 (10%) relapse |
| Relapse Rate | 3/20 (15%) | 2/20 (10%) |
| Time to Defervescence | 2.78 ± 1.03 days | 3.85 ± 1.98 days |
| Adverse Events | 0/20 | 0/20 |
| Serious AEs | None | None |
| Discontinuations | None | None |

**5. Risk of Bias (Cochrane RoB 2)**

| **Domain** | **Judgment** |
| --- | --- |
| Randomization process | Low risk (randomization table) |
| Deviations from intended interventions | High risk (open-label, not blinded) |
| Missing outcome data | Low risk (all completed) |
| Measurement of outcome | Some concerns (clinical judgment may be biased, unblinded) |
| Selection of reported result | Some concerns (not pre-registered) |

**Fluoroquinolone-Specific Focus (Cipro-Rif Arm only)**

| **Outcome** | **Result** |
| --- | --- |
| Sample Size | 20 |
| Relapse | 3/20 (15%) |
| Therapeutic Success | 85% |
| Time to Defervescence | 2.78 ± 1.03 days |
| Adverse Events | None |

**Side-by-Side Comparison**

| **Outcome** | **Fluoroquinolone (Cipro-Rif)** | **Standard (Doxy-Rif)** |
| --- | --- | --- |
| n | 20 | 20 |
| Relapse | 15% (3/20) | 10% (2/20) |
| Treatment Success | 85% | 90% |
| Defervescence | 2.78 ± 1.03 d | 3.85 ± 1.98 d |
| Adverse Events | 0 | 0 |

**Meta-Analysis–Ready Dataset**

- **Therapeutic Failure (Relapse)**
  - FQ: 3/20
  - Comparator: 2/20
  - OR = 1.58 (95% CI: 0.24–10.3)
- **Time to Defervescence (Mean Difference)**
  - FQ: 2.78 ± 1.03 (n=20)
  - Comparator: 3.85 ± 1.98 (n=20)
  - MD = -1.07 days (95% CI: -2.04 to -0.10)
- **Adverse Events**
  - Both groups 0 → not estimable

Article 8

**1. Study Characteristics**

| **Item** | **Details** |
| --- | --- |
| Author(s) | Bayindir, Sonmez, Aladag, Buyukberber |
| Year | 2003 |
| Journal | Journal of Chemotherapy |
| Location | Malatya, Turkey |
| Study design | Prospective, randomized trial (parallel) |
| Population | Brucellar spondylitis |
| Sample size | 102 total |
| Groups | 5 arms: ST (20), SD (21), DR (20), OR (19), SDR (22) |
| Female/Male | 46 / 56 |
| Age range | 21–69 years |
| Mean age | 40.5 years overall |
| BMI | Not reported |
| Entered vs completed | 102 enrolled; 102 completed (all analyzed) |
| Duration of therapy | 45 days (all groups) |
| Follow-up | ≥ 6 months after treatment |
| Intervention (FQ arm) | Ofloxacin 200 mg PO q12h + Rifampicin 15 mg/kg PO qd, 45 days |
| Comparator regimens | ST: Streptomycin + Tetracycline; SD: Streptomycin + Doxycycline; DR: Doxycycline + Rifampicin; SDR: Streptomycin + Doxycycline + Rifampicin |

**2. Participant Characteristics**

| **Item** | **Details** |
| --- | --- |
| Total randomized | 102 |
| Total analyzed | 102 |
| Population description | Brucellar spondylitis |
| Diagnostic criteria | History of unpasteurized dairy, clinical symptoms, SAT ≥1:160, culture, CT/MRI spine |
| Inclusion | Adults with brucellar spondylitis, confirmed serology and imaging |
| Exclusion | Not explicitly listed |
| Baseline mean age (OR arm) | 37.1 years |
| Gender (OR arm) | 10M / 9F |
| Duration of symptoms before therapy (OR arm) | 9 weeks |
| Complications | Mostly diffuse spondylitis; some with abscesses (rare) |

**3. Intervention & Comparison Details**

| **Group** | **Drugs** | **Dose & Duration** | **Route** |
| --- | --- | --- | --- |
| OR (FQ) | Ofloxacin + Rifampicin | Ofloxacin 200 mg PO q12h × 45d; Rifampicin 15 mg/kg PO qd × 45d (max 900 mg) | Oral |
| ST | Streptomycin + Tetracycline | Streptomycin 1g IM × 15d + Tetracycline 500 mg PO q6h × 45d | IM + PO |
| SD | Streptomycin + Doxycycline | Streptomycin 1g IM × 15d + Doxycycline 100 mg PO q12h × 45d | IM + PO |
| DR | Doxycycline + Rifampicin | Doxycycline 100 mg PO q12h × 45d + Rifampicin 15 mg/kg PO qd × 45d | PO |
| SDR | Streptomycin + Doxycycline + Rifampicin | Streptomycin 1g IM × 15d + Doxycycline 100 mg PO q12h × 45d + Rifampicin 15 mg/kg PO qd × 45d | IM + PO |

**4. Outcome Data**

**Primary & Secondary Efficacy**

| **Outcome** | **OR (Ofloxacin+Rifampicin)** | **Comparators** |
| --- | --- | --- |
| Therapeutic failure | 5/19 (26%) | ST: 2/20 (10%), SD: 4/21 (19%), DR: 3/20 (15%), SDR: 0/22 (0%) |
| Relapse | 5/19 (26%) | ST: 0%, SD: 0%, DR: 2/20 (10%), SDR: 0% |
| Good response (cure) | 9/19 (47%) | ST: 90%, SD: 81%, DR: 75%, SDR: 100% |
| Time to defervescence | Not reported | – |
| Adverse events | GI upset most common (12–17% across groups), none required discontinuation | – |
| Serious adverse events | None reported | – |
| Discontinuations | 0 | – |

**5. Risk of Bias (Cochrane RoB 2)**

| **Domain** | **Judgment** |
| --- | --- |
| Randomization | Some concerns (randomization stated, concealment not detailed) |
| Deviations from intervention | Some concerns (not blinded) |
| Missing outcome data | Low (all completed) |
| Outcome measurement | Some concerns (non-blinded clinical outcomes) |
| Selective reporting | Some concerns (no protocol, but major outcomes reported) |

**Fluoroquinolone-Focused Results (OR arm only)**

| **Outcome** | **n/N (%)** |
| --- | --- |
| Therapeutic failure | 5/19 (26%) |
| Relapse | 5/19 (26%) |
| Good response | 9/19 (47%) |
| Adverse events | Mild GI complaints (not quantified separately) |
| Discontinuations | 0 |

**Comparison Table (FQ vs Best Comparator)**

| **Outcome** | **FQ (OR)** | **SDR (Best)** |
| --- | --- | --- |
| Therapeutic failure | 26% | 0% |
| Relapse | 26% | 0% |
| Good response | 47% | 100% |
| Adverse events | Mild GI only | Mild GI only |
| Discontinuations | 0 | 0 |

**Meta-Analysis–Ready Dataset**

| **Outcome** | **FQ Events/Total** | **Comparator Events/Total** | **OR [95% CI]** |
| --- | --- | --- | --- |
| Therapeutic failure | 5/19 | 2/20 (ST) | OR ≈ 3.2 (95% CI ~0.5–20) |
|  | 5/19 | 4/21 (SD) | OR ≈ 1.5 (95% CI ~0.3–7.2) |
|  | 5/19 | 3/20 (DR) | OR ≈ 2.0 (95% CI ~0.4–10) |
|  | 5/19 | 0/22 (SDR) | OR not estimable (favor SDR strongly) |
| Relapse | 5/19 | 0/20 (ST) | OR not estimable (favor ST) |
|  | 5/19 | 0/21 (SD) | OR not estimable (favor SD) |
|  | 5/19 | 2/20 (DR) | OR ≈ 3.4 (95% CI ~0.6–19) |
|  | 5/19 | 0/22 (SDR) | OR not estimable (favor SDR) |
| Cure (good response) | 9/19 | 22/22 (SDR) | OR ≈ 0.0 (favor SDR) |

✅ This gives you:

- **Structured tables** (study, participant, intervention, outcomes).
- **FQ-specific extraction** (OR arm).
- **Comparison table** (FQ vs SDR, best performing).
- **Meta-analysis–ready dataset** (ORs with CIs for RevMan/Stata).

Article 9

**1. Study Characteristics**

| **Data Point** | **Details** |
| --- | --- |
| Author(s) | Karabay O, Sencan I, Kayas D, Şahin I |
| Year | 2004 |
| Journal | BMC Infectious Diseases |
| Location | Düzce, Turkey |
| Age range | FQ: 18–60; Control: 24–61 |
| Mean/Median age | FQ: 35 (median); Control: 29 (median) |
| BMI | NR |
| Participant status | Hospitalized, acute brucellosis |
| Study design | RCT (open-label, parallel) |
| Sample size | Total randomized: 29 analyzed (FQ: 15, Control: 14); Randomized 34 → 5 excluded |
| Sex | FQ: 13 M, 2 F; Control: 11 M, 3 F |
| Completion | FQ: 15 entered, 14 completed (1 dropout); Control: 14 entered, 10 completed (4 dropout) |
| Duration of study | Recruitment Dec 1999 – Dec 2001; FU ≈ 150 days |
| Intervention dose | Ofloxacin 400 mg/day + Rifampicin 600 mg/day for 30 days |
| Control dose | Doxycycline 200 mg/day + Rifampicin 600 mg/day for 45 days |
| Journal | BMC Infectious Diseases |

**2. Participant Characteristics**

| **Data Point** | **Fluoroquinolone Arm (Ofloxacin + Rifampicin)** |
| --- | --- |
| Total randomized (N) | 15 |
| Total analyzed (N) | 14 (1 dropout) |
| Population | Acute brucellosis |
| Diagnostic criteria | Clinical symptoms + SAT ≥1:160 and/or positive culture |
| Inclusion | Age ≥15, no pregnancy, no seizure history |
| Exclusion | Pregnancy, seizure, age <15 |
| Mean/Median age | 35 (18–60) |
| Gender | 13 M / 2 F |
| Complications | 1 sacroileitis, 1 orchitis |
| Duration of symptoms before tx | NR |

**3. Intervention & Comparison Details**

| **Feature** | **Fluoroquinolone (FQ)** | **Control (Standard)** |
| --- | --- | --- |
| Regimen name | Ofloxacin + Rifampicin | Doxycycline + Rifampicin |
| Drug 1 | Ofloxacin (FQ) 400 mg OD PO | Doxycycline 100 mg BID PO |
| Drug 2 | Rifampicin 600 mg OD PO | Rifampicin 600 mg OD PO |
| Duration | 30 days | 45 days |
| Route | Oral | Oral |

**4. Outcomes**

| **Outcome** | **FQ Group (Ofloxacin + Rifampicin)** | **Control (Doxycycline + Rifampicin)** |
| --- | --- | --- |
| **Therapeutic failure** (relapse + no response) | Relapse: 2/15 | Relapse: 2/14 |
| **Relapse rate** | 2/15 (13.3%) | 2/14 (14.3%) |
| **Time to defervescence** | 74 ± 30 hrs | 106 ± 26 hrs |
| **Any adverse event** | Nausea/vomiting: 1; Diarrhea: 2 | Nausea/vomiting: 3; Diarrhea: 1 |
| **SAE** | None reported | None |
| **Treatment discontinuation** | 1 dropout (refused drugs) | 4 dropouts (felt well, stopped) |

**5. Risk of Bias (Cochrane RoB 2)**

| **Domain** | **Judgment** |
| --- | --- |
| Randomization process | Some concerns (method NR) |
| Deviations from intended interventions | High (open-label, no blinding) |
| Missing outcome data | Low (small dropout, balanced) |
| Measurement of outcome | Some concerns (subjective outcomes, not blinded) |
| Selection of reported result | Low (protocol outcomes reported) |

**Fluoroquinolone-Specific Results (extracted)**

| **Endpoint** | **FQ Result** |
| --- | --- |
| Cure rate | 13/15 (86.7%) |
| Relapse | 2/15 |
| Time to defervescence | 74 ± 30 hrs |
| Adverse events | Nausea/vomiting: 1, Diarrhea: 2 |
| Discontinuation | 1/15 |
| Follow-up | 156 ± 15 days |

**Comparison Table (FQ vs Control)**

| **Outcome** | **Ofloxacin + Rifampicin** | **Doxycycline + Rifampicin** |
| --- | --- | --- |
| N (analyzed) | 15 | 14 |
| Relapse | 2 (13.3%) | 2 (14.3%) |
| Time to defervescence | 74 ± 30 hrs | 106 ± 26 hrs |
| AE: nausea/vomiting | 1 (6.7%) | 3 (21.4%) |
| AE: diarrhea | 2 (13.3%) | 1 (7.1%) |
| SAE | None | None |
| Dropout | 1 | 4 |

**Meta-Analysis–Ready Dataset (RevMan/Stata format)**

**Dichotomous outcomes (Relapse, Adverse Events)**

| **Outcome** | **FQ n/N** | **Control n/N** | **OR (95% CI)** |
| --- | --- | --- | --- |
| Relapse | 2/15 | 2/14 | OR = 0.93 (95% CI 0.12–7.40) |
| Any AE (nausea/diarrhea) | 3/15 | 4/14 | OR = 0.65 (95% CI 0.13–3.17) |
| Dropout | 1/15 | 4/14 | OR = 0.21 (95% CI 0.02–2.11) |

**Continuous outcomes (Time to defervescence)**

| **Outcome** | **FQ Mean (SD)** | **Control Mean (SD)** | **MD (95% CI)** |
| --- | --- | --- | --- |
| Time to defervescence (hours) | 74 (30) | 106 (26) | MD = –32 hrs (95% CI –51 to –13), p=0.016 |
