# Supplementary material for: Efficacy and safety of fluoroquinolones in the treatment of human brucellosis: A systematic review and meta-analysis
Source: PLoS Negl Trop Dis. 2026 Oct 23;20(10):e0013822. doi: 10.1371/journal.pntd.0013822 (PMC13722316; doi:10.1371/journal.pntd.0013822)
Supplement: Supplementary file 6 [file pntd.0013822.s005.docx]

sTable 4: GRADE results

| **Summary of findings:** | | | | |
| --- | --- | --- | --- | --- |
| **Patient or population:** Patients with brucellosis **Setting:** Randomized controlled trials  **Intervention:** Fluoroquinolone-based regimens **Comparison:** Standard therapy (doxycycline ± rifampicin/streptomycin) | | | | |
| Outcomes | Relative effect (95% CI) | № of participants (studies) | Certainty of the evidence (GRADE) | Comments |
|  |  |  |  |  |
| Therapeutic failure | **2.11 1.26-3.51** | 524 participants (8 studies) | ⨁⨁⨁◯ moderate^1a^ | Fluoroquinolone-based regimens significantly increase risk of treatment failure compared with standard therapy. |
| Relapse | **1.88 0.94-3.74** | 444 participants (7 studies) | ⨁⨁◯◯ low^1ac^ | Fluoroquinolone-based regimens may be associated with a higher risk of relapse compared with standard therapy; however, the confidence interval includes no difference. |
| Adverse events | **0.62 0.29-1.33** | 552 participants (7 studies) | ⨁⨁◯◯ Low¹ᵇᶜ | Fluoroquinolone-containing regimens may result in little or no difference in adverse events compared with non-fluoroquinolone regimens. |
| Time to defervescence | **MD=1.20 0.66-1.74** | 140 participants (4 studies) | ⨁⨁⨁◯ moderate^1a^ | Fluoroquinolone-based regimens associated with significantly longer time to fever resolution. |
| ***The risk in the intervention group** (and its 95% confidence interval) is based on the assumed risk in the comparison group and the **relative effect** of the intervention (and its 95% CI).  **CI:** confidence interval; RR**:** risk ratio, MD: mean differences | | | | |
| **GRADE Working Group grades of evidence** **High certainty:** Very confident in effect estimate  **Moderate certainty:** True effect likely close to estimate  **Low certainty:** Effect may be substantially different  **Very low certainty:** Very little confidence in estimate | | | | |

#### Explanations

a) some concerns regarding risk of bias and study limitations

b) Downgraded for inconsistency due to moderate statistical heterogeneity across studies (I² = 52%).

c) Downgraded for imprecision because the 95% confidence interval included both a potentially important reduction and increase in adverse events.
