# Supplementary material for: Efficacy and safety of fluoroquinolones in the treatment of human brucellosis: A systematic review and meta-analysis
Source: PLoS Negl Trop Dis. 2026 Oct 23;20(10):e0013822. doi: 10.1371/journal.pntd.0013822 (PMC13722316; doi:10.1371/journal.pntd.0013822)
Supplement: Supplementary file 8 [file pntd.0013822.s007.docx]

PLOS Neglected Tropical Diseases

Efficacy and Safety of Fluoroquinolones in the Treatment of Human Brucellosis: A Systematic Review and Meta-Analysis

--Manuscript Draft--

| Manuscript Number: | PNTD-D-25-02141 |
| --- | --- |
| Full Title: | Efficacy and Safety of Fluoroquinolones in the Treatment of Human Brucellosis: A Systematic Review and Meta-Analysis |
| Short Title: | Review of Fluoroquinolones for Brucellosis |
| Article Type: | Research Article |
| Keywords: | Brucellosis; Zoonoses; Fluoroquinolones; Therapeutic failure; Treatment outcome |
| Abstract: | Introduction  Brucellosis is major zoonotic infection worldwide, accounting for an estimated 2.1 million cases, with the highest burden reported in the mediterranean region. The use of fluoroquinolones in the treatment of this disease is controversial. In this meta-analysis, we aimed to investigate the effects of fluoroquinolones in the treatment of brucellosis.  Methods  This systematic review and meta-analysis was prospectively registered in PROSPERO (CRD420251142019) and carried out following the PRISMA 2020 guidelines. The authors conducted an extensive literature search in PubMed, Cochrane Library, Web of Science, and Scopus through August 2, 2025, using a set of predefined terms related to fluoroquinolones and treatment outcomes. Only randomized clinical trials in English or Persian that focused on the use of fluoroquinolone-based regimens for the treatment of acute or chronic brucellosis were considered for the review. Combined risk ratios (RR), odds ratios (OR), and mean differences (MD) were estimated using fixed-or random-effects models. Heterogeneity between studies was measured using the I2 statistic. Publication bias was assessed using funnel plots, and Egger and Begg tests were applied for this purpose.  Results  In this meta-analysis, the risk of treatment failure was approximately 2.2 times higher with fluoroquinolone-containing regimens than with standard therapy (RR = 2.23; 95% CI: 1.39–3.57; P = 0.001), with no heterogeneity observed between studies (I2 = 0%). For relapse, there was no statistically significant difference between fluoroquinolone and standard therapy (RR = 1.63; 95% CI: 0.90–2.97; P ≈ 0.11). Regarding adverse events, the random-effects model showed no significant difference, although the fixed-effects model indicated a slight reduction in favor of fluoroquinolones (OR ≈ 0.53; 95% CI: 0.31–0.89). The time to defervescence was, on average, approximately 0.9 days longer in the fluoroquinolone group (P < 0.001). Subgroup analyses showed that these adverse outcomes were mainly associated with ciprofloxacin-based regimens, whereas outcomes related to ofloxacin were not significantly different from those of standard therapy.  Conclusion  Fluoroquinolones should not be used as first-line therapy for brucellosis, although ofloxacin-based regimens may be considered in specific cases where standard agents are contraindicated. |
| Additional Information: |  |
| Question | Response |
| Government Employee | No - No authors are employees of the U.S. government. |

*Powered by Editorial Manager® and ProduXion Manager® from Aries Systems Corporation*

| Are you or any of the contributing authors an employee of the United States government?  Manuscripts authored by one or more US Government employees are not copyrighted, but are licensed under a [CC0](https://creativecommons.org/about/cc0) [Public Domain Dedication](https://creativecommons.org/about/cc0), which allows unlimited distribution and reuse of the article for any lawful purpose. This is a legal requirement for US Government employees.  This will be typeset if the manuscript is accepted for publication. |  |
| --- | --- |
| Financial Disclosure  Enter a financial disclosure statement that describes the sources of funding for the work included in this submission. Review the [submission guidelines](http://journals.plos.org/plosntds/s/submission-guidelines#loc-financial-disclosure-statement) for detailed requirements. View published research articles from [PLOS Neglected Tropical](https://journals.plos.org/plosntds/) [Diseases](https://journals.plos.org/plosntds/) for specific examples.  This statement is required for submission and will appear in the published article if the submission is accepted. Please make sure it is accurate. | The author(s) received no specific funding for this work. |
| Funded studies  Enter a statement with the following details:   - Initials of the authors who received each award - Grant numbers awarded to each author - The full name of each funder - URL of each funder website - Did the sponsors or funders play any role in the study design, data collection and analysis, decision to publish, or preparation of the manuscript? |  |
| Did you receive funding for this work? |  |
| Competing Interests | The authors have declared that no competing interests exist. |

*Powered by Editorial Manager® and ProduXion Manager® from Aries Systems Corporation*

| On behalf of all authors, disclose any competing interests that could be perceived to bias this work.  This statement will be typeset if the manuscript is accepted for publication.  Review the instructions link below and PLOS NTDs’ [competing interests](https://journals.plos.org/plosntds/s/competing-interests#loc-what-represents-a-competing-interest) policy to determine what information must be disclosed at submission. |  |
| --- | --- |
| Data Availability  Provide a Data Availability Statement in the box below. This statement should detail where the data used in this submission can be accessed. This statement will be typeset if the manuscript is accepted for publication.  Before publication, authors are required to make all data underlying their findings fully available, without restriction. Review our [PLOS Data Policy](https://journals.plos.org/plosntds/s/data-availability) page for detailed information on this policy. Instructions for writing your Data Availability statement can be accessed via the Instructions link below. | Data Availability  Provide a Data Availability Statement in the box below. This statement should detail where the data used in this submission can be accessed. This statement will be typeset if the manuscript is accepted for publication.  Before publication, authors are required to make all data underlying their findings fully available, without restriction. Review our PLOS Data Policy page for detailed information on this policy. Instructions for writing your Data Availability statement can be accessed via the Instructions link below. |

*Powered by Editorial Manager® and ProduXion Manager® from Aries Systems Corporation*

<https://www2.cloud.editorialmanager.com/pntd/download.aspx?id=1515605&guid=fc304a16-85d8-48d5-8e85-509cb2ab9a5e&scheme=1>Manuscript	[Click here to access/download;Manuscript;FQ on brucellosis manuscript.docx](https://www2.cloud.editorialmanager.com/pntd/download.aspx?id=1515605&guid=fc304a16-85d8-48d5-8e85-509cb2ab9a5e&scheme=1)

**Efficacy and Safety of Fluoroquinolones in the Treatment of Human Brucellosis: A Systematic Review and Meta-Analysis**

## Mohsen Mohammadi-Sartang^1*^, Nastaran Rafieipour^2*^, Shahla Afrasiabian^3^, Roya Mahmoudi^4^, Karo Servatyar^5^

1- **Department of Clinical Nutrition, School of Nutrition and Food Sciences, Shiraz University**

**of Medical Sciences, Shiraz, Iran**

2- **Islamic Azad University, Rasht Branch, Rasht, Iran**
3- **Department of Infectious Diseases, Faculty of Medicine, Kurdistan University of Medical**

**Sciences, Sanandaj, Iran**

4- **Nutrition Research Center, School of Nutrition and Food Sciences, Tabriz University of**

**Medical Sciences, Tabriz, Iran**

5- **Student Research Committee, Kurdistan University of Medical Sciences, Sanandaj, Iran Corresponding Author: Infectious Disease Residency, Email:** [**Servatyarikaro@yahoo.com,**](mailto:Servatyarikaro@yahoo.com)

**Phone: +989186967936, Fax: +988733664643**

**(* co-first authorship)**

## Running title: Review of Fluoroquinolones for Brucellosis

**Abstract**

**Introduction:** Brucellosis is major zoonotic infection worldwide, accounting for an estimated 2.1 million cases, with the highest burden reported in the mediterranean region. The use of fluoroquinolones in the treatment of this disease is controversial. In this meta-analysis, we aimed to investigate the effects of fluoroquinolones in the treatment of brucellosis.

**Methods:** This systematic review and meta-analysis was prospectively registered in PROSPERO (CRD420251142019) and carried out following the PRISMA 2020 guidelines. The authors conducted an extensive literature search in PubMed, Cochrane Library, Web of Science, and Scopus through August 2, 2025, using a set of predefined terms related to fluoroquinolones and treatment outcomes. Only randomized clinical trials in English or Persian that focused on the use of fluoroquinolone-based regimens for the treatment of acute or chronic brucellosis were considered for the review. Combined risk ratios (RR), odds ratios (OR), and mean differences (MD) were estimated using fixed- or random-effects models. Heterogeneity between studies was measured using the I² statistic. Publication bias was assessed using funnel plots, and Egger and Begg tests were applied for this purpose.

**Results:** In this meta-analysis, the risk of treatment failure was approximately 2.2 times higher with fluoroquinolone-containing regimens than with standard therapy (RR = 2.23; 95% CI: 1.39–3.57; P = 0.001), with no heterogeneity observed between studies (I² = 0%). For relapse, there was no statistically significant difference between fluoroquinolone and standard therapy (RR = 1.63; 95% CI: 0.90–2.97; P ≈ 0.11). Regarding adverse events, the random-effects model showed no significant difference, although the fixed-effects model indicated a slight reduction in favor of fluoroquinolones (OR ≈ 0.53; 95% CI: 0.31–0.89). The time to defervescence was, on average, approximately 0.9 days longer in the fluoroquinolone group (P < 0.001). Subgroup analyses showed that these adverse outcomes were mainly associated with ciprofloxacin-based regimens, whereas outcomes related to ofloxacin were not significantly different from those of standard therapy.

**Conclusion:** Fluoroquinolones should not be used as first-line therapy for brucellosis, although ofloxacin-based regimens may be considered in specific cases where standard agents are contraindicated.

**Keywords**: Brucellosis, Zoonoses, Fluoroquinolones, Therapeutic failure, Treatment outcome

## Introduction

Brucellosis, also known as Malta fever, is a serious communicable zoonosis of great importance that is prevalent in various regions of the world, particularly in the Middle East and Iran [1]. Humans are infected by bacteria of the genus Brucella through contact with contaminated animals, contact with their body fluids, or intake of unpasteurized milk and its derivatives, and develop a pervasive systemic infection [2].

Between 2015 and 2016, bovine brucellosis prevalence in Kuwait and Jordan ranged between 7.2% and 18.1%. In comparison, during 2012–2014, the prevalence was much lower—around 3% to 3.1%—in Iraq and Iran. In Iran, the prevalence of caprine brucellosis due to Brucella melitensis was estimated at 3.5% in 2014. At present, the test-and-slaughter control strategy is mainly used in countries where disease prevalence remains below 2%. [3].

Effective control of brucellosis has invariably provided a significant challenge to the field of medicine, in part due to Brucella's ability to endure within host cells, such as macrophages and reticuloendothelial cells, and to promote latent infection reservoirs. Their intracellular environment impairs the efficacy of many conventional antibiotics to reach an effective concentration, and the resultant occurrence of relapse following successful initial treatment is a significant concern (4). Brucella invades host cells, suppresses lysosomal fusion, and moves to the endoplasmic reticulum to replicate. Its survival within cells relies on the BvrS/BvrR regulatory system, VirB type IV secretion machinery, and a complete lipopolysaccharide (LPS) layer [4].

In a meta-analysis conducted by Big et al., the efficacy of fluoroquinolones for the treatment of Brucella spp. was reported, with low resistance levels (1.6–4.6%), and a recommendation for a relatively safe option in cases of relapse or drug-related complications. Because of their intracellular activities, low toxicity to the kidneys, and relapse prevention potential, fluoroquinolones are a suitable choice for patients who develop complications from other agents [5].

In contrast, a domestic systematic review titled "Antibiotic susceptibility of Brucella spp. isolated from human specimens" showed alarming results. Although the first focus of the study was on

overseas data, the study results show that there are strains of Brucella whose susceptibility to quinolones, such as ciprofloxacin, is reduced [6].

While the usual therapy for brucellosis involves regimens of doxycycline and rifampin or doxycycline and streptomycin [7], fluoroquinolones have been suggested as an alternative or adjunctive mode of treatment in selected cases. This is because of various beneficial factors, including adequate oral absorption, acceptable tolerability, and intracellular penetration [8].

A comprehensive comparison of laboratory tests and clinical trials revealed that there is currently insufficient evidence to justify the approval of quinolones as a first-line therapeutic agent for human brucellosis [9]. Newer agents of the fluoroquinolone group possess enhanced activity against gram-negative bacilli and selected gram-positive organisms, such as Streptococcus pneumoniae, Staphylococcus aureus, and selected anaerobic organisms. These agents possess a longer half-life, are administered once daily at a fixed dose, and selected agents are eliminated through non-renal clearance. Clinical trials have established positive efficacy for a range of infections relative to standard treatment regimens; however, a direct comparison of various fluoroquinolone agents has not yet been conducted [10].

This research combines current evidence regarding the debated role of fluoroquinolones in the treatment of brucellosis, with the aim to assess their effectiveness, describe their benefits and drawbacks, and suggest guidance for upcoming clinical trials.

**Methods**

## Search Strategy

The systematic review and meta-analysis is registered with PROSPERO (CRD420251142019), and it followed the PRISMA 2020 protocol. The literature search for the systematic review and meta-analysis included the databases of PubMed, Scopus, Web of Science, and the Cochrane Library up to August 2, 2025. The terms used during the search incorporated both the MeSH terms and free-text terms that described both Brucellosis and fluoroquinolones, including: “brucellosis”, ”Brucell*”, “Malta fever”, ”Gibraltar Fever”, “undulant fever”, ”rock fever”, “Cyprus fever”, ”fluoroquinolone,” “ciprofloxacin,” “ofloxacin,” “levofloxacin,” “moxifloxacin,” “treatment

failure,” “relapse”,” “Treatment Outcome”, “therapeutics”, “therapy”,” “clinical trial”, ”single blinded method”,” “double blinded method”, ”comparative study”, “intervention”, “randomized”, “RCT”, “parallel,” and “ Only randomized clinical trials, in English or Persian, which focused on the use of fluoroquinolone-based regimens in the treatment of acute or chronic brucellosis, were considered for the review. All candidate studies were published in English and Persian. The bibliographic databases of the included studies and related reviews were also searched manually.

## Eligibility Criteria

Studies that fulfilled the following criteria were considered for inclusion.

1. Design: Randomized controlled trials (RCTs) or prospective comparative studies.
2. Population: Patients with brucellosis diagnosed using clinical, serological, or microbiological criteria.
3. Intervention: Treatment with a fluoroquinolone-based regimen, either alone or in combination with other agents.
4. Comparators: standard recommended regimens (e.g., doxycycline + rifampicin, doxycycline + streptomycin).
5. Outcomes: therapeutic failure, relapse, adverse events, and time to defervescence.

## Outcome definitions:

- Therapeutic failure: Lack of clinical improvement, persistence, or worsening of symptoms during treatment.
- Relapse: recurrence of symptoms or laboratory-confirmed brucellosis following the completion of therapy and initial cure.
- Adverse events: any reported drug-related side effects, particularly those leading to discontinuation or dose adjustment.
- Time to defervescence :number of days from treatment initiation to the sustained resolution of fever.

Studies were excluded if they were case series, retrospective cohorts, reviews, or if insufficient data were available to calculate effect sizes.

## Data Extraction

Two reviewers independently extracted data from each eligible study using a standardized data collection form. The extracted information included study characteristics (first author, publication year, country, and study design), demographic details (sample size, mean age, and sex distribution), intervention regimens (drug combinations, dosage, and treatment duration), comparator regimens, follow-up period, and all predefined outcomes of interest. When outcomes were not directly reported, effect sizes (e.g., means, standard deviations, or event counts) were derived from the available data or estimated using standard formulas. Discrepancies were resolved by consensus.

## Quality Assessment

**Assessing risk of bias (RoB-2 and ROBINS-I):** The risk of bias of randomized trials was evaluated using the Cochrane RoB 2 (RoB-2), which consists of five domains: (1) risk of bias due to the randomization procedure, (2) risk of bias due to deviations from the intended interventions,

(3) risk of bias due to incomplete outcome data, (4) risk of bias due to the measurement of the outcome, and (5) risk of bias due to the selection of the result reported. The risk of each domain could be categorized into “low risk of bias', “some concerns,' and “high risk of bias'. An overall risk of bias judgment could be assigned to each trial.

**Certainty of evidence (GRADE):** The certainty of the evidence for each of the outcomes was also assessed using the GRADE system, which takes into account five factors: risk of bias, inconsistency, indirectness, imprecision, and publication bias. The evidence is graded as high, moderate, low, and very low.

## Statistical Analysis

Meta-analyses were performed using Comprehensive Meta-Analysis (CMA) software, version 3.0. For dichotomous outcomes (therapeutic failure, relapse, and adverse events), risk ratios (RRs) with 95% confidence intervals (CIs) were calculated. For continuous outcomes (time to defervescence), mean differences (MDs) with 95% confidence intervals (CIs) were used. Both fixed-effect and random-effects (DerSimonian–Laird) models were applied, with random-effects estimates

reported as primary results. Heterogeneity was evaluated using Cochran’s Q-test and the I² measure, and the threshold for substantial heterogeneity was I²>50%.

Predefined subgroup analyses were performed according to the regimen type (ciprofloxacin-based, ofloxacin-based, or other fluoroquinolone regimens), treatment duration (short ≤6 weeks, standard 6–8 weeks, long ≥12 weeks), patient population (acute vs complicated), geographic location (Iran vs Turkey vs other countries), sample size (<50 vs ≥50), and follow-up duration (<6 vs ≥6 months). Sensitivity analyses were conducted by sequentially removing the individual studies.

Publication bias was assessed using funnel plots, Begg’s rank correlation, Egger’s regression test, and calculation of the classic and Orwin’s fail-safe N. Trim-and-fill analysis was also performed to estimate the potential impact of unpublished studies.

All statistical tests were two-tailed, and a p-value<0.05 was considered statistically significant.

## Fig1. PRISMA flow diagram of study selection

**Results**

The initial literature search yielded 231 articles that were screened. After the removal of 54 duplicates based on title and abstract screening, 134 irrelevant articles were excluded from the initial review. An additional 21 articles (7 case reports and 14 reviews) were excluded, resulting in the final inclusion of 12 clinical trials [11-22]. The selection process is illustrated in Figure 1.

Table 1 summarizes the general characteristics of the included studies. Twelve studies conducted between 1990 and 2014 across several countries, predominantly Iran and Turkey, with additional studies from Israel and Albania, were included. The sample sizes varied substantially, ranging from 10 to 178 participants (total: 816 patients). The mean age of the enrolled patients was generally in the third to fourth decade of life, with most studies including both men and women. Overall, there was a balanced sex distribution across the intervention and control groups, with no major sex-related imbalances reported. All included trials evaluated adult patients with brucellosis, most commonly acute uncomplicated disease, although some studies also enrolled patients with osteoarticular involvement or complicated forms, such as brucellar spondylitis. The intervention arms predominantly consisted of fluoroquinolone-based combinations, mainly ciprofloxacin or ofloxacin (administered orally in standard doses), combined with rifampicin or doxycycline. Control groups typically received standard regimens, most frequently doxycycline plus rifampicin or doxycycline combined with streptomycin. Treatment durations ranged from short courses of 6 weeks or 45 days to longer regimens of 3 months, depending on the trial design and patient population. Most of the included studies employed randomized parallel-group controlled trial designs, with a few prospective observational comparative studies. In summary, the included trials represent a relatively young adult population with acute brucellosis, with balanced demographic characteristics between groups, and investigated diverse fluoroquinolone-based regimens compared to conventional standard-of-care therapies over variable treatment durations.

**Therapeutic Failure**: In a pooled analysis of 10 studies, fluoroquinolone-based regimens were associated with a significantly higher risk of therapeutic failure than standard regimens. The overall risk ratio (RR) was 2.23 (95% CI: 1.39–3.57, p = 0.001), indicating that patients treated with fluoroquinolone-based combinations had more than twice the risk of treatment failure. Both fixed-effect and random-effects models yielded identical estimates, consistent with the absence of heterogeneity across studies (Q = 4.72, df = 9, p = 0.86; I² = 0%).

Sensitivity analysis, done using the sequential exclusion of each study, supported the robustness of these conclusions The pooled risk ratio consistently remained significant and in the same direction, with estimates ranging from RR 2.01 to 2.38 across all leave-one-out scenarios. No single study disproportionately influenced the overall effect size

**Relapse:** Ten studies reported relapse outcomes. The pooled analysis demonstrated no statistically significant difference in relapse risk between fluoroquinolone-based regimens and standard therapy (RR 1.63, 95% CI: 0.90–2.97, p = 0.108). Both fixed-effect and random-effects models produced identical estimates. Heterogeneity across studies was low (Q = 6.13, p = 0.73; I² = 0%), suggesting consistency of findings.

Sensitivity analysis, performed by sequentially omitting each study, did not materially alter the pooled estimates. The risk ratio remained above unity in all leave-one-out scenarios, ranging from

1.40 to 1.97, but consistently failed to reach statistical significance. This indicates that the results are robust and not driven by any single study.

**Adverse Events:** Eight studies reported data on adverse events. The pooled analysis under the random-effects model demonstrated no significant difference in the risk of adverse events between fluoroquinolone-based regimens and standard therapy (OR 0.57, 95% CI: 0.25–1.34, p = 0.201). In contrast, the fixed-effects model suggested a significantly lower risk with fluoroquinolone regimens (OR 0.53, 95% CI: 0.31–0.89, p = 0.017).

Moderate heterogeneity was observed among the studies (Q = 15.95, p = 0.026; I² = 56%), which likely explains the discrepancy between the fixed- and random-effects estimates. Sensitivity analysis, performed by excluding each study sequentially, showed that the direction and magnitude of the effect remained stable, with no single study exerting undue influence on the overall result.

**Time to Defervescence:** Five studies assessed the time to defervescence. The pooled analysis demonstrated that patients receiving fluoroquinolone-based regimens had a significantly longer time to fever resolution than those treated with standard therapy. The mean difference was 0.92 days (95% CI: 0.47–1.37, p < 0.001). Both fixed- and random-effects models yielded identical results.

No evidence of heterogeneity was detected (Q = 3.46, p = 0.48; I² = 0%), suggesting consistency across included trials. Sensitivity analysis, excluding each study sequentially, confirmed the robustness of the findings, with the mean difference remaining statistically significant in all scenarios.

## Subgroup Analyses (Table 2)

**Therapeutic Failure:** Subgroup analyses showed that the risk of therapeutic failure with fluoroquinolone-based regimens was consistently elevated in most subgroups. Both short (≤6 weeks) and longer (>6 weeks) treatment durations were associated with increased failure, with the effect reaching statistical significance in the latter (RR 2.48, 95% CI not shown, p = 0.001). Ciprofloxacin-based regimens were significantly inferior (RR 2.59, p = 0.001), whereas ofloxacin-based regimens did not differ from standard therapy (RR 1.35, p = 0.54). By geographic region, studies from Iran demonstrated a significant increase in therapeutic failure (RR 2.75, p = 0.001), whereas those from Turkey and other countries did not. The effect was particularly pronounced in trials with larger sample sizes (≥50 patients, RR 2.79, p = 0.02) and longer follow-up durations (≥6 months, RR 2.24, p = 0.004).

**Relapse:** No subgroup reached statistical significance, although trends were consistent with a higher relapse risk under fluoroquinolone regimens. Ciprofloxacin-based regimens showed a non-significant increase (RR 1.78, p = 0.1) with substantial heterogeneity (I² = 75%), whereas

ofloxacin-based regimens had no significant effect. A significant association was observed only in the “other countries” subgroup (RR 2.40, p = 0.03), whereas studies from Iran and Turkey did not demonstrate significant differences.

**Adverse Events**: Subgroup analyses of adverse events showed mixed results. Overall, there was no increased risk compared with standard therapy. However, ofloxacin-based regimens were associated with a significantly lower risk of adverse events (RR 0.24, p < 0.001), whereas ciprofloxacin-based regimens showed no difference (RR 0.97, p = 0.9). Considerable heterogeneity was noted in several subgroups (I² up to 85%).

**Time to Defervescence:** For results for time to defervescence were heterogeneous. Ciprofloxacin-based regimens appeared less effective (RR 0.63, p = 0.09), whereas ofloxacin-based regimens showed significantly faster defervescence (RR 1.25, p < 0.001). Subgroup analyses by country revealed a significant benefit in studies from Turkey (RR 1.20, p < 0.001) but no consistent effects in other regions.

**Publication Bias:** There are no obvious asymmetries revealed through the visual inspection of the funnel plots. The Begg’s rank correlation test and Egger’s regression tests for publication bias were not statistically significant for any of the outcomes. For therapeutic failure, Begg’s test yielded τ = –0.22 (p = 0.37) and Egger’s test an intercept of –0.26 (95% CI: –1.79 to 1.25, p = 0.69). Similar non-significant results were observed for relapse (Begg’s τ = 0.17, p = 0.47; Egger’s intercept = 1.04, p = 0.37), adverse events (Begg’s τ = 0.10, p = 0.71; Egger’s intercept = 1.37, p

= 0.42), and time to defervescence (Begg’s τ = 0.10, p = 0.80; Egger’s intercept = 1.63, p = 0.43).

The fail-safe N was relatively small across outcomes (10 for therapeutic failure and relapse, 8 for adverse events, and 5 for time to defervescence), suggesting that only a limited number of unpublished null studies are required to potentially overturn the findings. Although formal statistical tests did not indicate publication bias, the modest fail-safe N values highlight the need for cautious interpretation (Table 3).

## GRADE Assessment

Using the GRADE approach, the certainty of the evidence varied across outcomes. The evidence for therapeutic failure was rated as moderate, primarily downgraded due to concerns regarding the risk of bias and potential publication bias, but was supported by consistent findings across studies. The certainty of evidence for relapse was judged to be low, as the estimates were imprecise with wide confidence intervals and a limited number of events, despite the moderate consistency. For adverse events, the certainty was rated as moderate, with downgrading due to small sample sizes and imprecision, although the overall risk of bias was low. The evidence for time to defervescence was rated as moderate, with concerns only about the risk of bias, while heterogeneity and imprecision were minimal.

Overall, the quality of evidence ranged from low to moderate, suggesting that while FQ-based regimens may be associated with a higher risk of treatment failure than standard regimens, the strength of evidence supporting differences in relapse and adverse events remains limited.

## Discussion

This systematic review and meta-analysis included 12 randomized clinical trials (RCTs) that evaluated the effects of fluoroquinolone-based regimens on brucellosis treatment. The main results showed that the risk of therapeutic failure in the fluoroquinolone group was approximately 2.2 times higher than that in the standard treatment group (RR≈2.23). There was no significant difference in relapse compared with conventional treatment. In the analysis of adverse events, the random-effects model did not show a difference, although the fixed-effects model suggested a slight reduction in the incidence of complications in the fluoroquinolone group. The time to defervescence was also estimated to be approximately 0.9 days longer on average than that in the control group. Subgroup analyses indicated that these adverse events were mainly associated with ciprofloxacin-based regimens, whereas the results for ofloxacin did not differ significantly from those of the standard treatment group. From a methodological perspective, the very low

heterogeneity between studies, robustness of the results in sensitivity analyses, and lack of evidence of publication bias are the main strengths of this meta-analysis. However, the limitations of this review include the relatively small overall sample size, the greater geographic concentration of studies in Iran and Turkey, and the limited diversity in the types of fluoroquinolones studied. In addition, the relatively low fail-safe N value suggests that the inclusion of a small number of new negative studies could have influenced the overall results.

The relative ineffectiveness of fluoroquinolones against brucellosis is due to both the pharmacodynamic properties of the antibiotics and multiple bacterial resistance mechanisms.

They exert their action by inhibiting DNA gyrase (GyrA/GyrB) and topoisomerase, which are induced by mutations in the quinolone resistance–determining region (QRDR) of GyrA and/or GyrB, disrupting drug binding to target enzymes and generating elevated minimum inhibitory concentrations (MICs).

In an in vitro study, Rodriguez-Tarazona et al. showed that exposure to ciprofloxacin (CIP) and levofloxacin (LEV) induced mutations in gyrA, such as Ala87Val, and increased resistance [23].

The lack of adequate drug permeation to macrophages and other sub-tissue regions (such as foamy macrophages) also impedes bacterial clearance. Decreased perfusion, sluggish growth of host cells, suboptimal dosing, and treatment duration also enhance the likelihood of resistance emergence and treatment failure [24,25].

Efflux pumps also play a part: over-production of such pumps or alterations of membrane proteins diminish incorporation of fluoroquinolones. Additionally, Brucella persists in the acidic milieu of macrophage phagolysosomes, where fluoroquinolone activity is reduced. There are data to indicate that it is less effective than levofloxacin or ofloxacin in these low-pH settings [26,27].

In agreement with these results, Tekin et al. recorded increasing resistance to levofloxacin and ciprofloxacin in clinical Brucella isolates. Likewise, a genomic study of 24 human and animal B. melitensis strains by Ayoub et al. detected various efflux-pump genes (mprF, bepG, bepF, bepC, bepE). Interestingly, classical resistance mutations (gyrA, gyrB, folP, and rpoB) alone did not predict phenotypic resistance, highlighting the complex genotype–phenotype relationship [28,29].

Although fluoroquinolones possess numerous strengths, such as superior oral bioavailability, low nephrotoxicity, desirable pharmacokinetics, and a lack of requirement for drug-level monitoring, their regimens are not first-line for brucellosis because of rising relapse and resistance selection [30].

One of the reasons for ofloxacin's better performance than ciprofloxacin in the treatment of brucellosis needs the understanding of the disease's pathophysiology and the drug absorption, distribution, metabolism, and excretion (ADME) characteristics. Brucella is an intracellular pathogen that lives in the phagolysosomes of macrophages, which is a highly acidic (approximately pH 5) and therefore drug-resistant environment. Ofloxacin has been shown to penetrate cells more efficiently under acidic conditions and is more stable at low pH than ciprofloxacin. In contrast, ciprofloxacin is more ionized in an acidic environment; thus, its cellular permeability decreases, and the effective intracellular concentration becomes lower [31,32].

These limitations arise from two major factors: (a) intrinsic or acquired genetic resistance and (b) poor infiltration of the drug into intracellular reservoirs. Intrinsic or acquired QRDR mutations in gyrA and gyrB can confer intrinsic resistance prior to the start of treatment [33,34].

As an intracellular infection, brucellosis does not reach therapeutically stable intracellular levels with fluoroquinolones, as it does with tetracyclines or rifampin, and therefore accounts for treatment failure [35].

Despite strong in-vitro activity—a systematic review and meta-analysis performed by Beig et al. found low resistance levels to ofloxacin, sparfloxacin, pefloxacin, and lomefloxacin (2%, 1.6%, and 4.6%, respectively)—the results do not entirely reflect in vivo situations [36].

Many antibiotics showing in vitro efficacy are unable to reliably permeate the host cell membrane or reach the specific intracellular compartments within which Brucella is localized, leading to significant relapse rates [37].

Its capacity to infest and proliferate within non-phagocytic cells, as illustrated in the experiments of Detilleux et al. utilizing Vero cell systems, highlights its special capacity for intracellular proliferation, specifically within the rough endoplasmic reticulum [38].

Furthermore, there exists a sub-population of so-termed "persister" cells resistant to exposure to antibiotics and, upon discontinuation of treatment, rebound to yield recurrent infections.

This persistence, coupled with the intracellular lifestyle of the bacterium, affords intrinsic resistance to most antibiotics and emphasizes the need for novel research models to test drug efficacy (39). Given that the ofloxacin subgroup had different performances and better results than ciprofloxacin, it is recommended that ofloxacin be evaluated with a larger sample size in future trials.

Future research should focus on head-to-head comparisons between newer fluoroquinolones, such as levofloxacin or moxifloxacin, and standard regimens, with adequate follow-up to assess relapse. Understanding pharmacokinetic penetration into intracellular compartments could guide the development of optimized dosing strategies.

Our findings are consistent with the WHO recommendations discouraging the use of fluoroquinolones as the primary therapy for human brucellosis.

## Conclusion

From a practical standpoint, the data from the presented study confirm international suggestions that the first-line drug therapy for acute brucellosis should be doxycycline and rifampicin or streptomycin. In contrast, fluoroquinolone-based therapies, especially ciprofloxacin, are less effective and should not be used routinely. Only in exceptional situations, such as in patients who cannot tolerate tetracyclines or aminoglycosides, may ofloxacin-containing regimens be considered, and then only under close supervision, preferably within a structured clinical study. Although this analysis included data from 12 studies, the certainty of the evidence was moderate.

These studies are mostly old and do not conform to modern standards of comparison and have similar limitations such as small sample size, unblinded studies, and poor randomization reporting. The limited number of unpublished studies also raises the possibility that future data may alter these conclusions. For a more precise assessment, prospective, high-quality, well-controlled trials comparing new-generation fluoroquinolones (e.g., moxifloxacin or levofloxacin) with standard therapies are needed.

Overall, this systematic review provides strong evidence that fluoroquinolone-containing regimens are inferior to standard combinations, largely because of higher treatment failure rates. Ciprofloxacin shows the weakest outcomes; although ofloxacin offers a better safety profile and may slightly shorten fever duration, it does not significantly improve overall cure rates. These findings reinforce the continued use of doxycycline–rifampicin or doxycycline–streptomycin combinations as the mainstay of brucellosis therapy and caution against the routine replacement of these standard treatments with fluoroquinolones.

Fluoroquinolone-based regimens, particularly those containing ciprofloxacin, show inferior efficacy and should not be used as a replacement for standard therapies. Ofloxacin-based combinations may be reserved for selected cases with contraindications to first-line regimens.

## Authors’ Contributions

K.S. and M.M. developed the idea of the study and the methodology, and also oversaw the project. N.R, M.M, S.A. and R.M. performed the literature search, data abstraction, and quality evaluation. K.S., M.M. and N.M performed the statistical analysis and interpreted the data. Both authors developed the manuscript, reviewed it critically, and approved the final manuscript.

**Ethical approval**

Ethical approval was not required as this study used data from previously published research.

## Reference

1. Bahmani, N., & Bahmani, A. (2022). A review of brucellosis in the Middle East and control of animal brucellosis in an Iranian experience. *Reviews and Research in Medical Microbiology*, *33*(1), e63-e69.
2. Tuon, F. F., Gondolfo, R. B., & Cerchiari, N. (2017). Human-to-human transmission of Brucella–a systematic review. *Tropical Medicine & International Health*, *22*(5), 539-546.
3. Bahmani, N., & Bahmani, A. (2022). A review of brucellosis in the Middle East and control of animal brucellosis in an Iranian experience. *Reviews and Research in Medical Microbiology*, *33*(1), e63-e69.
4. Gorvel, J. P., & Moreno, E. (2002). Brucella intracellular life: from invasion to intracellular replication. *Veterinary microbiology*, *90*(1-4), 281-297.
5. Beig, M., Moradkasani, S., Goodarzi, F., & Sholeh, M. (2024). Prevalence of brucella melitensis and Brucella abortus fluoroquinolones resistant isolates: a systematic review and meta-analysis. *Vector-Borne and Zoonotic Diseases*, *24*(1), 1-9.
6. Darvishi, M., Nazer, M. R., Hashemi Rafsanjani, S. M., Nouri, M., & Soleiman-Meigooni, S. (2023). The Antibiotic Susceptibility of Brucella spp. Isolated from Human Samples: A Systematic Review and Meta-Analysis. *Iranian Journal of Medical Microbiology*, *17*(6), 620-628.
7. Meng, F., Pan, X., & Tong, W. (2018). Rifampicin versus streptomycin for brucellosis treatment in humans: A meta-analysis of randomized controlled trials. *PLoS One*, *13*(2), e0191993.
8. Turnidge, J. (1999). Pharmacokinetics and pharmacodynamics of fluoroquinolones. *Drugs*, *58*(Suppl 2), 29-36.
9. Falagas, M. E., & Bliziotis, I. A. (2006). Quinolones for treatment of human brucellosis: critical review of the evidence from microbiological and clinical studies. *Antimicrobial agents and chemotherapy*, *50*(1), 22-33.
10. Zhanel GG, Walkty A, Vercaigne L, Karlowsky JA, Embil J, Gin AS, Hoban DJ. The new fluoroquinolones: A critical review. Can J Infect Dis. 1999 May;10(3):207-38.
11. Akova, M., Uzun, O. M. R. U. M., Akalin, H. E., Hayran, M., Unal, S. E. R. H. A. T., & Gür, D. (1993).

Quinolones in treatment of human brucellosis: comparative trial of ofloxacin-rifampin versus doxycycline-rifampin. *Antimicrobial agents and chemotherapy*, *37*(9), 1831-1834.

12. Keramat, F., Ranjbar, M., Mamani, M., Hashemi, S. H., & Zeraati, F. (2009). A comparative trial of three therapeutic regimens: ciprofloxacin-rifampin, ciprofloxacin-doxycycline and doxycycline-rifampin in the treatment of brucellosis. *Tropical doctor*, *39*(4), 207-210.
13. Lang, R., Raoul, R., Sacks, T., & Shapiro, M. (1990). Failure of prolonged treatment with dprofloxadn in acute infections dueto Brucella melitensis. *Journal of Antimicrobial Chemotherapy*, *26*(6), 841-846.
14. Kalo, T., Novi, S., Nushi, A., & Dedja, S. (1996). Ciprofloxacin plus doxycycline versus rifampicin plus doxycycline in the treatment of acute brucellosis. *Médecine et maladies infectieuses*, *26*, 587-589.
15. Taghvaee, M. R., Nozadi, M., & Hassani, M. (2011). A comparison between doxycycline-rifampin and ciprofloxacin-rifampin regimens in the treatment of acute Brucellosis. *Indian Journal of Medical Sciences*, *65*(10), 436.
16. Saltoglu, N., Tasova, Y., Inal, A. S., Seki, T., & Aksu, H. S. (2002). Efficacy of rifampicin plus doxycycline versus rifampicin plus quinolone in the treatment of brucellosis. *Saudi medical journal*, *23*(8), 921-924.
17. Didgar, F., Sarmadian, H., ZARIN, F. N., Rafiee, M., & Choghae, M. (2012). Comparison between efficacy of ciprofioxacin-doxycycline and rifampin-doxycycline regimens in treatment of brucellosis.
18. Alp, E., Koc, R. K., Durak, A. C., Yildiz, O., Aygen, B., Sumerkan, B., & Doganay, M. (2006). Doxycycline plus streptomycin versus ciprofloxacin plus rifampicin in spinal brucellosis [ISRCTN31053647]. *BMC infectious diseases*, *6*(1), 72.
19. Alikhani, A., & Heidarzadeh, A. (2007). A new therapeutic management in acute uncomplicated brucellosis: Comparison between three month-ofloxacin and doxycycline-based regimens. *Journal of Isfahan Medical School*, *25*(85), 60-55.
20. Agalar, C., Usubutun, S., & Turkyilmaz, R. (1999). Ciprofloxacin and rifampicin versus doxycycline and rifampicin in the treatment of brucellosis. *European Journal of Clinical Microbiology and Infectious Diseases*, *18*(8), 535-538.
21. Bayindir, Y. A. Ş. A. R., Sonmez, E., Aladag, A., & Buyukberber, N. (2003). Comparison of five antimicrobial regimens for the treatment of brucellar spondylitis: a prospective, randomized study. *Journal of chemotherapy*, *15*(5), 466-471.
22. Karabay, O., Sencan, I., Kayas, D., & Şahin, I. (2004). Ofloxacin plus rifampicin versus doxycycline plus rifampicin in the treatment of brucellosis: a randomized clinical trial [ISRCTN11871179]. *BMC infectious diseases*, *4*(1), 18.
23. Rodríguez Tarazona E, García Rodríguez JÁ, Muñoz Bellido JL. Emergence of quinolone-resistant, topoisomerase-mutant Brucella after treatment with fluoroquinolones in a macrophage experimental infection model. Enferm Infecc Microbiol Clin. 2015 Apr;33(4):248-52
24. Beig M, Moradkasani S, Goodarzi F, Sholeh M. Prevalence of Brucella melitensis and Brucella abortus Fluoroquinolones Resistant Isolates: A Systematic Review and Meta-Analysis. Vector Borne Zoonotic Dis. 2024 Jan;24(1):1-9.
25. Blanc L, Daudelin IB, Podell BK, Chen PY, Zimmerman M, Martinot AJ, Savic RM, Prideaux B, Dartois

V. High-resolution mapping of fluoroquinolones in TB rabbit lesions reveals specific distribution in immune cell types. Elife. 2018 Nov 14;7:e41115.

26. Valdezate S, Navarro A, Medina-Pascual MJ, Carrasco G, Saéz-Nieto JA. Molecular screening for rifampicin and fluoroquinolone resistance in a clinical population of Brucella melitensis. J Antimicrob Chemother. 2010 Jan;65(1):51-3.
27. Falagas, M. E., & Bliziotis, I. A. (2006). Quinolones for treatment of human brucellosis: critical review of the evidence from microbiological and clinical studies. *Antimicrobial agents and chemotherapy*, *50*(1), 22-33.
28. Ayoub, H., Kumar, M. S., Mehta, R., Thomas, P., Dubey, M., Dhanze, H., & Amachawadi, R. G. (2024). Exploring genetic determinants of antimicrobial resistance in Brucella melitensis strains of human and animal origin from India. *Frontiers in Microbiology*, 15, 1474957.
29. Tekin, M. K., Erbağcı, E., & İnal, N. (2024). In-Vitro Antimicrobial Susceptibility Results of Brucella Species in Eastern Türkiye. *Journal of Global Antimicrobial Resistance*, *39*, 32.
30. Pappas, G., Christou, L., Akritidis, N., & Tsianos, E. V. (2006). Quinolones for brucellosis: treating old diseases with new drugs. *Clinical Microbiology and Infection*, 12(9), 823-825.
31. Turkmani A, Psaroulaki A, Christidou A, Samoilis G, Mourad TA, Tabaa D, Tselentis Y. Uptake of ciprofloxacin and ofloxacin by 2 Brucella strains and their fluoroquinolone-resistant variants under different conditions. An in vitro study. Diagnostic microbiology and infectious disease. 2007 Dec 1;59(4):447-51.
32. Drakopoulos AI, Ioannou PC. Spectrofluorimetric study of the acid–base equilibria and complexation behavior of the fluoroquinolone antibiotics ofloxacin, norfloxacin, ciprofloxacin and pefloxacin in aqueous solution. *Analytica Chimica Acta*. 1997 Nov 10;354(1-3):197-204.
33. Ravanel, N., Gestin, B., & Maurin, M. (2009). *In vitro* selection of fluoroquinolone resistance in Brucella melitensis. *International journal of antimicrobial agents*, *34*(1), 76-81.
34. Turkmani, A., Psaroulaki, A., Christidou, A., Chochlakis, D., Tabaa, D., & Tselentis, Y. (2008). In vitro-selected resistance to fluoroquinolones in two Brucella strains associated with mutational changes in gyrA. *International journal of antimicrobial agents*, *32*(3), 227-232.
35. Gamazo, C., Concepcion Lecaroz, M., Prior, S., Isabel Vitas, A., Campanero, M. A., Irache, J. M., & Jose Blanco-Prieto, M. (2006). Chemical and biological factors in the control of Brucella and brucellosis. *Current Drug Delivery*, *3*(4), 359-365.
36. Beig, M., Moradkasani, S., Goodarzi, F., & Sholeh, M. (2024). Prevalence of brucella melitensis and Brucella abortus fluoroquinolones resistant isolates: a systematic review and meta-analysis. *Vector-Borne and Zoonotic Diseases*, *24*(1), 1-9.
37. Gamazo, C., Concepcion Lecaroz, M., Prior, S., Isabel Vitas, A., Campanero, M. A., Irache, J. M., & Jose Blanco-Prieto, M. (2006). Chemical and biological factors in the control of Brucella and brucellosis. *Current Drug Delivery*, *3*(4), 359-365.
38. Detilleux, P. G., Deyoe, B. L., & Cheville, N. F. (1990). Penetration and intracellular growth of Brucella abortus in nonphagocytic cells in vitro. *Infection and immunity*, *58*(7), 2320-2328.
39. Mode, S., Ketterer, M., Québatte, M., & Dehio, C. (2022). Antibiotic persistence of intracellular Brucella abortus. *PLoS neglected tropical diseases*, *16*(7), e0010635.

## Table 2. Subgroup analysis of fluoroquinolone regimens in brucellosis treatment

| Variable | Duration | | Intervention Regimen | | Location | | | Sample size | | Follow-up | |
| --- | --- | --- | --- | --- | --- | --- | --- | --- | --- | --- | --- |
| **Therapeutic**  **Failure** | 6 weeks≥ | 6 weeks< | Ciprofloxacin | Ofloxacin | Iran | Turkey | Other  Country | 50> | 50≤ | 6 Months≥ | 6 Months< |
| Number of  comparisons | 7 | 3 | 7 | 3 | 4 | 4 | 2 | 5 | 5 | 3 | 6 |
| Risk Ratio | 1.93 | 2.48 | 2.59 | 1.35 | 2.7  5 | 1.38 | 2.79 | 2.19 | 2.24 | 1.57 | 2.27 |
| P value | 0.07 | 0.001 | 0.001 | 0.54 | 0.0  01 | 0.4 | 0.2 | 0.09 | 0.004 | 0.36 | 0.02 |
| I2 (%) | 0% | 0% | 0% | 0% | 0% | 0% | 13% | 0% | 0% | 0% | 0% |
| P-  heterogenity | 0.6 | 0.9 | 0.8 | 0.8 | 0.2 | 0.9 | 0.2 | 0.4 | 0.9 | 0.6 | 0.6 |
| **Relapse** | 6 weeks≥ | 6 weeks< | Ciprofloxacin | Ofloxacin | Iran | Turkey | Other  Country | 50> | 50≤ | 6 Months≥ | 6 Months< |
| Number of  comparisons | 6 | 4 | 6 | 4 | 3 | 5 | 2 | 5 | 5 | 3 | 6 |
| Risk Ratio | 2.01 | 1.35 | 1.78 | 1.44 | 2.0  0 | 1.45 | 2.79 | 1.83 | 1.45 | 1.17 | 2.4 |
| P value | 0.1 | 0.4 | 0.1 | 0.4 | 0.3 | 0.3 | 0.3 | 0.1 | 0.3 | 0.8 | 0.03 |
| I2 (%) | 0% | 0% | 75% | 0% | 44  % | 0% | 13.5% | 0% | 0% | 0% | 0% |
| P-  heterogenity | 0.5 | 0.6 | 0.4 | 0.8 | 0.1 | 0.9 | 0.2 | 0.7 | 0.4 | 0.8 | 0.6 |
| **Adverse**  **Event** | 6 weeks≥ | 6 weeks< | Ciprofloxacin | Ofloxacin | Iran | Turkey | Other  Country | 50> | 50≤ | 6 Months≥ | 6 Months< |
| Number of  comparisons | 5 | 3 | 4 | 4 | 4 | 3 | 1 | 3 | 5 | 4 | 2 |
| Risk Ratio | 1.08 | 0.56 | 0.97 | 0.24 | 0.5  8 | 0.55 | 3.26 | 1.64 | 0.4 | 0.39 | 0.24 |
| P value | 0.9 | 0.1 | 0.9 | 0.000 | 0.6 | 0.2 | 0.4 | 0.5 | 0.07 | 0.01 | 0.05 |
| I2 (%) | 77% | 40% | 0% | 64% | 55  % | 73% | 0% | 20 | 64% | 41.8% | 85.3% |

| P-  heterogenity | 0.01 | 0.1 | 0.8 | 0.03 | 0.0  8 | 0.02 | 1 | 0.2 | 0.02 | 0.1 | 0.009 |
| --- | --- | --- | --- | --- | --- | --- | --- | --- | --- | --- | --- |
| **Time to**  **Defervescenc e** | 6 weeks≥ | 6 weeks< | Ciprofloxacin | Ofloxacin | Iran | Turkey | Other Country | 50> | 50≤ | 6 Months≥ | 6 Months< |
| Number of  comparisons | 4 | 1 | 2 | 3 | 1 | 4 | 0 | 3 | 2 | 2 | 3 |
| Risk Ratio | 1.2 | 0.3 | 0.63 | 1.25 | 0.3 | 1.2 | - | 1.2 | 0.48 | 0.78 | 1.13 |
| P value | 0.000 | 0.4 | 0.09 | 0.000 | 0.4 | 0.000 | - | 0.00  0 | 0.1 | 0.1 | 0.001 |
| I2 (%) | 0% | 0% | 29% | 0% | 0% | 0% | - | 0% | 0% | 64% | 0% |
| P-  heterogenity | 0.9 | 1 | 0.2 | 0.9 | 1 | 0.9 | - | 0.9 | 0.3 | 0.09 | 0.9 |

**Table 3. Assessment of publication bias in the impact of fluoroquinolone on brucellosis**

|  | Corrected Effect size | | Begg’s rank correction test | | Egger’s linear regression test | | | | Fail  safe N test |
| --- | --- | --- | --- | --- | --- | --- | --- | --- | --- |
|  | VMD | 95%CI | Kendal  Tau | P | Intercept | 95%CI | T  value | P | N |
| Therapeutic  Failure | 2.23 | 1.39-3.57 | -0.22 | 0.37 | -0.26 | -1.79-1.25 | 0.4 | 0.69 | 10 |
| Relapse | 1.63 | 0.89-2.97 | 0.17 | 0.47 | 1.04 | -1.50-3.58 | 0.94 | 0.37 | 10 |
| Adverse event | 0.43 | 0.18-1.03 | 0.10 | 0.71 | 1.37 | -2.58-5.32 | 0.84 | 0.42 | 8 |
| Time to  Defervescence | 0.72 | 0.33-1.12 | 0.1 | 0.8 | 1.63 | -4.19-7.47 | 0.89 | 0.43 | 5 |

Table 1. Demographic characteristics of the included studies

| First author/  year | Location | Mean age years (I/C) | Study Design | Sample size  Total(I/C) | Female/Male  (I/C) | Intervention  Group | Control Group | Duration | Population |
| --- | --- | --- | --- | --- | --- | --- | --- | --- | --- |
| Murat Akova/1993 | Ankara/Turkey | (34.4±14.3)/(37.8±15.1) | Parallel RCT | 61(30/31) | (16/14)/(14/17) | Ofloxacin  (400 mg once daily) Rifampicin (600 mg once  daily) | Doxycycline (200 mg once daily)  Rifampicin (600 mg once daily) | 6 weeks | Adults with acute uncomplicated brucellosis |
| Keramat/2009 | Hamedan/Iran | 39.67/43.66 | Parallel RCT | 178(61/117) | 61/117 | Doxycycline (200 mg once daily) Rifampicin (600 mg once daily) | Ciprofloxacin (500 mg PO twice daily) Rifampicin (600 mg once daily)  OR  Ciprofloxacin (500 mg PO twice daily) Doxycycline (200 mg  once daily) | 8-12  weeks | Adults with acute uncomplicated brucellosis |
| Lang/1990 | Israel | Range (18-58) | Parallel RCT | 10(6/4) | (3/3)/(3/1) | Ciprofloxacin (500 mg PO  twice daily) | Doxycycline (200 mg once daily)  Rifampicin (600 mg once daily) | 6 weeks | Adults with acute uncomplicated brucellosis |

| Kalo/1996 | Tirana, Albania | Total (31.8±13.5) | Parallel RCT | 24(12/12) | 10/14 | Ciprofloxacin (500 mg PO  twice daily) Doxycycline (200 mg once  daily) | Doxycycline (200 mg once daily)  Rifampicin (600 mg once daily) | 6 weeks | Adults with acute uncomplicated brucellosis |
| --- | --- | --- | --- | --- | --- | --- | --- | --- | --- |
| Erfanian/2011 | Mashhad/Iran | (36.8±10.9)/(35.2±10) | Prospective observational comparative trial (not blinded  RCT) | 50(23/27) | (12/15)/(10/13) | Ciprofloxacin (500 mg PO  twice daily) Rifampicin (600 mg once  daily) | Doxycycline (200 mg once daily)  Rifampicin (600 mg once daily) | 6 weeks | Adults with acute uncomplicated brucellosis |
| Saltoglu/2002 | Adana, Turke | 36.8±11.3 | Prospective comparative (not blinded  RCT) | 57(27/30) | (21/6)/(22/8) | Ofloxacin  (400 mg once daily) | Doxycycline (200 mg once daily)  Rifampicin (600 mg  once daily) | 45 day | acute/chronic (ostearticular included) |

|  |  |  |  |  |  | Rifampicin (600 mg once  daily) |  |  |  |
| --- | --- | --- | --- | --- | --- | --- | --- | --- | --- |
| Didgar/2012 | Arak/Iran | (38.6±16.7)/(35.4±16.4) | Randomized controlled trial (triple blind) | 80(40/40) | 20/60 | Ciprofloxacin (500 mg PO  twice daily) Doxycycline | Doxycycline (200 mg once daily)  Rifampicin (600 mg once daily) | 8 weeks | Adults with acute uncomplicated brucellosis |

|  |  |  |  |  |  | (200 mg once daily) |  |  |  |
| --- | --- | --- | --- | --- | --- | --- | --- | --- | --- |
| Alavi/2014 | Ahvaz/Iran | (31.6±14.3)/(31.3±13.6) | Parallel RCT | 107(54/53) | (29/25)/(26/27) | Ciprofloxacin (500 mg PO  twice daily) Doxycycline (200 mg once daily) | Doxycycline (200 mg once daily)  Rifampicin (600 mg once daily) | 8 weeks | Adults with acute uncomplicated brucellosis |
| Alikhani/2007 | Tonekabon/Iran | Range (15-80) | Single blinded Parallel RCT | 78(38/40) | NR | Ofloxacin  (400 mg once daily) Rifampicin (600 mg once daily) Streptomycin (750–1000 mg  IM daily) | Doxycycline (200 mg once daily)  Rifampicin (600 mg once daily) Streptomycin (750–1000 mg IM daily) | 3 months | Adults with acute uncomplicated brucellosis |
| Agalar/1999 | Ankara/Turkey | (37.8±13.9)/(37.1±15.8) | Parallel RCT | 40(20/20) | (12/8)/(7/13) | Ciprofloxacin (500 mg PO  twice daily) Rifampicin (600 mg once  daily) | Doxycycline (200 mg once daily)  Rifampicin (600 mg once daily) | 45 days | Hospitalized brucellosis patients |

| Karabay/2004 | Duzce/Turkey | Range(18-60/24-61) | Parallel RCT | 29(15/14) | (2/13)/(3/11) | Ofloxacin  (400 mg once daily) Rifampicin (600 mg once  daily) | Doxycycline (200 mg once daily)  Rifampicin (600 mg once daily) | 45 days | Hospitalized brucellosis patients |
| --- | --- | --- | --- | --- | --- | --- | --- | --- | --- |
| Bayindir/2003 | Malatya/Turkey | Overall: 40.5 | Parallel RCT | 102(19/83) | 46/56 | Ofloxacin  (400 mg once daily) Rifampicin (600 mg once daily) | ST=20  (Streptomycin 1g IM  + Tetracycline 500 mg PO q6h) SD=21(Streptomycin 1g IM + Doxycycline 100 mg PO q12h) DR=20 (Doxycycline  100 mg PO q12h + Rifampicin 15 mg/kg  PO qd) SDR=22  (Streptomycin 1g IM  + Doxycycline 100 mg PO q12h +  Rifampicin 15 mg/kg  PO qd) | 45 days | Brucellar spondylitis |

Figure	[Click here to access/download;Figure;Plots.docx](https://www2.cloud.editorialmanager.com/pntd/download.aspx?id=1515606&guid=dc5a91d0-368c-42a3-aa06-7c69c49d80cb&scheme=1)[<https://www2.cloud.editorialmanager.com/pntd/download.aspx?id=1515606&guid=dc5a91d0-368c-42a3-aa06-7c69c49d80cb&scheme=1>](https://www2.cloud.editorialmanager.com/pntd/download.aspx?id=1515606&guid=dc5a91d0-368c-42a3-aa06-7c69c49d80cb&scheme=1)

Therapeutic Failure

| Groue by | Study name | Statistics for each study | |  | | Risk ratio and 95% Cl  - | |
| --- | --- | --- | --- | --- | --- | --- | --- |
| Intervention Duration group |  |  |  |  | |  | |
|  | Risk Lower Upper | |  |  | |  | |
|  | ratio | limit limit Z-Value p-Value | | | |  | |
| Less equal 6 weeks | Agalar, 19991.500 | 0.280 8.036 | 0.473 | 0.636 | |  | |
| Less equal 6 weeks | Erfanian, 2018.217 | 1.090 61.954 | 2.044 | 0.041 | |  | |
| Less equal 6 weeks | Kalo, 1996 1.000 | 0.070 14.208 | 0.000 | 1.000 | |  | |
| Less equal 6 weeks Less equal 6 weeks Less equal 6 weeks Less equal 6 weeks Less equal 6 weeks More 6weeks More 6weeks More 6weeks  More 6weeks Overall | Karabay, 20C0.933 Lang, 1990 7.857  Akova, 1993 0.968  Bayindir, 2001.754  1.931  Alavi. 2014 2.290  Keramat, 2009806  Oidgar, 20122.000  2.484  2.231 | 0.151 5.758  0.551112.089  0.063 14.779  0.485 6.350  0.939 3.970  0.625 8.389  1.212 6.499  0.537 7.448  1.334 4.623  1.393 3.572 | -0.074  1.520  -0.024  0.857  1.789  1.251  2.408  1.033  2.869  3.341 | 0.941  0.128  0.981  0.392  0.074  0.211  0.016  0.301  0.004  0.001 | **0.01** | 0.1 | �..   - 10 100 |
|  |  |  |  |  | **FQ-Regimen Standard-Regimen** | | |
| **Fixed** model |  |  |  |  |  | | |

**Groue by:	Study:name	Statistics for each study:**

**Intervention Group	Risk Lower Upper**

**ratio limit	limit Z-Value p-Value**

-•

◄ ►•

-

10

100

**Risk ratio and 95'/, Cl**

| **Ciprofloxacin-based** | Agalar, 1999 1.500 | 0.280 8.036 | | 0.473 | 0.636 | | |
| --- | --- | --- | --- | --- | --- | --- | --- |
| **Ciprofloxacin-based** | Erfanian, 2011.217 | 1.090 61.954 | | 2.044 | 0.041 | | |
| **Ciprofloxacin-based** | Kalo, 1996 1.000 | 0.070 14.208 | | 0.000 | 1.000 | | |
| **Ciprofloxacin-based** | Alavi, 2014 2.290 | 0.625 8.389 | | 1.251 | 0.211 | | |
| **Ciprofloxacin-based** | Lang, 1990 7.857 | 0.551112.089 | | 1.520 | 0.128 | | |
| **Ciprofloxacin-based** | Keramat, 20011.806 | 1.212 | 6.499 | 2.408 | 0.016 |  |  |
| **Ciprofloxacin-based** | Didgar, 2012 2.000 | 0.537 | 7.448 | 1.033 | 0.301 |  |  |
| **Ciprofloxacin-based** | 2.592 | 1.515 | 4.432 | 3.479 | 0.001 |  |  |
| **Ofloxacin-based** | Karabay, 200.0.933 | 0.151 | 5.758 | -0.074 | 0.941 |  |  |
| Ofloxacin-based | Akova, 1993 0.968 | 0.063 | 14.779 | -0.024 | 0.981 |  |  |
| **Ofloxacin-based** | Bayindir, 20031.754 | 0.485 | 6.350 | 0.857 | 0.392 |  |  |
| **Ofloxacin-based** | 1.353 | 0.508 | 3.605 | 0.604 | 0.546 |  |  |
| Overall | 2.231 | 1.393 | 3.572 | 3.341 | 0.001 |  |  |
|  |  |  |  |  |  | **0.01** | **0.1** |

**FQ-Regimen Standard-Regimen**

Fixed model

| **Group by Study name** | |  | **Statistics for each study** | | |  |  | **Risk ratio and 95% Cl** | | | | | |  |
| --- | --- | --- | --- | --- | --- | --- | --- | --- | --- | --- | --- | --- | --- | --- |
| **Location** |  |  |  |  |  |  |  |  |  |  |  | | |  |
|  |  | **Risk Lower** | | **Upper** |  |  |  |  |  |  |  | | |  |
| Albania Albania Iran Iran Iran Iran | **ratio**  Kalo, 1996 1.000  1.000  Erfanian, 20118.217  Alavi, 2014 2.290  Keramat, 20092.806  Didgar, 2012 2.000 | | **limit limit 2-Value p-Value**  0.070 14.208 0.000 1.000  0.070 14.208 0.000 1.000  1.090 61.954 2.044 0.041  0.625 8.389 1.251 0.211  1.212 6.499 2.408 0.016  0.537 7.448 1.033 0.301 | | | |  |  |  | -  • | | | |  |
| Iran  Israel  Israel | Lang, 1990 | 2.754  7.857  7.857 | 1.521 4.988  0.551 112.089  0.551 112.089 | | 3.343  1.520  1.520 | 0.001  0.128  0.128 |  |  |  |  |  | -- |  |  |
| Turkey | Agalar, 1999 | 1.500 | 0.280 | 8.036 | 0.473 | 0.636 |  |  |  |  |  | | |  |
| Turkey | Karabay, 20040.933 | | 0.151 | 5.758 | -0.074 | 0.941 |  |  |  |  |  | | |  |
| Turkey Turkey | Akova, 1993 0.968  Bayindir, 2003 **1.**754 | | 0.063  0.485 | 14.779  6.350 | -0.024  0.857 | 0.981  0.392 |  |  |  | - | |  |  |  |
| Turkey |  | 1.389 | 0.596 | 3.237 | 0.760 | 0.447 |  |  | ◄ |  |  | | |  |
| Overall |  | 2.231 | 1.393 | 3.572 | 3.341 | 0.001 |  |  |  | � |  | | |  |
|  |  |  |  |  |  |  | **0.01** | **0.1** |  |  | **10** | | | **100** |
|  |  |  |  |  |  |  |  | **FQ-Regimen Standard-Regimen** | | | | | | |
| **Fixed model** |  |  |  |  |  |  |  |  |  |  |  | | |  |

**Stud:t'n. ame	Statistics with stud:t'. removed	Risk ratio (95% Cl)**

**with study removed**

|  | **Point** | **Lower limit** | **Upper limit** | **Z-Value** | **p-Value** |
| --- | --- | --- | --- | --- | --- |
| Agalar, 1999 | 2.308 | 1.413 | 3.768 | 3.343 | 0.001 |
| Erfanian, 2011 | 2.070 | 1.276 | 3.359 | 2.946 | 0.003 |
| Kalo, 1996 | 2.290 | 1.419 | 3.694 | 3.395 | 0.001 |
| Karabay, 2004 | 2.375 | 1.459 | 3.866 | 3.479 | 0.001 |
| Alavi, 2014 | 2.222 | 1.341 | 3.682 | 3.099 | 0.002 |
| Lang, 1990 | 2.142 | 1.328 | 3.455 | 3.121 | 0.002 |
| Keramat, 2009 | 2.008 | 1.138 | 3.545 | 2.405 | 0.016 |
| Akova, 1993 | 2.289 | 1.419 | 3.691 | 3.396 | 0.001 |
| Didgar, 2012 | 2.267 | 1.369 | 3.753 | 3.182 | 0.001 |
| Bayindir, 2003 | 2.315 | 1.396 | 3.839 | 3.254 | 0.001 |
|  | 2.231 | 1.393 | 3.572 | 3.341 | 0.001 |

•

•

-

•

•

•

**0.01	0.1	1**• **10	100**

**FQ-Regimen Standard-Regimen**

Random model

**Risk ratio and 95•1. Cl**

| **Groue b::t: Stud::t: name Statistics for each stud::t:**  **Sample size group Risk Lower Upper**  **ratio limit limit Z-Value p-Value**  **50 patients or more** Alavi, 2014 2.290 0.625 8.389 1.251 0.211  **50 patients or more** Keramat, 20011.806 1.212 6.499 2.408 0.016  **50 patients or more** Akova, 1993 0.968 0.063 14.779 -0.024 0.981  **50 patients or more** Didgar, 2012 2.000 0.537 7.448 1.033 0.301  **50 patients or more** Bayindir, 20031.754 0.485 6.350 0.857 0.392  **50 patients or more** 2.244 1.297 3.883 2.891 0.004  Less than 50 patients Agalar, 1999 1.500 0.280 8.036 0.473 0.636  **Less than 50 patients** Erfanian, 2011.217 1.090 61.954 2.044 0.041 | | | | | |  | - | | |  |
| --- | --- | --- | --- | --- | --- | --- | --- | --- | --- | --- |
| Less than 50 patients | Kalo, 1996 1.000 | | 0.070 14.208 | 0.000 | 1.000 |  |  |  |  |  |
| Less than 50 patients | Karabay, 2004l.933 | | 0.151 5.758 | -0.074 | 0.941 |  |  |  |  |  |
| Less than 50 patients | Lang, 1990 | 7.857 | 0.551112.089 | 1.520 | 0.128 | | | | | |
| Less than 50 patients Overall | 2.193  2.231 | | 0.875 5.494  1.393 3.572 | 1.676  3.341 | 0.094  0.001 | **0.01** | 0.1 | � • | 10 | 100 |
|  |  | |  |  |  |  | **FQ-Regimen Standard-Regimen** | | | |
| Random model |  | |  |  |  |  |  |  |  |  |

-

-

-

�-

-

# Therapeutic Failure

**Stud name	Statistics for each stud**

**Risk ratio and 95% Cl**

--

| **Risk**  **ratio** | | **Lower**  limit | **Upper**  limit | **Z-Value** | **p-Value** |
| --- | --- | --- | --- | --- | --- |
| Agalar, 1999 | 1.500 | 0.280 | 8.036 | 0.473 | 0.636 |
| Erfanian, 2011 | 8.217 | 1.090 | 61.954 | 2.044 | 0.041 |
| Kalo, 1996 | 1.000 | 0.070 | 14.208 | 0.000 | 1.000 |
| Karabay, 2004 | 0.933 0.151 5.758 -0.074 0.941 | | | | |
| Alavi, 2014 | 2.290 0.625 8.389 1.251 0.211 | | | | |
| Lang, 1990 | 7.857 0.551 112.089 1.520 0.128 | | | | |
| Keramat, 2009 | 2.806 1.212 6.499 2.408 0.016 | | | | |
| Akova, 1993 | 0.968 0.063 14.779 -0.024 0.981 | | | | |
| Didgar, 2012 | 2.000 0.537 7.448 1.033 0.301 | | | | |
| Bayindir, 2003 | 1.754 0.485 6.350 0.857 0.392 | | | | |
|  | 2.231 1.393 3.572 3.341 0.001 | | | | |

-

--

----

--

-

**0.01**

**0.1**	1• 10	**100**

**FQ-Regimen Standard-Regimen**

**Fixed** model

Relapse

Group by

Intervention duration sub group2

Study name	Statistics for each study Risk Lower Upper

ratio limit limit Z-Vatue p-Value

Risk ratio and 95% Cl

| Less equal 6 weeks | Agalar, 1999 | 1.500 | 0.280 8.036 | 0.473 | 0.636  | | | | |
| --- | --- | --- | --- | --- | --- | --- | --- | --- | --- |
| Less equal 6 weeks | Erfanian, 2011 | 8.217 | 1.090 61.954 | 2.044 | 0.041 | | | | |
| Less equal 6 weeks | Kalo, 1996 | 1.000 | 0.070 14.208 | 0.000 | 1.000 | | | | |
| Less equal 6 weeks | Karabay, 2004 | 0.933 | 0.151 5.758 | -0.074 | 0.941 | | | | |
| Less equal 6 weeks | Lang, 1990 | 7.857 | 0.551 112.089 | 1.520 | 0.128 | | | | |
| Less equal 6 weeks | Akova, 1993 | 0.968 | 0.063 14.779 | -0.024 | 0.981 | | | | |
| Less equal 6 weeks | 2.018 | | 0.845 4.817 | 1.581 | 0.114 |  |  |  |  |
| More than6 weeks | Saltoghlu, 2002 1.111 | | 0.168 7.353 | 0.109 | 0.913 |  |  |  |  |
| More than6 weeks | Alavi, 2014 1.963 | | 0.183 21.006 | 0.558 | 0.577 |  |  |  |  |
| More than6 weeks | Keramat, 2009 0.802 | | 0.215 2.992 | -0.329 | 0.742 |  |  |  |  |
| More than6 weeks | Bayindir, 2003 2.632 | | 0.579 11.970 | 1.252 | 0.211 |  |  |  |  |
| More than6 weeks | 1.352 | | 0.593 3.085 | 0.717 | 0.473 |  |  |  |  |
| Overall | 1.634 | | 0.898 2.973 | 1.608 | 0.108 |  |  |  |  |
|  |  | |  |  |  | 0.01 | 0.1 | 10 | 100 |

**FQ-Regimen Standard-Regimen**

**Fixed model**

=Gecro"'u"p"b"'y,-�----- **Study name	Statistics for each study Intervention Group	Risk Lower Upper**

**ratio limit	limit Z-Value p-Value**

**Risk ratio and 95% Cl**

| **Ciprofloxacin-based** | Agalar, 1999 1.500 | 0.280 8.036 | | 0.473 | 0.636  | | | | | |
| --- | --- | --- | --- | --- | --- | --- | --- | --- | --- | --- |
| **Ciprofloxacin-based** | Erfanian, 20118.217 | 1.090 61.954 | | 2.044 | 0.041 | | | | | |
| **Ciprofloxacin-based** | Kalo, 1996 1.000 | 0.070 14.208 | | 0.000 | 1.000 | | | | | |
| **Ciprofloxacin-based** | Alavi, 2014 1.963 | 0.183 21.006 | | 0.558 | 0.577 | | | | | |
| **Ciprofloxacin-based** | Lang, 1990 7.857 | 0.551112.089 | | 1.520 | 0.128 | | | | | |
| **Ciprofloxacin-based** | Keramat, 2009J.802 | 0.215 | 2.992 | -0.329 | 0.742 |  |  |  |  |  |
| **Ciprofloxacin-based** | 1.785 | 0.814 | 3.916 | 1.447 | 0.148 |  |  |  |  |  |
| **Ofloxacin-based** | Saltoghlu, 2002.111 | 0.168 | 7.353 | 0.109 | 0.913 |  |  |  |  |  |
| **Ofloxacin-based** | Karabay, 20040.933 | 0.151 | 5.758 | -0.074 | 0.941 |  |  |  |  |  |
| **Ofloxacin-based** | Akova, 1993 0.968 | 0.063 | 14.779 | -0.024 | 0.981 |  |  |  |  |  |
| **Ofloxacin-based** | Bayindir, 20032.632 | 0.579 | 11.970 | 1.252 | 0.211 |  |  |  |  |  |
| **Ofloxacin-based** | 1.447 | 0.570 | 3.673 | 0.778 | 0.437 |  |  |  |  |  |
| Overall | 1.636 | 0.898 | 2.983 | 1.607 | 0.108 |  |  |  |  |  |
|  |  |  |  |  |  | **0.01** | **0.1** | **10** | | **100** |

**FQ-Regimen Standard-Regimen**

**Random model**

| **�� _r�-���� b-:-- --** | | **Study name** | | **Statistics for each study** | | |  | **Risk ratio and 95% Cl**  -  ◄ | | | |  |
| --- | --- | --- | --- | --- | --- | --- | --- | --- | --- | --- | --- | --- |
| **0** | **0** | **Risk Lower Upper** | | | | | | | | | | |
|  | | **ratio** | | **limit** | **limit Z-Value p-Value** | | |  |  | |  |  |
| Albania | | Kalo, 1996 | 1.000 | 0.070 | 14.208 | 0.000 | 1.000 | | | | | |
| Albania | | 1.000 | | 0.070 | 14.208 | 0.000 | 1.000 |  |  | |  |  |
| Iran Iran Iran Iran Israel  Israel | | Erfanian, 2011 8.217  Alavi, 2014 1.963  Keramat, 2009 0.802  2.005  Lang, 1990 7.857  7.857 | | 1.090 61.954  0.183 21.006  0.215 2.992  0.478 8.414  0.551112.089  0.551112.089 | | 2.044  0.558  -0.329  0.951  1.520  1.520 | 0.041  0.577  0.742  0.342  0.128  0.128 | -- | | -  - | -  -- | |
| Turkey | | Agalar, 1999 | 1.500 | 0.280 | 8.036 | 0.473 | 0.636 | - | | | | |
| Turkey | | Saltoghlu, 20021.111 | | 0.168 | 7.353 | 0.109 | 0.913 |  |  | |  |  |
| Turkey | | Karabay, 2004 0.933 | | 0.151 | 5.758 | -0.074 | 0.941 |  |  | |  |  |
| Turkey Turkey Turkey Overall | | Akova, 1993 0.968  Bayindir, 2003 2.632  1.459  1.694 | | 0.063  0.579  0.646  0.873 | 14.779  11.970  3.295  3.286 | -0.024  1.252  0.910  1.559 | 0.981  0.211  0.363  0.119 | **0.01 0.1** | **t** | | **10** | **100** |
|  | |  | |  |  |  |  | **FQ-Regimen Standard-Regimen** | | | | |
| **Random model** | |  | |  |  |  |  |  |  | |  |  |

# Relapse

**Stud:t name	Statistics for each stud:t	Risk ratio and 95% Cl Risk	Lower	Upper**

**ratio	limit	limit	Z-Value p-Value**

| Agalar, 1999 | 1.500 | 0.280 | 8.036 | 0.473 | 0.636  |  | | | |
| --- | --- | --- | --- | --- | --- | --- | --- | --- | --- |
| Erfanian, 2011 | 8.217 | 1.090 | 61.954 | 2.044 | 0.041 |  |  |  |  |
| Saltoghlu, 2002 | 1.111 | 0.168 | 7.353 | 0.109 | 0.913 |  |  |  |  |
| Kalo, 1996 | 1.000 | 0.070 | 14.208 | 0.000 | 1.000 |  |  |  |  |
| Karabay, 2004 | 0.933 | 0.151 | 5.758 | -0.074 | 0.941 |  |  |  |  |
| Alavi, 2014 | 1.963 | 0.183 | 21.006 | 0.558 | 0.577 |  |  |  |  |
| Lang, 1990 | 7.857 | 0.551 | 112.089 | 1.520 | 0.128 |  |  |  |  |
| Keramat, 2009 | 0.802 | 0.215 | 2.992 | -0.329 | 0.742 |  |  |  |  |
| Akova, 1993 | 0.968 | 0.063 | 14.779 | -0.024 | 0.981 |  |  |  |  |
| Bayindir, 2003 | 2.632 | 0.579 | 11.970 | 1.252 | 0.211 |  |  |  |  |
|  | 1.634 | 0.898 | 2.973 | 1.608 | 0.108 |  |  |  |  |
|  |  |  |  |  |  | **0.01** | **0.1 1** | **10** | **100** |

FQ-Regimen Standard-Regimen

Random model

|  | **Point** | **limit** | **limit** | **Z-Value** | **p-Value** |  | | |
| --- | --- | --- | --- | --- | --- | --- | --- | --- |
| Agalar, 1999 | 1.655 | 0.872 | 3.140 | 1.540 | 0.123 |  |  |  |
| Erfanian, 2011 | 1.399 | 0.747 | 2.618 | 1.050 | 0.294 |  |  |  |
| Saltoghlu, 2002 | 1.706 | 0.908 | 3.207 | 1.659 | 0.097 |  |  |  |
| Kalo, 1996 | 1.678 | 0.908 | 3.102 | 1.650 | 0.099 |  |  |  |
| Karabay, 2004 | 1.749 | 0.928 | 3.297 | 1.729 | 0.084 |  |  |  |
| Alavi, 2014 | 1.614 | 0.869 | 2.996 | 1.516 | 0.129 |  |  |  |
| Lang, 1990 | 1.503 | 0.813 | 2.778 | 1.299 | 0.194 |  |  |  |
| Keramat, 2009 | 1.967 | 1.004 | 3.852 | 1.973 | 0.048 |  |  |  |
| Akova, 1993 | 1.678 | 0.909 | 3.100 | 1.653 | 0.098 |  |  |  |
| Bayindir, 2003 | 1.496 | 0.780 | 2.871 | 1.212 | 0.226 |  |  |  |
|  | 1.634 | 0.898 | 2.973 | 1.608 | 0.108 |  |  |  |
| **0.01 0.1 1** | | | | | | | **10** | **100** |
|  |  |  |  |  |  | FQ-Regimen **Standard-Regimen** | | |
| Fixed model |  |  |  |  |  |  | | |

|  | **Point** | **limit** | **limit** | **Z-Value** | **p-Value** |  | | |
| --- | --- | --- | --- | --- | --- | --- | --- | --- |
| Agalar, 1999 | 1.655 | 0.872 | 3.140 | 1.540 | 0.123  |  | | |
| Erfanian, 2011 | 1.399 | 0.747 | 2.618 | 1.050 | 0.294 |  | | |
| Saltoghlu, 2002 | 1.706 | 0.908 | 3.207 | 1.659 | 0.097 |  | | |
| Kalo, 1996 | 1.678 | 0.908 | 3.102 | 1.650 | 0.099 |  | | |
| Karabay, 2004 | 1.749 | 0.928 | 3.297 | 1.729 | 0.084 |  | | |
| Alavi, 2014 | 1.614 | 0.869 | 2.996 | 1.516 | 0.129 |  | | |
| Lang, 1990 | 1.503 | 0.813 | 2.778 | 1.299 | 0.194 |  | | |
| Keramat, 2009 | 1.967 | 1.004 | 3.852 | 1.973 | 0.048 |  | | |
| Akova, 1993 | 1.678 | 0.909 | 3.100 | 1.653 | 0.098 |  | | |
| Bayindir, 2003 | 1.496 | 0.780 | 2.871 | 1.212 | 0.226 |  | | |
|  | 1.634 | 0.898 | 2.973 | 1.608 | 0.108 |  | | |
| **0.01 0.1 1** | | | | | | | **10** | **100** |
|  |  |  |  |  |  | FQ-Regimen **Standard-Regimen** | | |
| Fixed model |  |  |  |  |  |  | | |

�G_ro_u-p�b�Y'-c------- **Study name	Statistics for each study Samplesizegroup	Risk Lower Upper**

**ratio limit	limit Z-Value p-Value**

**Risk ratio and 95'/, Cl**

| **50 patients or more** | Erfanian, 20118.217 | 1.090 61.954 | 2.044 | 0.041  |  |  |  |  |
| --- | --- | --- | --- | --- | --- | --- | --- | --- |
| **50 patients or more** | Saltoghlu, 2002.111 | 0.168 7.353 | 0.109 | 0.913 |  |  |  |  |
| **50 patients or more** | Alavi, 2014 1.963 | 0.183 21.006 | 0.558 | 0.577 |  |  |  |  |
| **50 patients or more** | Keramat, 20000.802 | 0.215 2.992 | -0.329 | 0.742 |  |  |  |  |
| **50 patients or more** | Akova, 1993 0.968 | 0.063 14.779 | -0.024 | 0.981 |  |  |  |  |
| **50 patients or more** | 1.459 | 0.629 3.382 | 0.880 | 0.379 |  |  |  |  |
| Less than 50 patients | Agalar, 1999 1.500 | 0.280 8.036 | 0.473 | 0.636 |  |  |  |  |
| **less than 50 patients** | Kalo, 1996 1.000 | 0.070 14.208 | 0.000 | 1.000 |  |  |  |  |
| Less than 50 patients | Karabay, 20040.933 | 0.151 5.758 | -0.074 | 0.941 |  |  |  |  |
| **Less than 50 patients** | Lang, 1990 7.857 | 0.551112.089 | 1.520 | 0.128 |  |  |  |  |
| Less than 50 patients | Bayindir, 20032.632 | 0.579 11.970 | 1.252 | 0.211 |  |  |  |  |
| Less than 50 patients | 1.836 | 0.783 4.307 | 1.398 | 0.162 |  |  |  |  |
| Overall | 1.634 | 0.898 2.973 | 1.608 | 0.108 |  |  |  |  |
|  |  |  |  |  | 0.01 | 0.1 | 10 | 100 |

FQ-Regimen Standard-Regimen

Random model

Adverse Event

| 6 weeks or more | Alikhani, 2007 0.270 | 0.112 | 0.653 | -2.908 | 0.004 |
| --- | --- | --- | --- | --- | --- |
| 6 weeks or more | Alavi, 2014 0.765 | 0.223 | 2.765 | -0.376 | 0.707 |
| 6 weeks or more | Keramat, 2009 1.091 | 0.453 | 2.630 | 0.195 | 0.845 |
| 6 weeks or more | Didgar, 2012 0.750 | 0.179 | 3.138 | -0.394 | 0.694 |
| 6 weeks or more | 0.604 | 0.359 | 1.016 | -1.899 | 0.058 |
| Less equal 8 weeks | Kalo, 1996 3.000 | 0.134 | 67.056 | 0.693 | 0.488 |
| Less equal 6 weeks | Karabey, 2004 0.700 | 0.189 | 2.589 | -0.535 | 0.593 |
| Less equal 6 week!:> | Ako11a, 1993 0.149 | 0.037 | 0.605 | -2.663 | 0.008 |

0.01	0.1	10	100

**Funnel Plot of Standard Error by Log odds ratio**

0.0

0.5

g

LU

�

1.0 -

,"E,

*s*

"'

,:

(/)

1.5

2.0

-4

.3

**·2**

·1

0

2

4

Log odds ratio

**Sub-group Analysis**

Group by

Intervention duration sub-group

Stucty name

Statistics for each stu�

Risk Lower Upper

ratio limit	limit Z-Value p-Value

-Risk ratio and 95% Cl

Less equal 6 weeks Less equal 6 weeks 011erall

Bayindir, 2003 7.350 0,405 133.476 1.348 0.178

0.534 0.223 1.275 -1.413 0.158

0.585 0.374 0.914 -2.355 Q.Q19

0

•

FQ-Rgimen	Standard-Regimen

Fixed model

c=G�,o�u�p�b~ye-----------	**Study name	Statistics for each study Intervention group	Odds	Lower	Upper**

**ratio limit	limit Z-Value p-Value**

| **Ciprofloxacin-based** | Kalo, 1996 3.261 | 0.120 | 88.347 | 0.702 | 0.483 |
| --- | --- | --- | --- | --- | --- |
| **Ciprofloxacin-based** | Alavi, 2014 0.768 | 0.195 | 3.032 | -0.377 | 0.706 |
| **Ciprofloxacin-based** | Keramat, 20091.103 | 0.411 | 2.962 | 0.195 | 0.846 |
| **Ciprofloxacin-based** | Didgar, 2012 0.730 | 0.152 | 3.492 | -0.394 | 0.693 |
| **Ciprofloxacin-based** | 0.971 | 0.484 | 1.952 | -0.081 | 0.935 |
| **Ofloxacin-based** | Alikhani, 20070.160 | 0.051 | 0.500 | -3.151 | 0.002 |
| **Ofloxacin-based** | Karabey, 20040.625 | 0.112 | 3.477 | -0.537 | 0.591 |
| **Ofloxacin-based** | Akova, 1993 0.090 | 0.018 | 0.449 | -2.939 | 0.003 |
| **Ofloxacin-based** | Bayindir, 20038.697 | 0.419180.591 | | 1.398 | 0.162 |
| **Ofloxacin-based** | 0.349 | 0.082 1.485 | | -1.425 | 0.154 |
| Overall | 0.801 | 0.427 1.502 | | -0.692 | 0.489 |

**Odds ratio and 95°/4 Cl**

**0.01	0.1	10	100**

**FQ-Regimen Standard-Regimen**

**Random model**

Group by

Location

Study name	Statistics for each study	Odds ratio and 95% Cl

**Odds Lower Upper**

**ratio limit**	limit **Z-Value p-Value**

| Albania | Kalo, 1996 | 3.261 | 0.120 | 88.347 | 0.702 | 0.483  |  |  |  |  |
| --- | --- | --- | --- | --- | --- | --- | --- | --- | --- | --- |
| Albania |  | 3.261 | 0.120 | 88.347 | 0.702 | 0.483 |  |  |  |  |
| Iran | Alikhani, 2007 | 0.160 | 0.051 | 0.500 | -3.151 | 0.002 |  |  |  |  |
| Iran | Alavi, 2014 | 0.768 | 0.195 | 3.032 | -0.377 | 0.706 |  |  |  |  |
| Iran | Keramat, 2009 | 1.103 | 0.411 | 2.962 | 0.195 | 0.846 |  |  |  |  |
| Iran | Didgar, 2012 | 0.730 | 0.152 | 3.492 | -0.394 | 0.693 |  |  |  |  |
| Iran |  | 0.554 | 0.218 | 1.409 | -1.240 | 0.215 |  |  |  |  |
| Turkey | Karabey, 2004 | 0.625 | 0.112 | 3.477 | -0.537 | 0.591 |  |  |  |  |
| Turkey | Akova, 1993 | 0.090 | 0.018 | 0.449 | -2.939 | 0.003 |  |  |  |  |
| Turkey | Bayindir, 2003 | 8.697 | 0.419 | 180.591 | 1.398 | 0.162 |  |  |  |  |
| Turkey |  | 0.586 | 0.061 | 5.594 | -0.465 | 0.642 |  |  |  |  |
| Overall |  | 0.626 | 0.272 | 1.440 | -1.103 | 0.270 |  |  |  |  |
|  |  |  |  |  |  |  | **0.01** | **0.1** | **10** | **100** |

**FQ-Regimen Standard-Regimen**

**Random model**

| **Stud:t'n. ame** |  | **Statistics with stud:t'. removed** | | | | **Odds ratio (95% Cl)** | | | |
| --- | --- | --- | --- | --- | --- | --- | --- | --- | --- |
|  | **Point** | **Lower limit** | **Upper limit** | **Z-Value** | **p-Value** | **with study removed**  | | | |
| Alikhani, 2007 | 0.731 | 0.314 | 1.701 | -0.728 | 0.466 |  | | | |
| Kale, 1996 | 0.522 | 0.217 | 1.255 | -1.453 | 0.146 |  | | | |
| Karabey, 2004 | 0.580 | 0.219 | 1.539 | -1.094 | 0.274 |  | | | |
| Alavi, 2014 | 0.561 | 0.206 | 1.527 | -1.131 | 0.258 |  | | | |
| Keramat, 2009 | 0.502 | 0.191 | 1.322 | -1.395 | 0.163 |  | | | |
| Akova, 1993 | 0.723 | 0.325 | 1.609 | -0.795 | 0.427 |  | | | |
| Bayindir, 2003 | 0.481 | 0.213 | 1.084 | -1.766 | 0.077 |  | | | |
| Didgar, 2012 | 0.567 | 0.212 | 1.516 | -1.131 | 0.258 |  | | | |
|  | 0.574 | 0.245 | 1.344 | -1.279 | 0.201 |  | | | |
| **0.01** | | | | | | | **0.1 1** | **10** | **100** |
|  |  |  |  |  |  | **FQ-Regimen Standard-Regimen** | | | |
| Random model |  |  |  |  |  |  | | | |

| **Stud:t'n. ame** |  | **Statistics with stud:t'. removed** | | | | **Odds ratio (95% Cl)** | | | |
| --- | --- | --- | --- | --- | --- | --- | --- | --- | --- |
|  | **Point** | **Lower limit** | **Upper limit** | **Z-Value** | **p-Value** | **with study removed**  | | | |
| Alikhani, 2007 | 0.731 | 0.314 | 1.701 | -0.728 | 0.466 |  | | | |
| Kale, 1996 | 0.522 | 0.217 | 1.255 | -1.453 | 0.146 |  | | | |
| Karabey, 2004 | 0.580 | 0.219 | 1.539 | -1.094 | 0.274 |  | | | |
| Alavi, 2014 | 0.561 | 0.206 | 1.527 | -1.131 | 0.258 |  | | | |
| Keramat, 2009 | 0.502 | 0.191 | 1.322 | -1.395 | 0.163 |  | | | |
| Akova, 1993 | 0.723 | 0.325 | 1.609 | -0.795 | 0.427 |  | | | |
| Bayindir, 2003 | 0.481 | 0.213 | 1.084 | -1.766 | 0.077 |  | | | |
| Didgar, 2012 | 0.567 | 0.212 | 1.516 | -1.131 | 0.258 |  | | | |
|  | 0.574 | 0.245 | 1.344 | -1.279 | 0.201 |  | | | |
| **0.01** | | | | | | | **0.1 1** | **10** | **100** |
|  |  |  |  |  |  | **FQ-Regimen Standard-Regimen** | | | |
| Random model |  |  |  |  |  |  | | | |

**Grou�b:t	Study- name	Statistics for each study-**

**Sample size sub-group	Odds Lower Upper**

**ratio limit	limit Z-Value p-Value**

**Odds ratio and 95°/, Cl**

| **50 patients ormore** | Alikhani, 20070.160 | 0.051 | 0.500 | -3.151 | 0.002  | | | | |
| --- | --- | --- | --- | --- | --- | --- | --- | --- | --- |
| **50 patients ormore** | **Alavi,** 2014 0.768 | 0.195 | 3032 | -0.377 | 0.706 | | | | |
| **50 patients ormore** | Keramat, 20091.103 | 0.411 | 2.962 | 0.195 | 0.846 | | | | |
| **50 patients ormore** | Akova, 1993 0.090 | 0.018 | 0.449 | -2.939 | 0.003 | | | | |
| **50 patients ormore** | Didgar, 2012 0.730 | 0.152 | 3.492 | -0.394 | 0.693 | | | | |
| **50 patients ormore** | 0.407 | 0.154 | 1.076 | -1.812 | 0.070 | | | | |
| Less than 50 patients | Kalo, 1996 3.261 | 0.120 **88.347** | | 0.702 | 0.483 |  |  |  |  |
| less than 50 patients | Karabey, 20040.625 | 0.112 3.477 | | -0.537 | 0.591 |  |  |  |  |
| **Less than 50 patients** | Bayindir, 20038.697 | 0.419180.591 | | 1.398 | 0.162 |  |  |  |  |
| Less than 50 patients | 1.641 | 0.328 8.203 | | 0.603 | 0.547 |  |  |  |  |
| Overall | 0.591 | 0.257 1.358 | | -1.239 | 0.215 |  |  |  |  |
|  |  |  | |  |  | **0.01** | 0.1 | 10 | 100 |

FQ-Regimen Standard-Regimen

Random model

**Time to Defervescence**

| **Stud� name** |  |  | **Statistics for each stud�** | | |  |  |  | **Difference in means and 95% Cl**  | | |  |
| --- | --- | --- | --- | --- | --- | --- | --- | --- | --- | --- | --- | --- |
| **Difference Standard** | | |  | **Lower** Upper | |  |  |  |  |  |  |  |
|  | **in means** | **error** | **Variance** | **limit** | **limit** Z-Value p-Value | | |  |  |  |  |  |
| Agalar, 1999 | 1.070 | 0.499 | 0.249 | 0.092 | 2.048 | 2.144 | 0.032 |  |  |  |  |  |
| Karabay, 2004 | 1.300 | 0.437 | 0.191 | 0.444 | 2.156 | 2.978 | 0.003 |  |  |  |  |  |
| Alavi, 2014 | 0.300 | 0.408 | 0.167 | -0.500 | 1.100 | 0.735 | 0.462 |  |  |  |  |  |
| Lang, 1990 | 1.200 | 0.645 | 0.417 | -0.065 | 2.465 | 1.859 | 0.063 |  |  |  |  |  |
| Akova, 1993 | 1.200 | 0.814 | 0.663 | -0.395 | 2.795 | 1.474 | 0.140 |  |  |  |  |  |
|  | 0.919 | 0.228 | 0.052 | 0.471 | 1.366 | 4.022 | 0.000 |  |  |  |  |  |
|  |  |  |  |  |  |  |  | -3.00 | -1.50 | 0.00 | 1.50 | 3.00 |
|  |  |  |  |  |  |  |  |  | FQ-Regimen Standard-Regimen | | | |
| Fixed model |  |  |  |  |  |  |  |  |  |  |  |  |

Time to Defervescence

Group by

lo1ervenb0n dural!On group

Study name	Slalislics for each •�	Difference in means and 95% Cl

Difference Standard	L-• Uppe,

'""'

i

6 weeks 01 more

6 weeks 01 more Less equal6 weeks Len equal6weeks

Lesseql.Ja6weeks

Leue<p.Jal6Wttks Less equal6 weeks Overall

In means ,nM Valiance	limit Z•Vllue p-Value

Alavi. 2014	0.300	0.408 0.167 -0.500 1.100 0.735 0.462

0.300	0.408 0.167 -0.500 1.100 0.735 0.462

Agalar, 1999	1.070	0.499 0.249 0.092 2.048 2.144 0.032

Kanlbay, 2004	1.300	0.437 0.191 0.444 2.156 2.978 0.003

Lang, 1990	1.200	0.645 0.<117 -0,065 2.465 1.859 0.063

AkOVl, 1993	1200	0.814 0.663 -0,395 2.795 147<1 0.1-40

1.200	0.276 0.076 0.660 1.740 4.356 0.000

0.919	0.228 0.052 0.<171 1.366 4.022 0000

- 27.00	·13.50	0.00	13.50	27.00

**Favours A Favours B**

Random model

�	Statistics for Heh atu�

Intervention group

Difference In means and 95%Cl

Difference Standard	low.,r Upper

in means	Variance limit limit

Z.Value p-Value

| Ciprofloxacin-based | Agalar, 1999 | 1.070 | 0.499 | 0.249 | 0.092 | 2.048 | 2.144 | 0.032 |  |  |  | |
| --- | --- | --- | --- | --- | --- | --- | --- | --- | --- | --- | --- | --- |
| Ciprofloxacin-based | Alavi, 2014 | 0.300 | 0.408 | 0.167 | -0.500 | 1.100 | 0.735 | 0.462 |  |  |  |  |
| Ciprofloxacin-based |  | 0.609 | 0.316 | 0.100 | -0.011 | 1.228 | 1.926 | 0.054 |  |  |  |  |
| Ofloxacin-based | Karabay, 2004 | 1.300 | 0.437 | 0.191 | 0.444 | 2.156 | 2.978 | 0.003 |  |  |  |  |
| Ofloxacin-based | Lang, 1990 | 1.200 | 0.645 | 0.417 | -0.065 | 2.465 | 1.859 | 0.063 |  |  |  |  |
| Ofloxacin-based | Akova, 1993 | 1.200 | 0.814 | 0.663 | -0.395 | 2.795 | 1.474 | 0.140 |  |  |  |  |
| Oftoxacin-based |  | 1.257 | 0.330 | 0.109 | 0.610 | 1.905 | 3.605 | 0.000 |  |  |  |  |
| Overall |  | 0.919 | 0.228 | 0.052 | 0.471 | 1.366 | 4.022 | 0.000 |  |  |  |  |
|  |  |  |  |  |  |  |  |  | -3.00 | -1.50 0.00 | 1.00  | 3.00 |

FQ-Regimen Standard-Regimen

Fixed model

| Groue!?I | Sludyn•m• |  | | Statistics for each atu� | | |  |  | Oifferu1c• in means and 95%Cl  | | | |
| --- | --- | --- | --- | --- | --- | --- | --- | --- | --- | --- | --- | --- |
| Location |  |  | |  |  | |  |  |  | | | |
|  | Difference Standard | | | | Lower Upper | |  |  |  | | | |
|  |  | In means | | error Variance Bmit Umlt Z-Value p-Value | | | | |  | | | |
| Iran | Alavi, 2014 | 0.300 | | 0.408 | 0.167 -0.500 1.100 | | 0.735 | 0.462 |  | | | |
| Iran |  | 0.300 | | 0.408 | 0.167 -0.500 1.100 | | 0.735 | 0.462 |  | | | |
| Turkey | Aga1ar, 1999 | 1.070 | | 0.499 | 0.249 0.092 2.048 | | 2.144 | 0.032 |  | | | |
| Turkey | Karabay, 2004 | | 1.300 | 0.437 | 0.191 0.444 | 2.156 | 2.978 | 0.003 | | | | |
| Turkey | Lang, 1990 | 1.200 | | 0.645 | 0.417 -0.065 2.465 | | 1.859 | 0.063 |  | | | |
| Turt<.ey | Akova, 1993 | 1.200 | | 0.814 | 0.663 -0.395 2.795 | | 1.474 | 0.140 |  | | | |
| Tul1(ey | 1.200 | | | 0.276 | 0.076 0.660 | 1.740 | 4.356 | 0.000 | | | | |
| Overall | 0.919 | | | 0.228 | 0.052 0.471 | 1.366 | 4.022 | 0.000 | � | | | |
| -3.00 | | | | | | | | | | -1.50 **0.00** | '·" | 3.00 |
|  |  |  | |  |  | |  |  | FQ-Regimen Standard-Regimen | | | |
| Fixed model | |  | |  |  | |  |  |  | | | |

Groue�	Statistics for each atu!:!,:

Population group

Difrarence in meana and 95% Cl

Difference Standard	Lower Upper

in means	Variance limit limit Z-Value p-Value

| Hospitalized | Agalar, 1999 | 1.070 | 0.499 | 0.249 | 0.092 | 2.048 | 2.144 | 0.032 |  |  |  | |
| --- | --- | --- | --- | --- | --- | --- | --- | --- | --- | --- | --- | --- |
| Hospitalized | Karabay, 2004 | 1.300 | 0.437 | 0.191 | 0.444 | 2.156 | 2.978 | 0.003 |  |  |  |  |
| Hospitalized |  | 1.200 | 0.329 | 0.108 | 0.556 | 1.844 | 3.653 | 0.000 |  |  |  |  |
| Uncomphcated | Alavi, 2014 | 0.300 | 0.408 | 0.167 | -0.500 | 1.100 | 0.735 | 0.462 |  |  |  |  |
| Uncomplicated | Lang, 1990 | 1.200 | 0.645 | 0.417 | -0.065 | 2.465 | 1.859 | 0.063 |  |  |  |  |
| Uncomplicated | Akova, 1993 | 1.200 | 0.814 | 0.663 | -0.395 | 2.795 | 1.474 | 0.140 |  |  |  |  |
| Uncomplicated |  | 0.655 | 0.318 | 0.101 | 0.032 | 1.278 | 2.062 | 0.039 |  |  |  |  |
| Overall |  | 0.919 | 0.228 | 0.052 | 0.471 | 1.366 | 4.022 | 0.000 |  |  |  |  |
|  |  |  |  |  |  |  |  |  | -3.00 | -1.50 0.00 | 1.50  | 3.00 |

FQ-Regimen Standard-Regimen

Fixed model

**Studl name	Statistics for each studl	Risk ratio and 95% Cl Risk Lower	Upper**

**ratio	limit	limit	Z-Value p-Value**

| Agalar, 1999 | 1.500 | 0.280 | 8.036 | 0.473 | 0.636  |  | | | |
| --- | --- | --- | --- | --- | --- | --- | --- | --- | --- |
| Erfanian, 2011 | 8.217 | 1.090 | 61.954 | 2.044 | 0.041 |  |  |  |  |
| Saltoghlu, 2002 | 1.111 | 0.168 | 7.353 | 0.109 | 0.913 |  |  |  |  |
| Kalo, 1996 | 1.000 | 0.070 | 14.208 | 0.000 | 1.000 |  |  |  |  |
| Karabay, 2004 | 0.933 | 0.151 | 5.758 | -0.074 | 0.941 |  |  |  |  |
| Alavi, 2014 | 1.963 | 0.183 | 21.006 | 0.558 | 0.577 |  |  |  |  |
| Lang, 1990 | 7.857 | 0.551 | 112.089 | 1.520 | 0.128 |  |  |  |  |
| Keramat, 2009 | 0.802 | 0.215 | 2.992 | -0.329 | 0.742 |  |  |  |  |
| Akova, 1993 | 0.968 | 0.063 | 14.779 | -0.024 | 0.981 |  |  |  |  |
| Bayindir, 2003 | 2.632 | 0.579 | 11.970 | 1.252 | 0.211 |  |  |  |  |
|  | 1.634 | 0.898 | 2.973 | 1.608 | 0.108 |  |  |  |  |
|  |  |  |  |  |  | **0.01** | **0.1 1** | **10** | **100** |

**FQ-Regimen Standard-Regimen**

Random model

Study name	Statistics with study removed Standard	Lower Upper

Point	error	Variance limit	limit Z-Value p-Value

Difference in means **(95%**

Cl) **with study removed**

I

| Agalar, 1999 | 0.891 | 0.276 | 0.076 | 0.350 | 1.432 | 3.230 | 0.001 |
| --- | --- | --- | --- | --- | --- | --- | --- |
| Karabay, 2004 | 0.775 | 0.268 | 0.072 | 0.249 | 1.300 | 2.891 | 0.004 |
| Alavi, 2014 | 1200 | 0.276 | 0.076 | 0.660 | 1.740 | 4.356 | 0.000 |
| Lang, 1990 | 0.884 | 0.256 | 0.066 | 0.382 | 1.386 | 3.454 | 0.001 |
| Akova, 1993 | 0.902 | 0.252 | 0.064 | 0.407 | 1.396 | 3.575 | 0.000 |
|  | 0.919 | 0.228 | 0.052 | 0.471 | 1.366 | 4.022 | 0.000 |

-3.00	-1.50	0.00	1.50	3.00

FQ-Regimen Standard-Regimen

Fixed model

Study name	Statistics with study removed Standard	Lower Upper

Point	error	Variance limit	limit Z-Value p-Value

Difference in means **(95%**

Cl) **with study removed**

I

| Agalar, 1999 | 0.891 | 0.276 | 0.076 | 0.350 | 1.432 | 3.230 | 0.001 |
| --- | --- | --- | --- | --- | --- | --- | --- |
| Karabay, 2004 | 0.775 | 0.268 | 0.072 | 0.249 | 1.300 | 2.891 | 0.004 |
| Alavi, 2014 | 1200 | 0.276 | 0.076 | 0.660 | 1.740 | 4.356 | 0.000 |
| Lang, 1990 | 0.884 | 0.256 | 0.066 | 0.382 | 1.386 | 3.454 | 0.001 |
| Akova, 1993 | 0.902 | 0.252 | 0.064 | 0.407 | 1.396 | 3.575 | 0.000 |
|  | 0.919 | 0.228 | 0.052 | 0.471 | 1.366 | 4.022 | 0.000 |

-3.00	-1.50	0.00	1.50	3.00

FQ-Regimen Standard-Regimen

Fixed model

| Grou� |  |  | Statiatics for Heh atu� | | | |  |  |  | Difference In mHna and 95% Cl  | |  |
| --- | --- | --- | --- | --- | --- | --- | --- | --- | --- | --- | --- | --- |
| Sample size group |  |  |  |  |  |  |  |  |  |  |  |  |
|  | Difference Standard | | |  | Lower Upper | |  |  |  |  |  |  |
|  | inmeana | |  | Variance | limit | limit Z-Vatue p-Value | | |  |  |  |  |
| 50 or more | Alavi, 2014 | 0.300 | 0.408 | 0.167 | -0.500 | 1.100 | 0.735 | 0.462 |  |  |  |  |
| 50 or more | Akova, 1993 | 1.200 | 0.814 | 0.663 | -0.395 | 2.795 | 1.474 | 0.140 |  |  |  |  |
| 50 or more |  | 0.481 | 0.365 | 0.133 | -0.234 | 1.196 | 1.318 | 0.188 |  |  |  |  |
| Less lhan 50 | Agalar, 1999 | 1.070 | 0.499 | 0.249 | 0.092 | 2.048 | 2.144 | 0.032 |  |  |  |  |
| Less lhan 50 | Karabay, 2004 | 1.300 | 0.437 | 0.191 | 0.444 | 2.156 | 2.978 | 0.003 |  |  |  |  |
| Less lhan 50 | Lang, 1990 | 1.200 | 0.645 | 0.417 | -0.1)65 | 2.465 | 1.859 | 0.063 |  |  |  |  |
| Less lhan 50 |  | 1.200 | 0.293 | 0.086 | 0.626 | 1.774 | 4.099 | 0.000 |  |  |  |  |
| Oterall |  | 0.919 | 0.228 | 0.052 | 0.471 | 1.366 | 4.022 | 0.000 | ..... | -1.50 0.00 | 1.50 | 3.00 |
|  |  |  |  |  |  |  |  |  |  | FQ-Regimen Standard-Regimen | | |
| Fixed model |  |  |  |  |  |  |  |  |  |  |  |  |

**Study name	Statistics for each study Risk Lower	Upper**

**ratio	limit	limit	Z-Value p-Value**

Agalar, 1999	1.500	0.280	8.036	0.473	0.636

Erfanian, 2011 8.217	1 090 61.954	2.044	0.041

Kala, 1996	1.000	0.070 14.208	0.000	1.000

Karabay, 2004 0.933	0.151	5.758	-0.074	0.941

Alavi, 2014	2.290	0.625	8.389	1.251	0.211

Lang, 1990	7.857	0.551 112.089	1.520	0.128

Keramat, 2009 2.806	1.212	6.499	2.408	0.016

Akova, 1993	0.968	0.063 14.779	-0.024	0.981

Didgar, 2012	2.000	0.537	7.448	1.033	0.301

Bayindir, 2003 1.754	0.485	6.350	0.857	0.392

2.231	1.393	3.572	3.341	0.001

**Risk ratio and 95% Cl**

--

-

--

----

-

•

--

**0.01	0.1	10	100**

**FQ-Regimen Standard-Regimen**

Fixed model

**Stud� name	Statistics for each stud!!'.**

**Difference Standard	Lower Upper**

**in means	error	Variance limit	limit Z-Value p-Value**

**Difference in means and 95% Cl**

| Agalar, 1999 | 1.070 | 0.499 | 0.249 | 0.092 | 2 048 | 2.144 | 0.032 |  | | | | |
| --- | --- | --- | --- | --- | --- | --- | --- | --- | --- | --- | --- | --- |
| Karabay, 2004 | 1.300 | 0 437 | 0.191 | 0444 | 2.156 | 2 978 | 0.003 |  |  |  |  |  |
| Alavi, 2014 | 0.300 | 0.408 | 0.167 | -0.500 | 1.100 | 0.735 | 0.462 |  |  |  |  |  |
| Lang, 1990 | 1.200 | 0.645 | 0.417 | -0.065 | 2.465 | 1.859 | 0.063 |  |  |  |  |  |
| Al\ova, 1993 | 1.200 | 0.814 | 0.663 | -0.395 | 2.795 | 1.474 | 0.140 |  |  |  |  |  |
|  | 0.919 | 0.228 | 0.052 | 0.471 | 1.366 | 4.022 | 0.000 |  |  |  |  |  |
|  |  |  |  |  |  |  |  | **-3.00** | **-1.50** | **0.00**  | **1.50** | **3.00** |

**FQ-Regimen Standard-Regimen**

**Fixed model**

# Time to Defervescence

**Study:name	Statistics for each stud:r:	Difference** in **means** and **95% Cl**

|  | **Difference in means** | **Standard error** | **Variance** | **Lower limit** | **Upper limit** | **Z-Value** | **p-Value** |  | | | | | |
| --- | --- | --- | --- | --- | --- | --- | --- | --- | --- | --- | --- | --- | --- |
| Agalar, 1999 | 1.070 | 0.499 | 0.249 | 0.092 | 2.048 | 2.144 | 0.032 |  |  |  |  |  |  |
| Karabay, 2004 | 1.300 | 0.437 | 0.191 | 0.444 | 2.156 | 2.978 | 0.003 |  |  |  |  |  |  |
| Alavi, 2014 | 0.300 | 0.408 | 0.167 | -0.500 | 1.100 | 0.735 | 0.462 |  |  |  |  |  |  |
| Lang, 1990 | 1.200 | 0.645 | 0.417 | -0.065 | 2.465 | 1.859 | 0.063 |  |  |  |  |  |  |
| Akova, 1993 | 1.200 | 0.814 | 0.663 | -0.395 | 2.795 | 1.474 | 0.140 |  |  |  |  |  |  |
|  | 0.919 | 0.228 | 0.052 | 0.471 | 1.366 | 4.022 | 0.000 |  |  |  | �  |  |  |
|  |  |  |  |  |  |  |  | **-3.00** | **-1.50** | **0.00** |  | **1.50** | **3.00** |

**FQ-Regimen Standard-Regimen**

**Fixed model**

Table	[Click here to access/download;Table;Table.docx](https://www2.cloud.editorialmanager.com/pntd/download.aspx?id=1516197&guid=d929b205-4169-44a2-88e1-f06a8c60add3&scheme=1)[<https://www2.cloud.editorialmanager.com/pntd/download.aspx?id=1516197&guid=d929b205-4169-44a2-88e1-f06a8c60add3&scheme=1>](https://www2.cloud.editorialmanager.com/pntd/download.aspx?id=1516197&guid=d929b205-4169-44a2-88e1-f06a8c60add3&scheme=1)

**Table X. GRADE profile of fluoroquinolone-based regimens for brucellosis outcomes**

| Outcomes | Risk of bias | Inconsistency | Indirectness | Imprecision | Publication Bias | Number of intervention/con  trol | Effect size (95% CI) | Quality of evidence |
| --- | --- | --- | --- | --- | --- | --- | --- | --- |
| Therapeutic failure | Some concerns | No serious limitations | No serious limitations | No serious limitations | Possible | N=31/30 (10  studies) | RR = 2.23  (95% CI:  1.39–3.57) | Moderate (⊕⊕⊕○) |
| Relapse | Some concerns | Moderate limitations | No serious limitations | Serious (wide CI) | Not detected | N=31/30 (8  studies) | RR = 1.63  (95% CI:  0.89–2.97) | Low  (⊕⊕○○) |
| Adverse events | Low risk | No serious limitations | No serious limitations | Serious (small  sample) | Not detected | N=31/30 (6  studies) | RR = 0.57  (95% CI:  0.24–1.34) | Moderate (⊕⊕⊕○) |
| Time to defervescence | Some concerns | No serious limitations | No serious limitations | No serious limitations | Not detected | N=31/30 (5  studies) | MD = 0.92  days (95%  CI: 0.47–  1.36) | Moderate (⊕⊕⊕○) |

**Table 2. Subgroup analysis of fluoroquinolone regimens in brucellosis treatment**

| Variable | Duration | | Intervention Regimen | | Location | | | Sample size | | Follow-up | |
| --- | --- | --- | --- | --- | --- | --- | --- | --- | --- | --- | --- |
| **Therapeutic**  **Failure** | 6 weeks≥ | 6 weeks< | Ciprofloxacin | Ofloxacin | Iran | Turkey | Other  Country | 50> | 50≤ | 6 Months≥ | 6 Months< |
| Number of  comparisons | 7 | 3 | 7 | 3 | 4 | 4 | 2 | 5 | 5 | 3 | 6 |
| Risk Ratio | 1.93 | 2.48 | 2.59 | 1.35 | 2.7  5 | 1.38 | 2.79 | 2.19 | 2.24 | 1.57 | 2.27 |
| P value | 0.07 | 0.001 | 0.001 | 0.54 | 0.0  01 | 0.4 | 0.2 | 0.09 | 0.004 | 0.36 | 0.02 |
| I2 (%) | 0% | 0% | 0% | 0% | 0% | 0% | 13% | 0% | 0% | 0% | 0% |
| P-  heterogenity | 0.6 | 0.9 | 0.8 | 0.8 | 0.2 | 0.9 | 0.2 | 0.4 | 0.9 | 0.6 | 0.6 |
| **Relapse** | 6 weeks≥ | 6 weeks< | Ciprofloxacin | Ofloxacin | Iran | Turkey | Other  Country | 50> | 50≤ | 6 Months≥ | 6 Months< |
| Number of  comparisons | 6 | 4 | 6 | 4 | 3 | 5 | 2 | 5 | 5 | 3 | 6 |

| Risk Ratio | 2.01 | 1.35 | 1.78 | 1.44 | 2.0  0 | 1.45 | 2.79 | 1.83 | 1.45 | 1.17 | 2.4 |
| --- | --- | --- | --- | --- | --- | --- | --- | --- | --- | --- | --- |
| P value | 0.1 | 0.4 | 0.1 | 0.4 | 0.3 | 0.3 | 0.3 | 0.1 | 0.3 | 0.8 | 0.03 |
| I2 (%) | 0% | 0% | 75% | 0% | 44  % | 0% | 13.5% | 0% | 0% | 0% | 0% |
| P-  heterogenity | 0.5 | 0.6 | 0.4 | 0.8 | 0.1 | 0.9 | 0.2 | 0.7 | 0.4 | 0.8 | 0.6 |
| **Adverse**  **Event** | 6 weeks≥ | 6 weeks< | Ciprofloxacin | Ofloxacin | Iran | Turkey | Other  Country | 50> | 50≤ | 6 Months≥ | 6 Months< |
| Number of  comparisons | 5 | 3 | 4 | 4 | 4 | 3 | 1 | 3 | 5 | 4 | 2 |
| Risk Ratio | 1.08 | 0.56 | 0.97 | 0.24 | 0.5  8 | 0.55 | 3.26 | 1.64 | 0.4 | 0.39 | 0.24 |
| P value | 0.9 | 0.1 | 0.9 | 0.000 | 0.6 | 0.2 | 0.4 | 0.5 | 0.07 | 0.01 | 0.05 |
| I2 (%) | 77% | 40% | 0% | 64% | 55  % | 73% | 0% | 20 | 64% | 41.8% | 85.3% |
| P-  heterogenity | 0.01 | 0.1 | 0.8 | 0.03 | 0.0  8 | 0.02 | 1 | 0.2 | 0.02 | 0.1 | 0.009 |
| **Time to Defervescenc**  **e** | 6 weeks≥ | 6 weeks< | Ciprofloxacin | Ofloxacin | Iran | Turkey | Other Country | 50> | 50≤ | 6 Months≥ | 6 Months< |
| Number of  comparisons | 4 | 1 | 2 | 3 | 1 | 4 | 0 | 3 | 2 | 2 | 3 |
| Risk Ratio | 1.2 | 0.3 | 0.63 | 1.25 | 0.3 | 1.2 | - | 1.2 | 0.48 | 0.78 | 1.13 |
| P value | 0.000 | 0.4 | 0.09 | 0.000 | 0.4 | 0.000 | - | 0.00  0 | 0.1 | 0.1 | 0.001 |
| I2 (%) | 0% | 0% | 29% | 0% | 0% | 0% | - | 0% | 0% | 64% | 0% |
| P-  heterogenity | 0.9 | 1 | 0.2 | 0.9 | 1 | 0.9 | - | 0.9 | 0.3 | 0.09 | 0.9 |

**Table 3. Assessment of publication bias in the impact of fluoroquinolone on brucellosis**

|  | Corrected Effect size | | Begg’s rank correction test | | Egger’s linear regression test | | | | Fail  safe N test |
| --- | --- | --- | --- | --- | --- | --- | --- | --- | --- |
|  | VMD | 95%CI | Kendal  Tau | P | Intercept | 95%CI | T  value | P | N |
| Therapeutic  Failure | 2.23 | 1.39-3.57 | -0.22 | 0.37 | -0.26 | -1.79-1.25 | 0.4 | 0.69 | 10 |
| Relapse | 1.63 | 0.89-2.97 | 0.17 | 0.47 | 1.04 | -1.50-3.58 | 0.94 | 0.37 | 10 |
| Adverse event | 0.43 | 0.18-1.03 | 0.10 | 0.71 | 1.37 | -2.58-5.32 | 0.84 | 0.42 | 8 |
| Time to  Defervescence | 0.72 | 0.33-1.12 | 0.1 | 0.8 | 1.63 | -4.19-7.47 | 0.89 | 0.43 | 5 |

Table 1. Demographic characteristics of the included studies

| First author/  year | Location | Mean age years (I/C) | Study Design | Sample size  Total(I/C) | Female/Male  (I/C) | Intervention  Group | Control Group | Duration | Population |
| --- | --- | --- | --- | --- | --- | --- | --- | --- | --- |
| Murat Akova/1993 | Ankara/Turkey | (34.4±14.3)/(37.8±15.1) | Parallel RCT | 61(30/31) | (16/14)/(14/17) | Ofloxacin  (400 mg once daily) Rifampicin (600 mg once  daily) | Doxycycline (200 mg once daily)  Rifampicin (600 mg once daily) | 6 weeks | Adults with acute uncomplicated brucellosis |
| Keramat/2009 | Hamedan/Iran | 39.67/43.66 | Parallel RCT | 178(61/117) | 61/117 | Doxycycline (200 mg once daily) Rifampicin (600 mg once daily) | Ciprofloxacin (500 mg PO twice daily) Rifampicin (600 mg once daily)  OR  Ciprofloxacin (500 mg PO twice daily) Doxycycline (200 mg  once daily) | 8-12  weeks | Adults with acute uncomplicated brucellosis |
| Lang/1990 | Israel | Range (18-58) | Parallel RCT | 10(6/4) | (3/3)/(3/1) | Ciprofloxacin (500 mg PO  twice daily) | Doxycycline (200 mg once daily)  Rifampicin (600 mg once daily) | 6 weeks | Adults with acute uncomplicated brucellosis |

| Kalo/1996 | Tirana, Albania | Total (31.8±13.5) | Parallel RCT | 24(12/12) | 10/14 | Ciprofloxacin (500 mg PO  twice daily) Doxycycline (200 mg once  daily) | Doxycycline (200 mg once daily)  Rifampicin (600 mg once daily) | 6 weeks | Adults with acute uncomplicated brucellosis |
| --- | --- | --- | --- | --- | --- | --- | --- | --- | --- |
| Erfanian/2011 | Mashhad/Iran | (36.8±10.9)/(35.2±10) | Prospective observational comparative trial (not blinded  RCT) | 50(23/27) | (12/15)/(10/13) | Ciprofloxacin (500 mg PO  twice daily) Rifampicin (600 mg once  daily) | Doxycycline (200 mg once daily)  Rifampicin (600 mg once daily) | 6 weeks | Adults with acute uncomplicated brucellosis |
| Saltoglu/2002 | Adana, Turke | 36.8±11.3 | Prospective comparative (not blinded  RCT) | 57(27/30) | (21/6)/(22/8) | Ofloxacin  (400 mg once daily) | Doxycycline (200 mg once daily)  Rifampicin (600 mg  once daily) | 45 day | acute/chronic (ostearticular included) |

|  |  |  |  |  |  | Rifampicin (600 mg once  daily) |  |  |  |
| --- | --- | --- | --- | --- | --- | --- | --- | --- | --- |
| Didgar/2012 | Arak/Iran | (38.6±16.7)/(35.4±16.4) | Randomized controlled trial (triple blind) | 80(40/40) | 20/60 | Ciprofloxacin (500 mg PO  twice daily) Doxycycline | Doxycycline (200 mg once daily)  Rifampicin (600 mg once daily) | 8 weeks | Adults with acute uncomplicated brucellosis |

|  |  |  |  |  |  | (200 mg once daily) |  |  |  |
| --- | --- | --- | --- | --- | --- | --- | --- | --- | --- |
| Alavi/2014 | Ahvaz/Iran | (31.6±14.3)/(31.3±13.6) | Parallel RCT | 107(54/53) | (29/25)/(26/27) | Ciprofloxacin (500 mg PO  twice daily) Doxycycline (200 mg once daily) | Doxycycline (200 mg once daily)  Rifampicin (600 mg once daily) | 8 weeks | Adults with acute uncomplicated brucellosis |
| Alikhani/2007 | Tonekabon/Iran | Range (15-80) | Single blinded Parallel RCT | 78(38/40) | NR | Ofloxacin  (400 mg once daily) Rifampicin (600 mg once daily) Streptomycin (750–1000 mg  IM daily) | Doxycycline (200 mg once daily)  Rifampicin (600 mg once daily) Streptomycin (750–1000 mg IM daily) | 3 months | Adults with acute uncomplicated brucellosis |
| Agalar/1999 | Ankara/Turkey | (37.8±13.9)/(37.1±15.8) | Parallel RCT | 40(20/20) | (12/8)/(7/13) | Ciprofloxacin (500 mg PO  twice daily) Rifampicin (600 mg once  daily) | Doxycycline (200 mg once daily)  Rifampicin (600 mg once daily) | 45 days | Hospitalized brucellosis patients |

| Karabay/2004 | Duzce/Turkey | Range(18-60/24-61) | Parallel RCT | 29(15/14) | (2/13)/(3/11) | Ofloxacin  (400 mg once daily) Rifampicin (600 mg once  daily) | Doxycycline (200 mg once daily)  Rifampicin (600 mg once daily) | 45 days | Hospitalized brucellosis patients |
| --- | --- | --- | --- | --- | --- | --- | --- | --- | --- |
| Bayindir/2003 | Malatya/Turkey | Overall: 40.5 | Parallel RCT | 102(19/83) | 46/56 | Ofloxacin  (400 mg once daily) Rifampicin (600 mg once daily) | ST=20  (Streptomycin 1g IM  + Tetracycline 500 mg PO q6h) SD=21(Streptomycin 1g IM + Doxycycline 100 mg PO q12h) DR=20 (Doxycycline  100 mg PO q12h + Rifampicin 15 mg/kg  PO qd) SDR=22  (Streptomycin 1g IM  + Doxycycline 100 mg PO q12h +  Rifampicin 15 mg/kg  PO qd) | 45 days | Brucellar spondylitis |

Supporting Information

[Click here to access/download](https://www2.cloud.editorialmanager.com/pntd/download.aspx?id=1515604&guid=6340e514-1205-4041-a251-9f2effb97699&scheme=1)

[Supporting Information](https://www2.cloud.editorialmanager.com/pntd/download.aspx?id=1515604&guid=6340e514-1205-4041-a251-9f2effb97699&scheme=1)

[The role of ciprofloxacin in brucellosis treatment.docx](https://www2.cloud.editorialmanager.com/pntd/download.aspx?id=1515604&guid=6340e514-1205-4041-a251-9f2effb97699&scheme=1)
