# Supplementary material for: Efficacy and safety of fluoroquinolones in the treatment of human brucellosis: A systematic review and meta-analysis
Source: PLoS Negl Trop Dis. 2026 Oct 23;20(10):e0013822. doi: 10.1371/journal.pntd.0013822 (PMC13722316; doi:10.1371/journal.pntd.0013822)
Supplement: Supplementary file 9 [file pntd.0013822.s010.docx]

Response to Reviewers

Manuscript ID: PNTD-D-25-02141
Title: Efficacy and Safety of Fluoroquinolones in the Treatment of Human Brucellosis: A Systematic Review and Meta-Analysis

We sincerely thank the Editor and the Reviewers for their thoughtful and constructive comments. We carefully revised the manuscript and addressed all concerns in detail below. All modifications have been incorporated into the revised manuscript and highlighted accordingly.

**Reviewer #1**

Reviewer Comment:
The objectives are not clearly articulated with a testable hypothesis.

Response:

We thank the reviewer for this important comment. We have now explicitly stated a clear and testable hypothesis in the Introduction section, specifying that fluoroquinolone-based regimens were evaluated for their association with treatment failure and relapse compared with standard regimens in human brucellosis.

Reviewer Comment:
The search strategy lacks sufficient detail (keywords, databases, time frame).

Response:
We agree and have substantially expanded the Search Strategy section. The revised manuscript now includes complete details regarding all databases searched (PubMed, Scopus, Web of Science, and Cochrane Library), search dates, Medical Subject Headings (MeSH), free-text terms, Boolean operators, and database-specific syntax. The search period is now clearly reported as extending from database inception to August 2, 2025. We have substantially expanded the Search Strategy section, including full database names, search terms, Boolean operators, and date ranges in Supplementary Appendix 1.

Reviewer Comment:
PRISMA compliance requires full search strategy in supplement.

Response:
The full electronic search strategy for at least one major database has now been included in the supplementary materials in accordance with PRISMA 2020 guidelines.

Reviewer Comment:
Screening process not clearly described.

Response:
We have clarified that study selection was performed independently by two reviewers in a two-stage process (title/abstract screening followed by full-text review), with disagreements resolved through discussion and consensus. The PRISMA flow diagram has been updated accordingly.

Reviewer Comment:
Risk of Bias tool not specified.

Response:
We have explicitly stated that the Cochrane Risk of Bias 2 (RoB 2) tool was used for randomized controlled trials.

Reviewer Comment:
Detailed domain-level Risk of Bias table missing.

Response:
A comprehensive domain-level Risk of Bias assessment table has now been added to the supplementary materials. This table presents judgments for each RoB 2 domain (low risk, some concerns, or high risk of bias) across all included studies. A graphical summary has also been provided.

Reviewer Comment:
Statistical pooling model not clearly justified.

Response:
We appreciate this valuable comment. The Statistical Analysis section has been revised to clarify that the random-effects model (DerSimonian–Laird method) was prespecified as the primary analytical approach due to anticipated clinical and methodological heterogeneity among studies. Fixed-effect models were used only as sensitivity analyses.

Reviewer Comment:
Heterogeneity statistics not fully reported.

Response:
We now consistently report Cochran’s Q statistic, associated P-values, and I² statistics for all pooled analyses throughout the Results section.

Reviewer Comment:
Sensitivity analyses not described.

Response:
Sensitivity analyses using leave-one-out methods and alternative statistical models have now been fully described in the Results section. Importantly, pooled estimates remained stable after sequential exclusion of individual studies, supporting the robustness of the findings.

Reviewer Comment:
Subgroup conclusion about ciprofloxacin lacks quantitative support.

Response:
We have now performed additional subgroup analyses stratified by specific fluoroquinolone agents, including ciprofloxacin-, ofloxacin-, and levofloxacin-based regimens. Corresponding forest plots and subgroup interaction tests have been added to the revised manuscript and supplementary materials.

**Reviewer #2**

Reviewer Comment:
Study design does not fully align with objective; mixing RCTs and non-randomized studies.

Response:
We thank the reviewer for this important methodological observation. In the present systematic review, we included both randomized controlled trials and prospective comparative studies in order to comprehensively capture the available evidence on fluoroquinolone-based regimens in human brucellosis. We acknowledge that combining different study designs may introduce methodological heterogeneity and potential bias. To address this concern, we have clearly stratified study design characteristics in Table 1 and explicitly reported the study design of each included study throughout the Results section.

We have further clarified this issue in the Methods and Limitations sections, explicitly stating that although randomized trials represent the highest level of evidence, prospective comparative studies were also included due to the limited number of RCTs available in this field. This clarification ensures transparency while maintaining the comprehensiveness of the evidence base.

Reviewer Comment:
Acute and complicated cases pooled without stratification.

Response:

We have now performed subgroup analyses separating uncomplicated acute brucellosis from complicated forms, including osteoarticular disease. Results are reported separately in the Results section.

Reviewer Comment:
Effective power limited by small individual trials.

Response:

We acknowledge this limitation and have expanded the Discussion and Limitations sections to clarify that although meta-analysis increases overall power, small sample sizes at study level remain a limitation.

Reviewer Comment:
Inconsistent use of fixed and random-effects models.

Response:

All analyses now primarily use random-effects models, with fixed-effect models reported only as sensitivity analyses for comparison.

Reviewer Comment:
Continuous outcomes incorrectly reported using Risk Ratios.

Response:

We have corrected this issue. Continuous outcomes, including time to defervescence, are now reported as mean differences with 95% confidence intervals.

Reviewer Comment:
Results exceed certainty of evidence.

Response:

We have revised the Conclusions to align strictly with the GRADE assessment, ensuring that interpretations reflect low-to-moderate certainty of evidence.

Reviewer Comment:
Ofloxacin recommendation not sufficiently justified.

Response:
We have revised the wording to clarify that findings related to ofloxacin are exploratory and should be interpreted cautiously due to limited data.

**Reviewer #3**

Reviewer Comment:
The study gap is not convincingly justified given newer technologies (phage therapy, CRISPR, probiotics).

Response:
We have revised the Introduction and Discussion to better justify the relevance of evaluating fluoroquinolones in the context of current clinical practice. We also included discussion of emerging approaches such as phage therapy, CRISPR-based strategies, and probiotics, while clarifying that these remain experimental and not yet established in clinical practice.

Reviewer Comment:
Some parameters have small sample sizes.

Response:
We have explicitly acknowledged this limitation in the Limitations section and interpreted subgroup findings cautiously.

Reviewer Comment:
Discussion lacks coherence and contains fragmented paragraphs.

Response:
The Discussion section has been comprehensively reorganized and rewritten to improve logical flow, thematic consistency, and scientific clarity. Mechanistic explanations, clinical implications, and public health relevance are now integrated into more structured and cohesive paragraphs supported by updated references.

Reviewer Comment:
Old and inconsistent references; duplicated citations.

Response:
The reference list has been fully revised, duplicates removed, formatting standardized, and more recent literature added. We also clarify that older randomized trials remain the highest-quality evidence available, as recent studies are mostly observational and not suitable for quantitative synthesis.

**Editorial Comments (Reviewer #1 and Reviewer #2)**

Reviewer Comment:
Final number of studies and participants not clearly stated.

Response:
These have now been clearly reported in the Abstract and Results sections.

Reviewer Comment:
Statistical notation imprecise (e.g., P ≈ 0.11).

Response:
All statistical reporting has been standardized throughout the manuscript.

Reviewer Comment:
No dedicated Limitations section.

Response:
A separate section entitled “Limitations” has now been added immediately before the Conclusion section.

Reviewer Comment:
Tables duplicated, formatting inconsistent, labeling errors.

Response:
All tables and figures were carefully revised. Redundant tables were removed, formatting and font styles were standardized, labels corrected, and figure legends expanded to improve clarity and consistency.

Reviewer Comment:
Language and grammar require professional editing.

Response:
The manuscript underwent extensive language editing to improve grammar, readability, clarity, and scientific precision throughout the text.

We sincerely thank the Editor and Reviewers again for their valuable comments and constructive suggestions. We believe that the revised manuscript has been substantially strengthened and improved as a result of these revisions.

Sincerely,

Karo Servatyari
On behalf of all authors
