# Supplementary material for: Efficacy and safety of fluoroquinolones in the treatment of human brucellosis: A systematic review and meta-analysis
Source: PLoS Negl Trop Dis. 2026 Oct 23;20(10):e0013822. doi: 10.1371/journal.pntd.0013822 (PMC13722316; doi:10.1371/journal.pntd.0013822)
Supplement: Supplementary file 10 [file pntd.0013822.s011.docx]

Response to the Editor and Reviewers

**Dear Editor,**

Dear Editor,

We thank you and the reviewers for your thoughtful and thorough review of our manuscript entitled: “Efficacy and Safety of Fluoroquinolones in the Treatment of Human Brucellosis: A Systematic Review and Meta-analysis.” We appreciate your constructive criticism and helpful suggestions, which substantially contributed to improving the manuscript.

We also thank the reviewers for their excellent and completely correct comments. In particular, we are very grateful for the main suggestion to exclude non-RCTs from the analysis. As a result of re-evaluation of our inclusion criteria, as you correctly recommended, we have excluded all non-RCT studies from the analysis, which required us to completely reanalyze the data from the start. The statistical analysis and results have been revised accordingly.

We apologize for the delay with sending the revised version of the manuscript. Unfortunately, because of the current situation in Iran, we were not able to revise the manuscript as soon as we could. We hope that the above-mentioned information can justify the long time spent on revision and that it will be reflected in the quality of the revisions.

We carefully reviewed all the reviewer's comments and have made the relevant changes to the manuscript. Below all comments are listed in bold type, followed by our answer and the corresponding page and line numbers in the revised manuscript where the changes have been highlighted using Track Changes.

Thank you once again for your effort, patience and consideration.

"Please note that we used Gemini 3.1 Pro and Chat GPT plus, an AI tool, in the revision of this article."

Sincerely,
**Karo Servatyari, MD**
Corresponding Author
On behalf of all co-authors

| **No.** | **Reviewer Comment** | **Response** |
| --- | --- | --- |
| 1 | The manuscript is framed as a **non-inferiority** study; however, the statistical analysis evaluates superiority rather than non-inferiority. | Thank you for this insightful comment. We agree that our original wording implied a non-inferiority framework while the statistical analyses were conducted as comparative superiority analyses. We have revised the Introduction and Objectives accordingly and removed all references to non-inferiority. |
| 2 | The eligibility criteria state that only randomized controlled trials were included, whereas Table 1 also contains prospective comparative studies. | Thank you for raising this inconsistency. three non-RCT articles were removed from the analysis (Saltoglu 2002, Erfanian 2011, Alavi 2014), and the data were re-analyzed and corrected. |
| 3 | Several reported P values are inconsistent with the corresponding confidence intervals. | We appreciate this careful observation. All statistical analyses were rechecked against the original software outputs, and all discrepancies have been corrected. |
| 4 | The results for **time to defervescence** reported in the Abstract do not agree with those presented in the Results section. | We carefully reviewed this outcome and corrected the inconsistency. The Abstract, Results, Tables, and Discussion now consistently report the same pooled estimate. |
| 5 | Table 2 reports **Risk Ratios** for all outcomes, whereas continuous outcomes such as time to defervescence should be expressed as **Mean Difference**. | We thank the reviewer for this important point. We agree that continuous outcomes like time to defervescence should be reported as Mean Difference (MD), not Risk Ratio (RR). We have revised Table 2 accordingly and updated all related sections in the manuscript. |
| 6 | The manuscript inconsistently reports **Odds Ratios** and **Risk Ratios**. | Thank you for pointing out this inconsistency. We have now thoroughly revised the manuscript and standardized the reporting. **Risk Ratios (RR)** are now consistently reported for all variables, with the sole exception of 'time to defervescence,' which is a continuous outcome and is therefore reported as **Mean Difference (MD)**. All tables and corresponding sections have been updated accordingly. |
| 7 | Several confidence intervals appear mathematically implausible and should be verified. | We appreciate the reviewer's careful attention to this issue. Upon verification, we identified that the apparent inconsistencies in the confidence intervals were due to changes in the number of studies included across different analyses. We have now thoroughly rechecked and corrected all confidence intervals throughout the manuscript. All estimates are now consistent and accurately reflect the corresponding analyses. |
| 8 | The numbers reported in the PRISMA flow diagram are inconsistent. | We have carefully reviewed and corrected the PRISMA flow diagram. The numbers now reconcile completely, and a table of excluded studies has been added. |
| 9 | The reported **Fail-safe N** is relatively small and should be acknowledged as a limitation. | We have expanded the Limitations section to discuss the relatively small Fail-safe N and the potential impact on the robustness of the findings. |
| 10 | A statement suggesting the superiority of fluoroquinolones contradicts the pooled results. | We agree with the reviewer. The misleading statement has been removed to better reflect the findings of the meta-analysis.  **“Taken together, our findings do not support the superiority of fluoroquinolone-containing regimens over conventional doxycycline-based combinations. Although fluoroquinolone-containing regimens were associated with fewer adverse events, this potential tolerability advantage was accompanied by higher risks of treatment failure and relapse and a longer time to defervescence. Therefore, the observed safety advantage does not appear to outweigh the reduced treatment efficacy.”** |
| 11 | The Discussion contains numerous language problems and non-standard scientific expressions. | The Discussion has been extensively revised and professionally edited to improve scientific language and readability. |
| 12 | The country name **Ukraine** is misspelled. | Corrected as suggested. |
| 13 | The corresponding author's name is inconsistent between the title page and the response letter. | The corresponding author's name has been corrected and standardized throughout the submission. Dr. Karo Servatyari is the corresponding author |
| 14 | Some studies listed in Table 1 are missing from the reference list. | The missing references have been added to the reference list. bayindir et al was added to the reference list. |
| 15 | Several reference citations are incorrect or misnumbered. | All references and in-text citations have been carefully reviewed and corrected. |
| 16 | The method used to calculate **Fail-safe N** is not specified. | This text was added to limitation section. **“The relatively small fail-safe N values across the outcomes (N=5–10) represent an additional limitation, indicating that a relatively small number of unpublished null studies could potentially attenuate or overturn some of the observed associations. Therefore, the pooled estimates should be interpreted cautiously despite the absence of statistically significant findings from Begg’s and Egger’s tests.”** |
| 17 | The abbreviation **VMD** is unclear or non-standard. | The terminology has been revised using standard statistical nomenclature. **WMD: Weighted Mean Difference** |
| 18 | Table 4 does not explain the reasons for GRADE downgrading. | We have added a new column describing the reasons for each GRADE downgrade. |
| 19 | The **Data Availability Statement** does not comply with PLOS policy. | The dataset and supporting materials have now been deposited in a public repository, and the DOI has been included in the Data Availability Statement. |
| 20 | Supplementary Risk of Bias files are not cited in the manuscript. | Thank you for pointing this out. We have now cited the supplementary Risk of Bias file in the manuscript and added an appropriate cross-reference in the Results section under the “Risk of Bias Assessment” subsection. The supplementary file is now clearly identified as Supplementary FigureS1 and FigureS2. |
| 21 | The Discussion should better relate the findings to current clinical guidelines for brucellosis treatment. | We have expanded the Discussion to better interpret our findings in the context of current clinical guidelines and existing evidence. **“Our findings are consistent with current clinical practice recommendations, which continue to favor combination regimens based on doxycycline, most commonly with rifampicin or an aminoglycoside, for uncomplicated human brucellosis. Current guidance does not recommend fluoroquinolone-containing regimens as routine first-line therapy. The increased risks of treatment failure and relapse observed in our meta-analysis therefore provide clinical support for the continued use of established doxycycline-based combinations. Fluoroquinolones may nevertheless remain potential alternatives in selected patients when standard agents are contraindicated or poorly tolerated, although the evidence supporting their use in this setting remains limited.”** |
| 22 | Please include more recent references (2025–2026) where appropriate. | New and relevant studies have been added to the revised manuscript to the extent possible. |
| 23 | The Introduction requires a stronger background and rationale. | The Introduction has been substantially revised to better describe the background, research gap, objectives, and clinical significance of the study. “Despite favorable oral bioavailability, intracellular penetration, and tolerability, the clinical efficacy of fluoroquinolone-containing regimens for brucellosis remains uncertain. Previous studies have reported inconsistent findings and were often limited by small sample sizes and heterogeneous regimens. This research combines current findings related to the debated role of fluoroquinolones in the treatment of brucellosis, and therefore, we aimed to compare the efficacy and safety of fluoroquinolone-containing regimens with standard non-fluoroquinolone regimens for the treatment of human brucellosis.” |
| 24 | Please evaluate the manuscript for textual similarity. | The revised manuscript has been checked for textual similarity, and revisions have been made where appropriate. |
| 25 | If artificial intelligence tools were used, this should be disclosed. | Declaration of Generative Al and Al-Assisted Technologies in the Writing Process During the preparation of this manuscript, the authors used (Gemini 3.1 Pro and Chat GPT plus) solely to improve the grammar, language, and readability of the text. After using this tool, the authors reviewed and edited the content as needed and takes full responsibility for the content of the publication. |
| 26 | An **Author Summary** should be included. | An Author Summary has been prepared according to the journal's instructions. “Brucellosis is a zoonotic infection and treatment failure and relapse remain important issues. We evaluated fluoroquinolone regimens versus standard regimens for human brucellosis. Fluoroquinolone-based regimens had higher rates of treatment failure and relapse, and longer time to defervescence, but fewer adverse events. The results do not support the routine use of fluoroquinolones as first-line therapy. Standard doxycycline-based combination regimens should still be the mainstay of treatment.” |
| 27 | Figures should be uploaded in TIFF/EPS format. | All figures have been reformatted according to the journal's technical requirements. |
| 28 | Legends for the supplementary files are missing. | Legends have been added for all supplementary files. |
| 29 | Please provide a list of excluded studies and the reasons for exclusion. | A supplementary table listing all excluded studies and the reasons for exclusion has been added.  Several studies were ultimately excluded from our analysis, with the reason for each exclusion clearly specified. The studies by Erfanian and Saltoghlu were excluded because they were not randomized controlled trials (RCTs). The study by Alavi was excluded because the article was not published in English." |
| 30 | Please provide the extracted dataset to facilitate reproducibility. | The complete extracted dataset has been uploaded as supplementary material to facilitate transparency and reproducibility. |
