# Supplementary material for: Efficacy and safety of fluoroquinolones in the treatment of human brucellosis: A systematic review and meta-analysis
Source: PLoS Negl Trop Dis. 2026 Oct 23;20(10):e0013822. doi: 10.1371/journal.pntd.0013822 (PMC13722316; doi:10.1371/journal.pntd.0013822)
Supplement: Supplementary file 11 [file pntd.0013822.s012.docx]

Response to the Editor and Reviewers

**Dear Editor,**

Dear Editor,

We sincerely thank the Editor and the Reviewers for their careful, constructive, and detailed evaluation of our manuscript. We greatly appreciate the time and effort devoted to reviewing our work and the valuable comments and suggestions, which have helped us improve the clarity, methodological rigor, and overall quality of the manuscript.

In particular, following the Reviewer’s recommendation regarding the use of a random-effects model for the analysis of adverse events, we reanalyzed this outcome using a random-effects model instead of the previously applied fixed-effect model. With this approach, the association was no longer statistically significant (RR = 0.62, 95% CI: 0.29–1.33). We have therefore revised the interpretation of this outcome throughout the manuscript to accurately reflect the updated analysis. The corresponding results, tables, discussion, conclusions, and supplementary materials have been carefully reviewed and revised to ensure consistency with the final statistical analysis.

We have also addressed the other comments and recommendations provided by the Reviewers and have made the corresponding revisions throughout the manuscript. In addition, the requested Supporting Information files, including the study screening and exclusion table, detailed data-extraction table, risk-of-bias assessment, GRADE assessment, and extracted dataset, have been prepared and provided with the revised submission.

The present version represents the revised and updated manuscript incorporating all of the Reviewers’ comments and recommendations, together with the requested Supporting Information files. We hope that the revisions adequately address the concerns raised during the review process and have improved the quality and transparency of the manuscript.

We again sincerely thank the Editor and the Reviewers for their valuable comments and constructive guidance.

We have revised the Data Availability Statement and now provide the extracted dataset as S1 Dataset in the Supporting Information. No author is designated as a data-access contact point.

"Please note that we used Gemini 3.1 Pro and Chat GPT plus, an AI tool, in the revision of this article."

Sincerely,
**Karo Servatyari, MD**
Corresponding Author
On behalf of all co-authors

| **Reviewer / Journal Comment** | **Author Response and Correction** |
| --- | --- |
| **1. Author Summary** – Please provide an Author Summary (150–200 words) between the Abstract and the Introduction to make findings accessible to a wide audience. | Done. We have prepared an Author Summary of approximately 180 words and inserted it between the Abstract and the Introduction in the revised manuscript, following PLOS NTDs guidelines. |
| **2. Figure files** – Upload all main figures as separate files in .tif or .eps format. Use PLOS NAAS tool if needed. | Done. All main figures have been converted to high-resolution TIFF format meeting PLOS requirements (using the NAAS tool) and will be uploaded as separate files. Labelling errors have also been corrected. |
| **3. Supporting Information legends** – Add a full list of legends for Supporting Information files after the references list. | Done. A complete list of legends for all Supporting Information files has been added after the References section. |
| **4. Data Availability Statement** – Data must be freely available in a public repository, within the manuscript, or as supplementary information (not only “upon request”). | We have updated the Data Availability Statement. The extracted dataset, statistical code, and analysis outputs are now freely available [in a public repository / as Supporting Information – please specify]. |
| **5. Required data tables for systematic review** – Provide: (a) numbered table of all identified studies with exclusion reasons; (b) data-extraction table (extractors’ names, dates, eligibility, all data needed for replication); (c) risk-of-bias / GRADE assessments domain-by-domain; (d) explanation of missing-data handling. | **Response:** Thank you for this important comment. We have revised the Supporting Information and the Methods section to improve the transparency and reproducibility of the systematic review. **We have added a numbered study-screening table listing the identified studies and the reasons for exclusion, a detailed data-extraction table containing the study characteristics and outcome data required for replication, and domain-by-domain assessments of risk of bias and GRADE certainty for the included studies and outcomes.** We have also **clarified the procedures used to address missing or incompletely reported outcome data in the Methods section and documented any derived or estimated data in the extraction dataset.** The extracted dataset used for the quantitative synthesis is provided as S2 Dataset. |

| **Reviewer Comment** | **Author Response and Correction** |
| --- | --- |
| Adverse-event analysis still uses a fixed-effect model despite moderate heterogeneity. A random-effects sensitivity analysis should be added. | Thank you for this important comment. We agree that the presence of moderate heterogeneity (I² = 52%) warrants consideration of a random-effects model. We therefore repeated the adverse-event analysis using a random-effects model. The pooled effect estimate changed from a statistically significant result under the fixed-effect model to a non-statistically significant result under the random-effects model (RR = 0.63, 95% CI: 0.29–1.33).  Accordingly, we have revised the interpretation of this outcome throughout the manuscript and no longer state that fluoroquinolones significantly reduce adverse events. The results now indicate that **there was no statistically significant difference in adverse events between fluoroquinolone-containing regimens and comparator regimens under the random-effects model**. We have also revised the corresponding statements in the Abstract, Results, Discussion, and Conclusions to ensure consistency with the random-effects analysis. |
| PRISMA numbers are incorrect: text says 43 full texts assessed and 33 excluded, but exclusion categories add up to 34 (leaving 9 included studies). | Corrected. The text and PRISMA flowchart now consistently state: 43 full-text articles assessed → 34 excluded → 9 studies included. |
| Therapeutic failure P-value is inconsistent with the z statistic (z = 2.86 ≈ P = 0.004, but manuscript reports P < 0.001). Make uniform across abstract, results, tables and figures. | Corrected. The P-value has been revised to P = 0.004 throughout the Abstract, Results, tables and figure legends to match the z-statistic of 2.86. |
| Figure labelling errors: treatment-failure file labelled Figure 1 and time-to-defervescence labelled Figure 3, while manuscript refers to them as Figures 2 and 5. | Corrected. Files have been re-labelled: treatment failure = Figure 2; time to defervescence = Figure 5. In-text citations and legends are now consistent. |
| Method used to calculate fail-safe N is not named in the statistical analysis. Specify the procedure or remove the discussion. | Thank you for pointing this out. We apologize for the confusion. **Fail-safe N was not calculated or used in the statistical analysis of this meta-analysis. The mention of fail-safe N in the Discussion was included inadvertently. We have therefore removed all references to fail-safe N from the manuscript to avoid any ambiguity.** |
| Final language pass needed for awkward sentences in Discussion and Limitations (e.g., “fluoroquinolone marketing”, “intracellular time coating”). | Thank you for this helpful comment. We carefully reviewed the Discussion and Limitations sections for language, clarity, and academic style. Awkward and unclear expressions were revised while preserving the original scientific meaning. In particular, phrases such as “fluoroquinolone marketing” and “intracellular time coating” were replaced with more appropriate scientific wording. We also revised several choppy or translation-like sentences to improve readability and consistency throughout these sections. **The interpretation of the adverse-event outcome was also updated separately in response to the reviewer’s comment regarding the random-effects analysis.** |
| Methods cite PRISMA 2020 as ref. 9 and GRADE as ref. 11, but those numbers point to unrelated studies; several clinical trials appear twice. | Corrected. Reference list has been audited: correct PRISMA 2020 and GRADE citations restored and renumbered; duplicate trial entries removed; all in-text citations updated. |
| Corresponding author name should be “Karo Servatyari” but manuscript still says “Karo Servatyar”. | Corrected. The name has been standardized to “Karo Servatyari” throughout the manuscript. |
| Table 4 still describes the evidence setting as including prospective comparative studies, although synthesis is now restricted to RCTs. | Corrected. Table 4 now accurately states that the quantitative synthesis is restricted to randomized controlled trials only. |
| Overall: After correcting the above issues, the reviewer would be comfortable recommending acceptance. | We thank the reviewer for the positive assessment. All remaining points have been carefully addressed in the current revision. |
