# Supplementary material for: Efficacy and safety of fluoroquinolones in the treatment of human brucellosis: A systematic review and meta-analysis
Source: PLoS Negl Trop Dis. 2026 Oct 23;20(10):e0013822. doi: 10.1371/journal.pntd.0013822 (PMC13722316; doi:10.1371/journal.pntd.0013822)
Supplement: Peer review history

# Peer review: Decision Letter 0

**DOI:** 10.1371/journal.pntd.0013822.r001

**Authors:** Mathieu Picardeau, Mohammad Jokar

4 Jan 2026

Efficacy and Safety of Fluoroquinolones in the Treatment of Human Brucellosis: A Systematic Review and Meta-Analysis

PLOS Neglected Tropical Diseases

Dear Dr. servatyari,

Thank you for submitting your manuscript to PLOS Neglected Tropical Diseases. After careful consideration, we feel that it has merit but does not fully meet PLOS Neglected Tropical Diseases’s publication criteria as it currently stands. Therefore, we invite you to submit a revised version of the manuscript that addresses the points raised during the review process.

Please submit your revised manuscript within by Mar 05 2026 11:59PM. If you will need more time than this to complete your revisions, please reply to this message or contact the journal office at plosntds@plos.org. When you're ready to submit your revision, log on to https://www.editorialmanager.com/pntd/ and select the 'Submissions Needing Revision' folder to locate your manuscript file.

Please include the following items when submitting your revised manuscript:

* A letter that responds to each point raised by the editor and reviewer(s). You should upload this letter as a separate file labeled 'Response to Reviewers'. This file does not need to include responses to any formatting updates and technical items listed in the 'Journal Requirements' section below.

* A marked-up copy of your manuscript that highlights changes made to the original version. You should upload this as a separate file labeled 'Revised Manuscript with Track Changes'.

* An unmarked version of your revised paper without tracked changes. You should upload this as a separate file labeled 'Manuscript'.

If you would like to make changes to your financial disclosure, competing interests statement, or data availability statement, please make these updates within the submission form at the time of resubmission. Guidelines for resubmitting your figure files are available below the reviewer comments at the end of this letter.

We look forward to receiving your revised manuscript.

Kind regards,

Mohammad Jokar, DVM

Guest Editor

PLOS Neglected Tropical Diseases

Mathieu Picardeau

Section Editor

PLOS Neglected Tropical Diseases

Shaden Kamhawi

co-Editor-in-Chief

PLOS Neglected Tropical Diseases

orcid.org/0000-0003-4304-636XX

Paul Brindley

co-Editor-in-Chief

PLOS Neglected Tropical Diseases

orcid.org/0000-0003-1765-0002

**Journal Requirements:**

If the reviewer comments include a recommendation to cite specific previously published works, please review and evaluate these publications to determine whether they are relevant and should be cited. There is no requirement to cite these works unless the editor has indicated otherwise.

1) Please ensure that the CRediT author contributions listed for every co-author are completed accurately and in full.

At this stage, the following Authors/Authors require contributions: Mohsen Mohammadi-Sartang, Nastaran Rafieipour, Shahla Afrasiabian, Roya Mahmoudi, and Karo servatyari. Please ensure that the full contributions of each author are acknowledged in the "Add/Edit/Remove Authors" section of our submission form.

The list of CRediT author contributions may be found here: https://journals.plos.org/plosntds/s/authorship#loc-author-contributions

2) Please provide an Author Summary. This should appear in your manuscript between the Abstract (if applicable) and the Introduction, and should be 150-200 words long. The aim should be to make your findings accessible to a wide audience that includes both scientists and non-scientists. Sample summaries can be found on our website under Submission Guidelines:

https://journals.plos.org/plosntds/s/submission-guidelines#loc-parts-of-a-submission

3) Please upload all main figures as separate Figure files in .tif or .eps format. For more information about how to convert and format your figure files please see our guidelines:

https://journals.plos.org/plosntds/s/figures

4) We have noticed that you have uploaded Supporting Information files, but you have not included a list of legends. Please add a full list of legends for your Supporting Information files after the references list.

5) Please provide a complete Data Availability Statement in the submission form, ensuring you include all necessary access information or a reason for why you are unable to make your data freely accessible. If your research concerns only data provided within your submission, please write "All data are in the manuscript and/or supporting information files" as your Data Availability Statement.

6) As required by our policy on Data Availability, please ensure your manuscript or supplementary information includes the following:

- A numbered table of all studies identified in the literature search, including those that were excluded from the analyses.

- For every excluded study, the table should list the reason(s) for exclusion.

- If any of the included studies are unpublished, include a link (URL) to the primary source or detailed information about how the content can be accessed.

- A table of all data extracted from the primary research sources for the systematic review and/or meta-analysis. The table must include the following information for each study:

- Name of data extractors and date of data extraction

- Confirmation that the study was eligible to be included in the review.

- All data extracted from each study for the reported systematic review and/or meta-analysis that would be needed to replicate your analyses.

- If data or supporting information were obtained from another source (e.g. correspondence with the author of the original research article), please provide the source of data and dates on which the data/information were obtained by your research group.

- If applicable for your analysis, a table showing the completed risk of bias and quality/certainty assessments for each study or outcome.  Please ensure this is provided for each domain or parameter assessed. For example, if you used the Cochrane risk-of-bias tool for randomized trials, provide answers to each of the signalling questions for each study. If you used GRADE to assess certainty of evidence, provide judgements about each of the quality of evidence factor. This should be provided for each outcome.

- An explanation of how missing data were handled.

This information can be included in the main text, supplementary information, or relevant data repository. Please note that providing these underlying data is a requirement for publication in this journal, and if these data are not provided your manuscript might be rejected.

**Reviewers' Comments:**

Reviewer’s Responses to Questions

**Key Review Criteria Required for Acceptance?**

As you describe the new analyses required for acceptance, please consider the following:

**Methods**

-Are the objectives of the study clearly articulated with a clear testable hypothesis stated?

-Is the study design appropriate to address the stated objectives?

-Is the population clearly described and appropriate for the hypothesis being tested?

-Is the sample size sufficient to ensure adequate power to address the hypothesis being tested?

-Were correct statistical analysis used to support conclusions?

-Are there concerns about ethical or regulatory requirements being met?

Reviewer #1: 2.1. Search Strategy and Selection Process

The core reproducibility of a systematic review rests upon a transparent search strategy.

Currently, the manuscript fails to provide sufficient detail regarding how primary studies

were identified.

• Missing Details: The exact search keywords used across all databases (e.g.,

PubMed/MEDLINE, Embase, Cochrane Central Register of Controlled Trials), the

specific date ranges covered by the search (beyond simply stating the time

interval), and the precise inclusion/exclusion criteria applied during screening

must be explicitly detailed.

• PRISMA Compliance: As the manuscript states adherence to PRISMA 2020

guidelines, it is mandatory to include the full search strategy for at least one primary

database (e.g., PubMed) as a supplementary appendix. This allows readers and

reviewers to verify the comprehensiveness of the search.

• Screening Process Documentation: The process of study selection

(screening titles/abstracts followed by full-text review) needs rigorous description.

Specify how many reviewers independently screened the studies, how

disagreements were resolved (e.g., consensus, third reviewer), and provide the flow

of studies through the selection stages, ideally using the PRISMA 2020 flow

diagram structure.

2.2. Risk of Bias Assessment

The quality assessment of included studies is crucial for interpreting the overall weight

of evidence.

• Tool Specification: The manuscript must explicitly state which validated tool was

employed to assess the quality and risk of bias in the included Randomized

Controlled Trials (RCTs). Given that the focus is on RCTs, the Cochrane Risk of

Bias tool for Randomized Trials (RoB 2) is the expected standard. If non-RCTs

(e.g., observational studies) were included, the appropriate tool (e.g., ROBINS-I)

must be specified.

Summary of Findings: A summary of the risk of bias assessment is•

required. This should be presented in a detailed table showing the judgment

(Low, Some Concerns, High Risk) for each bias domain (e.g.,

randomization process, deviations from intended interventions, missing outcome

data, measurement of the outcome, selection of the reported result) for each

included study. This table should ideally be placed within the main results

section or as a key supplementary figure/table.

2.3. Statistical Analysis Clarification

Ambiguity surrounding the statistical pooling methods undermines the validity of the

synthesized estimates.

• Pooling Model Justification: The manuscript must clearly describe the statistical

model used to combine effect sizes. Specifically, state whether a Fixed-Effect

Model (e.g., Mantel-Haenszel or Inverse Variance) or a

Random-Effects Model (e.g., DerSimonian–Laird, Hartung-Knapp) was utilized,

and provide the justification for this choice based on heterogeneity

observed or expected clinical variance.

• Heterogeneity Interpretation: The statement "no heterogeneity ( I = 2 0% )"

requires immediate contextualization, especially when pooling data from potentially

diverse clinical settings and patient populations, even if only a small number of

studies were ultimately included. If I2 is indeed 0% across all outcomes, this should

be highlighted, but the statistical test for

-value) must be

heterogeneity (e.g., Chi-squared test results, including the

P provided. If heterogeneity was present but minimized in subsequent

analyses, this process must be documented.

• Sensitivity Analyses: Sensitivity analyses are a critical component of robust meta analysis, especially when dealing with few studies. The authors must detail any

sensitivity analyses performed (e.g., exclusion of the highest risk-of-bias study,

pooling using alternative models, assessing the impact of different definitions of

"treatment failure") and report their results.

Reviewer #2: The main problem preventing acceptance is that the study’s methods do not fully align with its stated objective of definitively evaluating fluoroquinolone efficacy in brucellosis, which weakens the testable hypothesis and the strength of the conclusions. While the objective is generally clear, it is not framed as a precise hypothesis, and the study design is compromised by mixing randomized controlled trials with nonrandomized prospective studies despite initially stating RCT-only inclusion, which introduces bias and reduces internal validity. The population is broadly appropriate but is insufficiently stratified, as acute uncomplicated and complicated cases are pooled, obscuring clinically meaningful differences. The total sample size appears large, but effective power is limited by small individual trials, subgroup fragmentation, and heterogeneity in regimens and follow-up. Statistical methods are mostly appropriate, but key errors such as inconsistent use of fixed versus random effects and incorrect effect metrics for continuous outcomes weaken confidence in the findings. Ethical and regulatory standards are met, but to resolve these issues the authors should clearly state a testable hypothesis, restrict analyses to true RCTs or justify inclusion of nonrandomized studies with separate analyses, stratify populations by disease severity, correct statistical inconsistencies, and realign conclusions with the actual certainty of evidence to ensure methodological coherence and credibility.

Reviewer #3: The gap of the study is not convincing; it emanated from a very old recommendation. Nothing has been touched regarding the current advancements (e.g., effort has been made, but the authors should have justified why and how the focus on those drugs is still more important than the recent and technological based experiments (e.g. 1. Phage Therapy: A Targeted Solution for Brucellosis. Lashtoo Aghaee B, Alikhani MY, Kazemi S, Ahmadyousefi M, van Leeuwen WB, Nasaj M.Vector Borne Zoonotic Dis. 2025 Dec;25(12):713-721. doi: 10.1177/15303667251367519. Epub 2025 Aug 11.PMID: 40788720 Review. 2. CRISPR technology for diagnosis and treatment of human brucellosis. Lau CH, Li X, Liang QL, Guo R, Ren X, Xu Z, Zhu Y, Lai Y, Liu G, Huang Y, Wu W, Zhu H, Chen J, Zhang X.J Microbiol Methods. 2026 Jan;240:107339. doi: 10.1016/j.mimet.2025.107339. Epub 2025 Nov 20.

3. Complementary Use of Probiotics and Their Antimicrobial Proteins in Brucellosis: A Comprehensive Review of Mechanisms and Clinical Benefits. Soozangar N, Faghfuri E, Gholizadeh P. Probiotics Antimicrob Proteins. 2025 Aug 27. doi: 10.1007/s12602-025-10731-y. Online ahead of print. PMID: 40864417 Review.

The sample size for some parameters are very small for some parameters

Reviewer #4: (No Response)

**********

**Results**

-Does the analysis presented match the analysis plan?

-Are the results clearly and completely presented?

-Are the figures (Tables, Images) of sufficient quality for clarity?

Reviewer #1: 2.4. Subgroup Analysis and Drug Efficacy

The conclusion regarding the differential efficacy based on the specific

fluoroquinolone used requires quantitative verification.

• Quantitative Data Presentation: The assertion that "most unfavorable results are

related to ciprofloxacin" cannot remain qualitative. Forest plots

displaying the pooled outcome measures (e.g., relative risk for treatment failure,

risk difference for adverse events) stratified by the specific FQ agent (e.g.,

Ciprofloxacin vs. Ofloxacin vs. Levofloxacin) must be included.

• Statistical Comparison: If such a stratification was performed, the statistical

test used to compare the effect sizes between the subgroups -value. (e.g.,

test for interaction) must be reported, including the P

Reviewer #2: The main problem in the Results section is that the analyses presented do not consistently match the prespecified analysis plan, which creates confusion and undermines clarity. Although the authors state that random-effects models are primary, fixed-effects results are repeatedly emphasized, and continuous outcomes such as time to defervescence are incorrectly reported using risk ratios in subgroup tables instead of mean differences, indicating deviation from the planned statistical approach. The results are generally complete but not always clearly presented, as key findings are repeated across text and tables with minor inconsistencies in effect sizes and terminology, making interpretation difficult for readers. The figures and tables contain valuable information but suffer from formatting issues, redundancy, and labeling errors, including duplicated tables, misaligned columns, and inconsistent outcome metrics, which reduce readability and precision. To resolve these issues, the authors should strictly adhere to the predefined analysis plan, report only the correct effect measures for each outcome, harmonize text and tables, remove redundancies, and professionally revise all tables and figures to ensure clarity, consistency, and transparent communication of results.

Reviewer #3: The result is ok, but the tables lack quality and clarity (the title and numbering are different or numbered differently, some are duplicated, and different font sizes are used).

Reviewer #4: (No Response)

**********

**Conclusions**

-Are the conclusions supported by the data presented?

-Are the limitations of analysis clearly described?

-Do the authors discuss how these data can be helpful to advance our understanding of the topic under study?

-Is public health relevance addressed?

Reviewer #1: The current conclusions are not fully supported due to significant methodological gaps and lack of quantitative evidence. Specifically:

Support of Data: The conclusions regarding the differential efficacy of specific fluoroquinolones (e.g., Ciprofloxacin) require quantitative verification through subgroup analysis and forest plots, which are currently missing.

Limitations: A dedicated and detailed "Limitations" section is mandatory to address issues such as heterogeneity in diagnostic criteria and short follow-up periods, which is currently absent.

Understanding the Topic: While the topic is clinically relevant, the discussion needs more depth to explain the mechanistic reasons for the observed clinical failures of FQ regimens compared to standard treatments.

Public Health Relevance: The authors should more concretely specify how these findings should alter current clinical guidelines for brucellosis treatment to improve public health outcomes.

Reviewer #2: The main problem in the Conclusions section is that the strength of the statements exceeds what the data can reliably support, particularly regarding clinical recommendations. While the conclusion that fluoroquinolone based regimens are associated with higher treatment failure is supported by the pooled data, the suggestion that ofloxacin may be used in selected cases is not firmly justified given the low to moderate certainty of evidence, small subgroup sizes, and exploratory nature of those analyses. The limitations are acknowledged but not fully emphasized, especially the age and quality of included trials, population heterogeneity, and the vulnerability of results to unpublished negative studies. The authors do contribute to advancing understanding by synthesizing historical clinical evidence and linking it to pharmacological and intracellular mechanisms, which helps explain why in vitro activity does not translate into clinical success. Public health relevance is addressed but could be clearer, as the findings reinforce existing treatment guidelines in endemic regions and caution against inappropriate antibiotic substitution, thereby supporting rational antimicrobial use and resistance prevention.

Reviewer #3: conclusion and limitation are written well

The authors didn't discuss how their data can be helpful to the readers' understanding of the topic. The way the discussion is written is not good. There are lots of fragmented ideas and many paragraphs with just one or two sentences, with no or few references cited; the flow of ideas is ruined. Moreover, very old references are used, the styles followed in the reference writing is very different, and some references are duplicated.

Reviewer #4: (No Response)

**********

**Editorial and Data Presentation Modifications?**

Use this section for editorial suggestions as well as relatively minor modifications of existing data that would enhance clarity. If the only modifications needed are minor and/or editorial, you may wish to recommend “Minor Revision” or “Accept”.

Reviewer #1: 3. Minor Comments

These points require attention for final polish and adherence to reporting

standards:

• Participant and Study Counts: The abstract and the main Results section must

clearly state the final number of included studies and the total number of

participants included in the primary analyses.

• Precision in Statistics: Where statistical values are reported, authors must use

precise notation. For example, replace informal notations like P ≈ 0.11 with the

standard format: P = 0.11 . This applies to all reported P-values, confidence

intervals (CIs), and heterogeneity statistics.

• Dedicated Limitations Section: As noted above, a distinct, titled section labeled

Limitations must precede the Conclusion section to ensure it is not overlooked.

• Professional Editing: Given the recurring grammatical issues, professional copy editing, ideally by a native English speaker specializing in medical manuscripts, is

strongly recommended before resubmission to ensure the language accurately

reflects the scientific rigor of the analysis.

Reviewer #2: The main problem is that editorial and data presentation issues substantially reduce clarity despite the underlying analyses being largely complete. The manuscript contains repeated tables and figures, inconsistent terminology for effect measures, formatting errors within tables, and grammatical issues that interrupt readability and professional presentation. Data are sometimes duplicated across sections without added interpretive value, and figure legends do not always fully explain what is being displayed, which may confuse readers. These issues can be resolved by removing redundant tables, standardizing statistical terms across text and figures, correcting table structure and labels, and performing thorough language and formatting editing. Addressing these points would markedly improve transparency and reader comprehension, but because these editorial issues coexist with methodological concerns identified elsewhere, a recommendation of Major Revision rather than Minor Revision is appropriate at this stage.

Reviewer #3: (No Response)

Reviewer #4: (No Response)

**********

**Summary and General Comments**

Use this section to provide overall comments, discuss strengths/weaknesses of the study, novelty, significance, general execution and scholarship. You may also include additional comments for the author, including concerns about dual publication, research ethics, or publication ethics. If requesting major revision, please articulate the new experiments that are needed.

Reviewer #1: 1. General Comments

This manuscript reviews the efficacy and safety of fluoroquinolones (FQs) in the

treatment of human brucellosis through a systematic review and meta-analysis. The topic

is clinically relevant, particularly given ongoing debates on the optimal antibiotic

regimen for this zoonotic disease. The study is registered in

PROSPERO and reportedly follows PRISMA 2020 guidelines, both of which are

commendable steps toward transparent reporting.

However, the paper currently lacks essential methodological details and contains

interpretational issues that significantly limit the reliability and reproducibility of its

conclusions. The manuscript would benefit substantially from increased methodological

transparency, clearer and more detailed data presentation (especially statistical outputs),

and a significantly improved discussion quality that critically engages with the nuances of

the data synthesized.

Reviewer #2: This study addresses an important and clinically relevant question and brings together a large body of historical randomized evidence on fluoroquinolone use in human brucellosis, which is a clear strength and of value to clinicians and public health practitioners in endemic regions. The work is generally well grounded in existing literature, the scholarship is strong, and the mechanistic discussion thoughtfully connects clinical outcomes with intracellular pharmacology and resistance biology, adding conceptual depth. However, the novelty lies primarily in synthesis rather than new insight, and the overall execution is weakened by methodological inconsistencies, particularly the inclusion of nonrandomized studies despite an RCT focused objective, population heterogeneity, and statistical reporting inconsistencies that affect interpretability. There are no apparent concerns regarding research ethics, dual publication, or data integrity, but the strength of the conclusions currently exceeds the certainty of the evidence. Because of these issues, major revision is required, including redefinition of a clear testable hypothesis, restriction or stratification of analyses to appropriate study designs and patient populations, correction and alignment of statistical methods with the prespecified plan, and moderation of conclusions to match evidence strength. No new laboratory experiments are needed, but reanalysis of existing data with stricter methodological consistency is essential to support publication.

Reviewer #3: It is not novel and does not contribute that much to the existing knowledge. The author relied on old recommendations to make the review, had no strong justification regarding the need to make the review, as compared to the very few recent reports in the related topic or related areas, and mentioned nothing about the recent advancement efforts in treating the disease. Very few data are used to make comparisons and draw conclusions.

Reviewer #4: Thank you for the invitation to review this manuscript. I apologize for the delayed response. Please find my detailed comments and suggestions below.

Regarding the study itself, while it presents a relatively systematic meta-analysis on the use of fluoroquinolones—important therapeutic agents for brucellosis—the findings ultimately remain highly subjective. Significant gaps persist in our understanding of brucellosis treatment and drug resistance. It should be noted that in vitro drug susceptibility data cannot fully replicate in vivo pharmacokinetics, and substantial individual variability exists. Consequently, there is a clear need for long-term, scientifically rigorous cohort evaluations.

Specific comments on the manuscript are as follows:

Introduction: The background section currently conflates information from both animal and human studies. To sharpen the focus, the introduction should be revised to concentrate more directly on the relevant clinical context, unless the therapeutic use in animals is a stated objective of the meta-analysis.

Results: The authors have performed extensive statistical analyses. These results require cautious interpretation and should be presented with clear caveats to prevent potential misunderstanding by readers.

Discussion: This section requires significant strengthening. A thorough discussion dedicated to the inherent biases and limitations of this investigation is essential. Explicitly addressing these aspects will substantially enhance the academic rigor and translational value of the work.

I hope these comments are helpful for revising the manuscript.

**********

PLOS authors have the option to publish the peer review history of their article (what does this mean?). If published, this will include your full peer review and any attached files.

If you choose “no”, your identity will remain anonymous but your review may still be made public.

**Do you want your identity to be public for this peer review?** For information about this choice, including consent withdrawal, please see our Privacy Policy.

Reviewer #1: **Yes:**  Marzieh Jalalifar

Reviewer #2: **Yes:**  Dr.Arman Abdous DVM,MPH

Reviewer #3: No

Reviewer #4: **Yes:**  Zhiguo Liu

[NOTE: If reviewer comments were submitted as an attachment file, they will be attached to this email and accessible via the submission site. Please log into your account, locate the manuscript record, and check for the action link "View Attachments". If this link does not appear, there are no attachment files.]

**Figure resubmission:**

While revising your submission, we strongly recommend that you use PLOS’s NAAS tool (https://ngplosjournals.pagemajik.ai/artanalysis) to test your figure files. NAAS can convert your figure files to the TIFF file type and meet basic requirements (such as print size, resolution), or provide you with a report on issues that do not meet our requirements and that NAAS cannot fix.

**Reproducibility:**

To enhance the reproducibility of your results, we recommend that authors of applicable studies deposit laboratory protocols in protocols.io, where a protocol can be assigned its own identifier (DOI) such that it can be cited independently in the future. Additionally, PLOS ONE offers an option to publish peer-reviewed clinical study protocols. Read more information on sharing protocols at https://plos.org/protocols?utm_medium=editorial-email&utm_source=authorletters&utm_campaign=protocols

Attachment Submitted filename: PNTD-D-25-02141 for PLOS ONE commented.docx

Attachment Submitted filename: Comment on the paper.pdf

# Author comment: Author response to Decision Letter 1

**DOI:** 10.1371/journal.pntd.0013822.r002

25 Jun 2026

Attachment Submitted filename: Response to Reviewer.docx

# Peer review: Decision Letter 1

**DOI:** 10.1371/journal.pntd.0013822.r003

**Authors:** Mathieu Picardeau, Mohammad Jokar

22 Jul 2026

PLOS Neglected Tropical Diseases

Thank you for submitting your manuscript to PLOS Neglected Tropical Diseases. After careful consideration, we feel that it has merit but does not fully meet PLOS Neglected Tropical Diseases’s publication criteria as it currently stands. Therefore, we invite you to submit a revised version of the manuscript that addresses the points raised during the review process.

Response to Reviewers Revised Manuscript with Track Changes Manuscript

Mohammad Jokar, DVM

Shaden Kamhawi

co-Editor-in-Chief

PLOS Neglected Tropical Diseases

orcid.org/0000-0003-4304-636XX

Paul Brindley

co-Editor-in-Chief

PLOS Neglected Tropical Diseases

orcid.org/0000-0003-1765-0002

**Additional Editor Comments (if provided):**

**Journal Requirements:**

1) Please provide an Author Summary. This should appear in your manuscript between the Abstract (if applicable) and the Introduction, and should be 150-200 words long. The aim should be to make your findings accessible to a wide audience that includes both scientists and non-scientists. Sample summaries can be found on our website under Submission Guidelines:

https://journals.plos.org/plosntds/s/submission-guidelines#loc-parts-of-a-submission

2) Please upload all main figures as separate Figure files in .tif or .eps format. For more information about how to convert and format your figure files please see our guidelines:

https://journals.plos.org/plosntds/s/figures

3) We have noticed that you have cited Table  Table 2 in the manuscript file but there is no corresponding table in the manuscript.  Please amend your manuscript to include this table noting that tables should not be uploaded as individual files.

4) We have noticed that you have uploaded Supporting Information files, but you have not included a list of legends. Please add a full list of legends for your Supporting Information files after the references list.

5) In the online submission form, you indicated that All data underlying the findings of this systematic review and meta-analysis are derived from previously published studies and are fully available within the original articles included in the review. No new primary data were generated or collected for this study. The extracted dataset used for quantitative synthesis, along with statistical code and analysis outputs, can be made available from the corresponding author upon reasonable request. All source studies are publicly accessible through bibliographic databases including PubMed, Scopus, Web of Science, and the Cochrane Library.. All PLOS journals now require all data underlying the findings described in their manuscript to be freely available to other researchers, either

1. In a public repository

2. Within the manuscript itself

3. Uploaded as supplementary information.

This policy applies to all data except where public deposition would breach compliance with the protocol approved by your research ethics board. If your data cannot be made publicly available for ethical or legal reasons (e.g., public availability would compromise patient privacy), please explain your reasons by return email and your exemption request will be escalated to the editor for approval. Your exemption request will be handled independently and will not hold up the peer review process, but will need to be resolved should your manuscript be accepted for publication. One of the Editorial team will then be in touch if there are any issues.

6) As required by our policy on Data Availability, please ensure your manuscript or supplementary information includes the following:

- A numbered table of all studies identified in the literature search, including those that were excluded from the analyses.

- For every excluded study, the table should list the reason(s) for exclusion.

- If any of the included studies are unpublished, include a link (URL) to the primary source or detailed information about how the content can be accessed.

- A table of all data extracted from the primary research sources for the systematic review and/or meta-analysis. The table must include the following information for each study:

- Name of data extractors and date of data extraction

- Confirmation that the study was eligible to be included in the review.

- All data extracted from each study for the reported systematic review and/or meta-analysis that would be needed to replicate your analyses.

- If data or supporting information were obtained from another source (e.g. correspondence with the author of the original research article), please provide the source of data and dates on which the data/information were obtained by your research group.

- If applicable for your analysis, a table showing the completed risk of bias and quality/certainty assessments for each study or outcome.  Please ensure this is provided for each domain or parameter assessed. For example, if you used the Cochrane risk-of-bias tool for randomized trials, provide answers to each of the signalling questions for each study. If you used GRADE to assess certainty of evidence, provide judgements about each of the quality of evidence factor. This should be provided for each outcome.

- An explanation of how missing data were handled.

This information can be included in the main text, supplementary information, or relevant data repository. Please note that providing these underlying data is a requirement for publication in this journal, and if these data are not provided your manuscript might be rejected.

**Reviewers' comments:**

**Key Review Criteria Required for Acceptance?**

As you describe the new analyses required for acceptance, please consider the following:

**Methods**

-Are the objectives of the study clearly articulated with a clear testable hypothesis stated?

-Is the study design appropriate to address the stated objectives?

-Is the population clearly described and appropriate for the hypothesis being tested?

-Is the sample size sufficient to ensure adequate power to address the hypothesis being tested?

-Were correct statistical analysis used to support conclusions?

-Are there concerns about ethical or regulatory requirements being met?

Reviewer #2: Revision 1 sharpens the clinical question nicely. The authors want to know whether fluoroquinolones can stand in for standard doxycycline regimens when treating human brucellosis, and that question genuinely matters at the bedside. The hypothesis now sits clearly at the end of the introduction, which is a real improvement over the original submission. However, Revision 1 still carries a small mismatch between the stated hypothesis and the statistical machinery. The introduction promises a test of non-inferiority, but the analysis runs standard superiority-style risk ratios and mean differences with no non-inferiority margin defined anywhere. I would simply reframe the hypothesis in the next revision as a comparative effectiveness question and let the analysis match the aim naturally. The study design is appropriate in spirit, but Revision 1 still describes the eligibility criteria inconsistently. The abstract and methods claim only randomized controlled trials were included, while Table 1, unchanged in this regard from the original submission, quietly admits prospective observational studies and trials explicitly labeled "not blinded." This is not a fatal flaw. The evidence in this field comes in imperfect containers, and the authors of Revision 1 seem aware of that. The fix is honesty. In the next round, state plainly that randomized and prospective comparative studies were included, then consider a sensitivity analysis restricted to the true RCTs to address Reviewer 2’s concern directly. The population of adults with acute brucellosis across Iran, Turkey, Israel, and Albania is exactly the right group to study, and Revision 1 describes them adequately. The total of 816 patients remains modest, and Revision 1 now acknowledges this in a dedicated limitations section, which is a welcome addition. The statistical toolbox in Revision 1 is appropriate: random-effects models, heterogeneity testing, leave-one-out sensitivity, publication bias assessment, and GRADE. The problem is not the tools chosen for Revision 1 but some of the numbers coming out of them. Several P-values printed in the Revision 1 abstract do not match what the confidence intervals predict, and one cell in Table 2 has a point estimate sitting outside its own confidence interval. These are fixable calculation or data-entry errors that slipped through the Revision 1 process. Ethical requirements remain well met through the PROSPERO registration and exclusive use of previously published data.

Reviewer #5: -

**********

**Results**

-Does the analysis presented match the analysis plan?

-Are the results clearly and completely presented?

-Are the figures (Tables, Images) of sufficient quality for clarity?

Reviewer #2: The results in Revision 1 follow a trail that has a few spots where the footprints suddenly reverse direction. The most puzzling moment in this Revision 1 manuscript involves time to defervescence. The abstract tells readers that fluoroquinolone patients had a shorter time to fever resolution. Then the detailed results and Table 4, both part of the Revision 1 submission, report a mean difference of plus 0.93 days, meaning these patients actually took longer to cool down. These two stories cannot coexist in the same paper. One must be corrected in the next revision so the abstract and the body of the paper speak with one voice. The primary outcomes of therapeutic failure and relapse are reported clearly in Revision 1, with pooled risk ratios that paint a consistent picture of fluoroquinolones underperforming. The heterogeneity is low, and Revision 1 now includes leave-one-out sensitivity analyses that confirm no single study is driving the results. These are strengths of this revision. Table 2 in Revision 1 is where clarity starts to fray. The column header applies "RR (95% CIs)" to every outcome, but time to defervescence is a continuous measure and needs a "Mean Difference" header. Adverse events appear as odds ratios in the Revision 1 abstract and GRADE table, then suddenly become risk ratios in Revision 1 Table 2. One effect measure per outcome, carried consistently throughout the next version, would solve this. Some numbers in the Revision 1 Table 2 also raise a mathematical eyebrow. A risk ratio of 7.86 with bounds of 0.55 to 11.2 is not symmetric on the log scale. The same asymmetry haunts the relapse subgroup for ciprofloxacin in the same Revision 1 table. One adverse-event entry has a point estimate of 0.27 sitting below its own lower confidence limit of 0.38. These are data-entry imps that need catching before the next submission. The PRISMA flow in Revision 1 still has an arithmetic hole. The numbers sum to 221 excluded records, but 231 minus 12 included should equal 219. Ten studies are unaccounted for in this Revision 1 version. A quick audit of the screening log will patch this. The fail-safe N values are reported honestly in Revision 1, which I appreciate, but a value of 5 for time to defervescence is very small and means a handful of unpublished null studies could flip the result. The next revision should say that plainly rather than wrapping it in cautious language.

Reviewer #5: -

**********

**Conclusions**

-Are the conclusions supported by the data presented?

-Are the limitations of analysis clearly described?

-Do the authors discuss how these data can be helpful to advance our understanding of the topic under study?

-Is public health relevance addressed?

Reviewer #2: The headline conclusion in Revision 1 is clear and well supported by the data. Fluoroquinolone-based regimens carry roughly double the risk of therapeutic failure and relapse compared with standard therapy. That is a clinically meaningful signal, and the Revision 1 authors are right to recommend against routine first-line use. But there is a sentence lurking in the Revision 1 discussion, just before the conclusion paragraph, that says "preliminary data support the potential superiority of fluoroquinolone-containing regimens over doxycycline-focused therapies with respect to treatment success and overall clinical outcomes." This sentence is the narrative twin of the time-to-defervescence reversal found elsewhere in Revision 1. It says the opposite of what the pooled estimates show. If it is a remnant from an earlier draft that survived into Revision 1, it needs gentle deletion in the next round. If it refers to a specific subgroup, it needs clear contextualization in the next round. Right now, in the Revision 1 manuscript, it sits like a contradictory echo, undermining reader trust. The limitations section is a genuine strength of this Revision 1 submission and was not present in the original manuscript. The Revision 1 authors talk about small sample sizes, the age of trials, geographic concentration, and clinical variability. What is missing from the Revision 1 limitations is an honest nod to the low fail-safe N values and the fragility they imply for some pooled estimates. The next revision should add this. The mechanistic discussion in Revision 1 about intracellular pharmacology and Brucella resistance strategies is interesting and connects clinical findings to bench science. But the language in this Revision 1 discussion section sometimes stumbles. Phrases like "ultra-modern in vitro pharmacokinetic statistics," "reduced affection," "choppy translation," and "fluorolone based remedy" feel like they lost meaning somewhere between the original draft and Revision 1. A fluent scientific editor could polish these Revision 1 sentences until they shine for the next submission. The public health relevance is present in Revision 1 but quiet. A sentence in the next revision linking these findings to current treatment guidelines would help clinicians in endemic regions understand exactly how this evidence should shift their prescribing.

Reviewer #5: -

**********

**Editorial and Data Presentation Modifications?**

Use this section for editorial suggestions as well as relatively minor modifications of existing data that would enhance clarity. If the only modifications needed are minor and/or editorial, you may wish to recommend “Minor Revision” or “Accept”.

Reviewer #2: The Revision 1 manuscript needs a language polish, especially in the discussion section, which still carries several expressions that block meaning despite the revision effort. Phrases like "ultra-modern in vitro pharmacokinetic statistics," "reduced affection," "no fluoroquinolones containing rutin," and "low fact of evidence" survived into Revision 1 and need attention before the next submission. A fluent scientific editor, ideally one familiar with infectious disease pharmacology, should coax the Revision 1 discussion into clarity. Beyond the prose, specific small repairs are needed in Revision 1. On the title page, "Ukrains" should be "Ukraine." The corresponding author signs the Revision 1 response letter as "Servatyari" but appears as "Servatyar" on the Revision 1 title page. These two names need to agree in the next version. The reference list in Revision 1 has gaps that were not caught during this round of revision. Two studies proudly present in Revision 1 Table 1, Alavi 2014 and Bayindir 2003, are completely missing from the Revision 1 bibliography. The study attributed to Erfanian 2011 in Revision 1 Table 1 matches Taghvaee et al. 2011 in the references. Author names must match the published record in the next submission. Citation numbering in Revision 1 is tangled in places. Reference 9 is used for PRISMA in the Revision 1 methods, but the actual reference 9 is the Cochrane Risk of Bias chapter. The GRADE methods in Revision 1 cite reference 11, but reference 11 is a brucellosis trial, not the GRADE working group paper. An afternoon of careful reference auditing before the next submission will fix this. Revision 1 Table 2 needs structural attention. Separate effect-measure headers by outcome type: Risk Ratio, Odds Ratio, or Mean Difference. Do not paint them all with the same brush as Revision 1 currently does. Correct the mathematically impossible confidence intervals in Revision 1 Table 2 and the point estimate that drifted outside its own bounds. Revision 1 Table 3 should name its fail-safe N method, Rosenthal or Orwin. The header "VMD" in Revision 1 Table 3 is cryptic and should be replaced with a standard term. Revision 1 Table 4 is well built but would benefit from a column naming the specific domains driving each downgrade. The PRISMA flow text in Revision 1 needs its arithmetic gap closed by listing all exclusion reasons so the numbers sum cleanly. The Data Availability statement in Revision 1 currently offers data upon reasonable request, but PLOS policy expects more. For the next revision, upload the dataset, code, and outputs to Figshare or Zenodo, obtain a DOI, and place that DOI in the statement. Finally, the supplementary Risk of Bias table that the Revision 1 response letter describes should be cross-referenced in the manuscript text of the next revision, and the file must actually be sitting in the submission package, not just promised.

Reviewer #5: -

**********

**Summary and General Comments**

Use this section to provide overall comments, discuss strengths/weaknesses of the study, novelty, significance, general execution and scholarship. You may also include additional comments for the author, including concerns about dual publication, research ethics, or publication ethics. If requesting major revision, please articulate the new experiments that are needed.

Reviewer #2: This Revision 1 manuscript sits at a lovely intersection of clinical curiosity and practical need. Somewhere in an endemic region, a physician is looking at a brucellosis patient who cannot tolerate doxycycline and genuinely wants to know whether reaching for a fluoroquinolone is reasonable or risky. This Revision 1 meta-analysis gathers twelve studies and 816 patients and runs the numbers. The answer that emerges is reasonably clear. Fluoroquinolones are associated with about double the risk of treatment failure and relapse. They may be better tolerated, but they do not clear the infection as reliably. This is useful clinical intelligence, and it deserves publication in a clean, trustworthy form. Revision 1 has made genuine progress over the original submission. The GRADE assessment is new and helpful. The limitations section is a welcome addition. The subgroup analyses add exploratory depth. The response letter shows the authors engaged seriously with the previous round of review. What stands between this Revision 1 manuscript and acceptance is not a lack of merit. It is a layer of internal contradictions, numerical gremlins, and language rough patches that survived the revision or were introduced during it. The Revision 1 abstract says one thing about time to defervescence and the Revision 1 results say the opposite. The Revision 1 methods claim randomized trials only while the Revision 1 table admits observational studies. The P-values printed in Revision 1 do not always match what the confidence intervals whisper to a careful reader. Revision 1 Table 2 has numbers that cannot be mathematically true and headers that blur the difference between risk ratios and mean differences. The Revision 1 reference list is missing two included studies and has citation tangles. The Revision 1 discussion contains genuine insight alongside phrases that read like they survived a rough translation and were not caught during this round of revision. None of these problems requires new data or fresh analyses. Every single one is fixable at the keyboard with care, honesty, and perhaps a pot of strong tea. That is why I am recommending minor revision with an optimistic heart. I see a publishable paper here. The bones are good. The question matters. The answer is clinically useful. If the authors of the next revision can align the abstract with the results, harmonize the effect-measure labels, correct the Table 2 numbers, complete and audit the references, strengthen the data availability statement, and give the discussion a professional scientific English polish, this manuscript can stand with confidence. I look forward to reading that next version. It will be a contribution that clinicians in brucellosis-endemic regions can actually use when they sit across from a patient and need to make a treatment decision that matters.

Reviewer #5: Q1: It is recommended to incorporate more recent references in 2026 to enhance the timeliness and relevance of the manuscript.

Q2: The introduction section should be revised to address the following elements comprehensively:

• Background and Context: Provide a concise yet informative overview of the research area, including current challenges and key issues.

• Research Gap and Significance: Clearly identify gaps in the existing literature and justify the importance of addressing them, highlighting the contribution of this study.

• Objectives and Scope: Explicitly state the research objectives and define the scope of the study, including a brief description of the adopted methodology.

• Structure of the Paper: Present a clear outline of the manuscript’s structure to guide the reader through the subsequent sections.

Q3: The overall similarity index with published sources should be maintained below 15%, with similarity to any single source not exceeding 1%. It is recommended to utilize plagiarism detection tools such as Turnitin or iThenticate to verify this requirement. Please revise the manuscript accordingly and provide the similarity report.

Q4: It is suggested to organize both the current study and prior research within a structured table in the Literature Review section. This categorization will facilitate a clearer identification and demonstration of the research gap.

Q5: Given the extensive use of mathematical formulations, all equations should be thoroughly re-examined to ensure accuracy in terms of indices, notation, and conceptual correctness.

Q6: All figures and tables should be carefully reviewed to ensure they are adequately described and properly integrated within the text. Each should include clear and informative explanations.

Q7: Please verify that all references are accurate, properly formatted, and free of duplication.

Q8: Please clarify whether any artificial intelligence tools were utilized in the preparation of the manuscript, either partially or in full. If applicable, kindly update the acknowledgments section to include the following details:

• The name(s) of the software used

• The specific stages of the manuscript preparation affected (e.g., data collection, writing, editing)

**********

PLOS authors have the option to publish the peer review history of their article (what does this mean?). If published, this will include your full peer review and any attached files.

If you choose “no”, your identity will remain anonymous but your review may still be made public.

**Do you want your identity to be public for this peer review?** For information about this choice, including consent withdrawal, please see our Privacy Policy.

Reviewer #2: **Yes:**  Dr.Arman Abdous DVM,MPH

Reviewer #5: No

**Figure resubmission:**

**Reproducibility:**

To enhance the reproducibility of your results, we recommend that authors of applicable studies deposit laboratory protocols in protocols.io, where a protocol can be assigned its own identifier (DOI) such that it can be cited independently in the future. Additionally, PLOS ONE offers an option to publish peer-reviewed clinical study protocols. Read more information on sharing protocols at https://plos.org/protocols?utm_medium=editorial-email&utm_source=authorletters&utm_campaign=protocols-->?>

# Author comment: Author response to Decision Letter 2

**DOI:** 10.1371/journal.pntd.0013822.r004

2 Sep 2026

Attachment Submitted filename: Response to the Editor and Reviewers.docx

# Peer review: Decision Letter 2

**DOI:** 10.1371/journal.pntd.0013822.r005

**Authors:** Mathieu Picardeau, Mohammad Jokar

16 Sep 2026

Response to Reviewers Revised Manuscript with Track Changes Manuscript

Shaden Kamhawi

co-Editor-in-Chief

PLOS Neglected Tropical Diseases

orcid.org/0000-0003-4304-636XX

Paul Brindley

co-Editor-in-Chief

PLOS Neglected Tropical Diseases

orcid.org/0000-0003-1765-0002

**Journal Requirements:**

1) Please provide an Author Summary. This should appear in your manuscript between the Abstract (if applicable) and the Introduction, and should be 150-200 words long. The aim should be to make your findings accessible to a wide audience that includes both scientists and non-scientists. Sample summaries can be found on our website under Submission Guidelines:

https://journals.plos.org/plosntds/s/submission-guidelines#loc-parts-of-a-submission

2) Please upload all main figures as separate Figure files in .tif or .eps format. For more information about how to convert and format your figure files please see our guidelines:

https://journals.plos.org/plosntds/s/figures

3) We have noticed that you have uploaded Supporting Information files, but you have not included a list of legends. Please add a full list of legends for your Supporting Information files after the references list.

4) In the online submission form, you indicated that All data underlying the findings of this systematic review and meta-analysis are derived from previously published studies and are fully available within the original articles included in the review. No new primary data were generated or collected for this study. The extracted dataset used for quantitative synthesis, along with statistical code and analysis outputs, can be made available from the corresponding author upon reasonable request. All source studies are publicly accessible through bibliographic databases including PubMed, Scopus, Web of Science, and the Cochrane Library.. All PLOS journals now require all data underlying the findings described in their manuscript to be freely available to other researchers, either

1. In a public repository

2. Within the manuscript itself

3. Uploaded as supplementary information.

This policy applies to all data except where public deposition would breach compliance with the protocol approved by your research ethics board. If your data cannot be made publicly available for ethical or legal reasons (e.g., public availability would compromise patient privacy), please explain your reasons by return email and your exemption request will be escalated to the editor for approval. Your exemption request will be handled independently and will not hold up the peer review process, but will need to be resolved should your manuscript be accepted for publication. One of the Editorial team will then be in touch if there are any issues.

5) As required by our policy on Data Availability, please ensure your manuscript or supplementary information includes the following:

- A numbered table of all studies identified in the literature search, including those that were excluded from the analyses.

- For every excluded study, the table should list the reason(s) for exclusion.

- If any of the included studies are unpublished, include a link (URL) to the primary source or detailed information about how the content can be accessed.

- A table of all data extracted from the primary research sources for the systematic review and/or meta-analysis. The table must include the following information for each study:

- Name of data extractors and date of data extraction

- Confirmation that the study was eligible to be included in the review.

- All data extracted from each study for the reported systematic review and/or meta-analysis that would be needed to replicate your analyses.

- If data or supporting information were obtained from another source (e.g. correspondence with the author of the original research article), please provide the source of data and dates on which the data/information were obtained by your research group.

- If applicable for your analysis, a table showing the completed risk of bias and quality/certainty assessments for each study or outcome.  Please ensure this is provided for each domain or parameter assessed. For example, if you used the Cochrane risk-of-bias tool for randomized trials, provide answers to each of the signalling questions for each study. If you used GRADE to assess certainty of evidence, provide judgements about each of the quality of evidence factor. This should be provided for each outcome.

- An explanation of how missing data were handled.

This information can be included in the main text, supplementary information, or relevant data repository. Please note that providing these underlying data is a requirement for publication in this journal, and if these data are not provided your manuscript might be rejected.

**Reviewers' comments:**

**Key Review Criteria Required for Acceptance?**

As you describe the new analyses required for acceptance, please consider the following:

**Methods**

-Are the objectives of the study clearly articulated with a clear testable hypothesis stated?

-Is the study design appropriate to address the stated objectives?

-Is the population clearly described and appropriate for the hypothesis being tested?

-Is the sample size sufficient to ensure adequate power to address the hypothesis being tested?

-Were correct statistical analysis used to support conclusions?

-Are there concerns about ethical or regulatory requirements being met?

Reviewer #2: The revised study design is now much clearer and the decision to restrict the quantitative synthesis to randomized controlled trials has strengthened the methodological consistency of the work. My main remaining concern is that the adverse event analysis still uses a fixed effect model despite reporting moderate heterogeneity, so a random effects sensitivity analysis should be added because the safety interpretation may change. The PRISMA numbers also need a simple correction, since the text says 43 full texts were assessed and 33 were excluded, but the listed exclusion categories add up to 34, which correctly leaves 9 included studies. Please also recheck the therapeutic failure P value, because the reported value appears inconsistent with the z statistic shown in the forest plot.

Reviewer #5: -

**********

**Results**

-Does the analysis presented match the analysis plan?

-Are the results clearly and completely presented?

-Are the figures (Tables, Images) of sufficient quality for clarity?

Reviewer #2: The results are generally presented clearly and now match the revised analysis plan well, including the use of risk ratios for binary outcomes and mean difference for time to defervescence. One numerical inconsistency remains, as the therapeutic failure forest plot shows a z value of 2.86, which corresponds to a two sided P value around 0.004, while the manuscript reports P less than 0.001, so this should be verified and made uniform across the abstract, results, tables, and figures. The tables and figures are mostly of sufficient quality, but the separately uploaded figure files have labelling errors, because the treatment failure file is labelled Figure 1 and the time to defervescence file is labelled Figure 3, even though the manuscript refers to these analyses as Figures 2 and 5

Reviewer #5: -

**********

**Conclusions**

-Are the conclusions supported by the data presented?

-Are the limitations of analysis clearly described?

-Do the authors discuss how these data can be helpful to advance our understanding of the topic under study?

-Is public health relevance addressed?

Reviewer #2: The conclusions are now much better aligned with the evidence, and the authors appropriately describe the overall relapse finding as statistically non significant, which reflects the revised analysis. Public health relevance is addressed in a reasonable way, and the limitations section is considerably improved. However, the method used to calculate the fail safe N is still not named anywhere in the statistical analysis, so the authors should either specify the exact procedure or remove that discussion from the limitations. A final language pass would also help smooth out a few awkward sentences in the discussion and limitations, but the core scientific message is now appropriate.

Reviewer #5: -

**********

**Editorial and Data Presentation Modifications?**

Use this section for editorial suggestions as well as relatively minor modifications of existing data that would enhance clarity. If the only modifications needed are minor and/or editorial, you may wish to recommend “Minor Revision” or “Accept”.

Reviewer #2: Several reference and naming issues still need correction before publication. The methods cite PRISMA 2020 as reference 9 and GRADE as reference 11, but those reference numbers point to unrelated studies, and several clinical trials appear twice in the reference list. The corresponding author name should be standardized to Karo Servatyari according to the response letter, but the manuscript still says Karo Servatyar. Table 4 still describes the evidence setting as including prospective comparative studies, even though the final synthesis is now restricted to randomized trials. A few unclear expressions in the discussion and limitations also need copyediting, such as the phrases about fluoroquinolone marketing, intracellular time coating, and choppy translation.

Reviewer #5: -

**********

**Summary and General Comments**

Use this section to provide overall comments, discuss strengths/weaknesses of the study, novelty, significance, general execution and scholarship. You may also include additional comments for the author, including concerns about dual publication, research ethics, or publication ethics. If requesting major revision, please articulate the new experiments that are needed.

Reviewer #2: This second revision is substantially improved, and I do not believe another major revision is necessary. The main scientific issue that still concerns me is the fixed effect analysis for adverse events, which may influence how readers interpret safety, so a random effects sensitivity analysis should be presented and the conclusions adjusted if needed. After correcting that issue along with the PRISMA arithmetic, the P value discrepancy, the reference numbering, the author name, and a few minor wording problems, I would be comfortable recommending acceptance without further substantive review.

Reviewer #5: -

**********

PLOS authors have the option to publish the peer review history of their article (what does this mean?). If published, this will include your full peer review and any attached files.

If you choose “no”, your identity will remain anonymous but your review may still be made public.

**Do you want your identity to be public for this peer review?** For information about this choice, including consent withdrawal, please see our Privacy Policy.

Reviewer #2: **Yes:**  Dr.Arman Abdous (DVM,MPH)

Reviewer #5: No

**Figure resubmission:**

**Reproducibility:** -->To enhance the reproducibility of your results, we recommend that authors of applicable studies deposit laboratory protocols in protocols.io, where a protocol can be assigned its own identifier (DOI) such that it can be cited independently in the future. Additionally, PLOS ONE offers an option to publish peer-reviewed clinical study protocols. Read more information on sharing protocols at https://plos.org/protocols?utm_medium=editorial-email&utm_source=authorletters&utm_campaign=protocols-->?>

# Author comment: Author response to Decision Letter 3

**DOI:** 10.1371/journal.pntd.0013822.r006

22 Sep 2026

Attachment Submitted filename: Response to the Reviewers.docx

# Peer review: Decision Letter 3

**DOI:** 10.1371/journal.pntd.0013822.r007

**Authors:** Mathieu Picardeau, Mohammad Jokar

4 Oct 2026

Dear Dr servatyari,

We are pleased to inform you that your manuscript 'Efficacy and Safety of Fluoroquinolones in the Treatment of Human Brucellosis: A Systematic Review and Meta-Analysis' has been provisionally accepted for publication in PLOS Neglected Tropical Diseases.

Before your manuscript can be formally accepted you will need to complete some formatting changes, which you will receive in a follow up email. A member of our team will be in touch with a set of requests.

Please note that your manuscript will not be scheduled for publication until you have made the required changes, so a swift response is appreciated.

IMPORTANT: The editorial review process is now complete. PLOS will only permit corrections to spelling, formatting or significant scientific errors from this point onwards. Requests for major changes, or any which affect the scientific understanding of your work, will cause delays to the publication date of your manuscript.

Should you, your institution’s press office or the journal office choose to press release your paper, you will automatically be opted out of early publication. We ask that you notify us now if you or your institution is planning to press release the article. All press must be co-ordinated with PLOS.

Thank you again for supporting Open Access publishing; we are looking forward to publishing your work in PLOS Neglected Tropical Diseases.

Best regards,

Mohammad Jokar, DVM

Guest Editor

PLOS Neglected Tropical Diseases

Mathieu Picardeau

Section Editor

PLOS Neglected Tropical Diseases

Shaden Kamhawi

co-Editor-in-Chief

PLOS Neglected Tropical Diseases

orcid.org/0000-0003-4304-636XX

Paul Brindley

co-Editor-in-Chief

PLOS Neglected Tropical Diseases

orcid.org/0000-0003-1765-0002

***********************************************************

p.p1 {margin: 0.0px 0.0px 0.0px 0.0px; line-height: 16.0px; font: 14.0px Arial; color: #323333; -webkit-text-stroke: #323333}span.s1 {font-kerning: none

Reviewer’s Responses to Questions

**Key Review Criteria Required for Acceptance?**

As you describe the new analyses required for acceptance, please consider the following:

**Methods**

-Are the objectives of the study clearly articulated with a clear testable hypothesis stated?

-Is the study design appropriate to address the stated objectives?

-Is the population clearly described and appropriate for the hypothesis being tested?

-Is the sample size sufficient to ensure adequate power to address the hypothesis being tested?

-Were correct statistical analysis used to support conclusions?

-Are there concerns about ethical or regulatory requirements being met?

Reviewer #2: The revised manuscript now presents an appropriate and sufficiently transparent methodology for the stated objective. The eligibility criteria, study selection and extraction procedures, RoB 2 assessment, GRADE evaluation, meta-analytic approach, subgroup analyses, and sensitivity analyses are described with adequate detail, and the authors have satisfactorily addressed the methodological concerns raised during the previous review rounds. I do not identify any remaining methodological issue that would require additional analysis before publication

**********

**Results**

-Does the analysis presented match the analysis plan?

-Are the results clearly and completely presented?

-Are the figures (Tables, Images) of sufficient quality for clarity?

Reviewer #2: The results are now presented in a substantially clearer and more consistent manner, and the major statistical issues raised previously have been adequately addressed. In particular, the therapeutic-failure estimate, relapse analysis, adverse-event analysis using a random-effects model, time-to-defervescence results, sensitivity analyses, and subgroup findings are appropriately reported and interpreted without overstating non-significant associations. The tables and figures provide adequate support for the principal findings, and I do not consider any additional analysis necessary for acceptance

**********

**Conclusions**

-Are the conclusions supported by the data presented?

-Are the limitations of analysis clearly described?

-Do the authors discuss how these data can be helpful to advance our understanding of the topic under study?

-Is public health relevance addressed?

Reviewer #2: The conclusions are appropriately supported by the pooled evidence and are now more cautious and balanced. The authors clearly distinguish the significant increase in therapeutic failure and longer time to defervescence from the non-significant overall findings for relapse and adverse events, while also acknowledging limitations related to older trials, small sample sizes, imprecision, clinical heterogeneity, and limited generalizability.The clinical and public-health relevance is clear, particularly for treatment decisions in brucellosis-endemic settings.

**********

**Editorial and Data Presentation Modifications?**

Use this section for editorial suggestions as well as relatively minor modifications of existing data that would enhance clarity. If the only modifications needed are minor and/or editorial, you may wish to recommend “Minor Revision” or “Accept”.

Reviewer #2: No further substantive revision is required.

**********

**Summary and General Comments**

Use this section to provide overall comments, discuss strengths/weaknesses of the study, novelty, significance, general execution and scholarship. You may also include additional comments for the author, including concerns about dual publication, research ethics, or publication ethics. If requesting major revision, please articulate the new experiments that are needed.

Reviewer #2: (No Response)

**********

PLOS authors have the option to publish the peer review history of their article (what does this mean?). If published, this will include your full peer review and any attached files.

If you choose “no”, your identity will remain anonymous but your review may still be made public.

**Do you want your identity to be public for this peer review?** For information about this choice, including consent withdrawal, please see our Privacy Policy.

Reviewer #2: **Yes:**  Dr.Arman Abdous DVM,MPH

# Editor report: Acceptance letter

**DOI:** 10.1371/journal.pntd.0013822.r008

**Authors:** Mathieu Picardeau, Mohammad Jokar

Dear Dr servatyari,

We are delighted to inform you that your manuscript, "Efficacy and Safety of Fluoroquinolones in the Treatment of Human Brucellosis: A Systematic Review and Meta-Analysis," has been formally accepted for publication in PLOS Neglected Tropical Diseases.

We have now passed your article onto the PLOS Production Department who will complete the rest of the publication process. All authors will receive a confirmation email upon publication.

The corresponding author will soon be receiving a typeset proof for review, to ensure errors have not been introduced during production. Please review the PDF proof of your manuscript carefully, as this is the last chance to correct any scientific or type-setting errors. Please note that major changes, or those which affect the scientific understanding of the work, will likely cause delays to the publication date of your manuscript. Note: Proofs for Front Matter articles (Editorial, Viewpoint, Symposium, Review, etc...) are generated on a different schedule and may not be made available as quickly.

Soon after your final files are uploaded, the early version of your manuscript will be published online unless you opted out of this process. The date of the early version will be your article’s publication date. The final article will be published to the same URL, and all versions of the paper will be accessible to readers.

For Research Articles, you will receive an invoice from PLOS for your publication fee after your manuscript has reached the completed accept phase. If you receive an email requesting payment before acceptance or for any other service, this may be a phishing scheme. Learn how to identify phishing emails and protect your accounts at https://explore.plos.org/phishing.

Thank you again for supporting open-access publishing; we are looking forward to publishing your work in PLOS Neglected Tropical Diseases.

Best regards,

Shaden Kamhawi

co-Editor-in-Chief

PLOS Neglected Tropical Diseases

Paul Brindley

co-Editor-in-Chief

PLOS Neglected Tropical Diseases
